# Relationship Between Hypovitaminosis C, Obesity, and Features of Metabolic Syndrome—A Narrative Review

**DOI:** 10.1093/nutrit/nuaf230

**Published:** 2026-01-20

**Authors:** Robert Beaumont Wilson, Yicong Liang, Devesh Kaushal, Anitra Carr

**Affiliations:** Faculty of Medicine, University of New South Wales (Sydney), Kensington, NSW 2052, Australia; Faculty of Medicine, University of New South Wales (Sydney), Kensington, NSW 2052, Australia; Western Sydney University, Campbelltown Hospital, Sydney, NSW 2560, Australia; Nutrition in Medicine Research Group, Department of Pathology and Biomedical Science, University of Otago, Christchurch, 8140, New Zealand

**Keywords:** antioxidant, ascorbic acid, fatty acid oxidation, free radical, GLUT, inflammation, insulin resistance, Mediterranean diet, metabolic syndrome, micronutrient, NAFLD, obesity, oxidative stress, RDA, ROS, scurvy, SVCT, vitamin C, Western diet

## Abstract

This narrative review aimed to review plausible mechanisms for the role of vitamin C (ascorbic acid [AA]) in the maintenance of healthy weight and energy metabolism; examine the evidence for inadequate vitamin C (plasma AA <50 µmol/L), hypovitaminosis C (≤23 µmol/L), and vitamin C deficiency (≤11.4 µmol/L) in the disrupted homeostasis of obesity and metabolic syndrome; and ascertain whether vitamin C supplementation or dietary intervention could potentially treat obesity and the associated features of metabolic syndrome. Vitamin C hypovitaminosis and deficiency are prevalent in developed countries, despite the widespread availability of vitamin C–containing fruit and vegetables and vitamin supplements. Western diets are characterized by highly processed, macronutrient-rich foods, which are deficient in dietary fiber and micronutrients. This contributes to postprandial oxidative stress and gut dysbiosis, leading to profound effects on insulin sensitivity, hyperglycemia, levels of endotoxemia, fatty acid oxidation, adipocyte hypertrophy, and regulation of metabolism and energy balance. The existing in vitro and in vivo preclinical data demonstrate the effectiveness of vitamin C as both a prophylactic and a therapeutic intervention for obesity and metabolic syndrome. The outcomes in human intervention studies are more modest, with improvements in insulin sensitivity, lipid profile, metabolic inflammation, weight, hypertension, gut permeability, and hepatic steatosis. Some clinical studies are limited by the lack of baseline plasma AA concentrations, or the inability to optimize plasma AA in participants with hypovitaminosis C or metabolic syndrome. The addition of vitamin C to physical activity and dietary interventions may improve the efficacy of treatments for obesity and metabolic dysfunction. However, more data are required to understand the synergism between vitamin C supplementation, medical nutrition therapy, adequate exercise, and pharmacological intervention in weight control and metabolic syndrome management.

## INTRODUCTION

Since the development of mass production of food and increased urbanization, there has been a rapid increase in the global prevalence of obesity and related diseases.^1^ This was originally confined to affluent Western countries but is now found in all socioeconomic strata and most GDP (gross domestic product) distributions. From the National Health and Nutrition Examination Survey (NHANES), between 1961/1962 and 2017/2018 the prevalence of obesity (body mass index [BMI] ≥30 kg/m^2^) in US adults aged 20–75 years increased from 13.4% to 42.8%, and severe obesity (BMI ≥40 kg/m^2^) increased from 0.9% to 9.6%, with 73.1% of the US population currently being either overweight (BMI = 25*–*29.9 kg/m^2^) or obese.^2^ The consumption of energy-dense, high-sugar, high-salt, high-fat diets with minimal dietary fiber (Western or cafeteria diets) results in macronutrient excess with micronutrient deficiency.^2–5^ This contributes to rapid increases in plasma glucose and insulin and postprandial oxidative stress, glycotoxicity, formation of reactive carbonyls, lipogenesis, adipocyte hypertrophy, and inhibition of fatty acid oxidation.^6^^,^^7^ As a result, unhealthy diets and sedentary lifestyles lead to systemic inflammation, endothelial dysfunction, insulin resistance, obesity, and metabolic syndrome (MetS).^1^^,^^8^^,^^9^ This narrative review aimed to review plausible mechanisms for the role of vitamin C (ascorbic acid [AA]) in the maintenance of healthy weight and energy metabolism; examine the evidence for inadequate vitamin C (plasma AA <50 µmol/L), hypovitaminosis C (≤23 µmol/L), and vitamin C deficiency (≤11.4 µmol/L) in the disrupted homeostasis of obesity and MetS; and ascertain whether vitamin C supplementation or dietary intervention could potentially treat obesity and the components of MetS.^10–21^

## METHODS

A literature search of PubMed, Medline, and Cochrane Library databases was conducted using MeSH (Medical Subject Heading) key word terms “vitamin C” OR “ascorbic acid” AND “obesity” in combination with “diet,” “pharmacology,” “deficiency,” “oxidative stress,” “oxygenase,” “carnitine,” “epigenome,” “DNA methylation,” “type 2 diabetes mellitus,” “non-alcoholic fatty liver disease,” “adipokine,” “adiponectin,” “leptin,” “glucose metabolism disorder,” “metabolic syndrome,” “ferroptosis,” “hypertension,” “DASH diet,” “Mediterranean diet,” “bariatric surgery” OR “endotoxin.” Search results were limited to the English language. The titles and abstracts of the search results were screened. The full texts that may be relevant were read and compiled, and a narrative review was conducted.

## PREVALENCE OF HYPOVITAMINOSIS C AND DEFICIENCY

Despite the apparent widespread availability of fresh fruit and vegetables and oral vitamin supplements, AA hypovitaminosis and deficiency are still observed in high-income countries, and highly prevalent in low- and middle-income countries.^18–21^ In a nationally representative survey of Canadian adults aged 20 to 79 years (*n* = 1615), the overall prevalence of AA deficiency (<11 μmol/L) was 2.9%. The prevalence of AA deficiency was higher among smokers (10%), people who rarely or never consumed citrus fruit (13%), or those who rarely or never drank 100% fruit juice (7%). Of the sampled household population, 25.6% were obese (BMI >30 kg/m^2^), which was significantly associated with lower AA plasma concentrations compared to those who were neither obese nor overweight (*P* < .05).^22^ This was consistent with a 2008 study conducted in a Canadian university teaching hospital of 141 outpatients (with otherwise normal hematological and biochemical parameters), which found that 13% had a subnormal AA concentration (11.4*–*28.4 μmol/L) and 3% were AA deficient (<11.4 μmol/L). By contrast, 60% of 149 inpatients who had been acutely admitted to the medical ward in the same hospital had a subnormal AA concentration and 19% were AA deficient. The authors emphasized the importance of correct blood sample collection, handling, transportation, storage, and analysis in avoiding spuriously low plasma AA results.^23^^,^^24^ The prevalence of AA hypovitaminosis or deficiency in the Canadian population was lower than that reported in studies of predominantly healthy or randomly selected groups from high-income countries, including England, Scotland, the United States, France, Finland, and Singapore.^18^ In an Australian study of 12 934 individuals (hospital inpatients and outpatients combined) who underwent AA testing between 2017 and 2021, 3168 (24.5%) were vitamin C deficient (serum AA <11 μmol/L), 3872 (29.9%) had vitamin C hypovitaminosis (serum AA = 12–39 μmol/L), and 5894 (45.6%) had normal serum AA concentrations (40–250 μmol/L).^25^

Apart from obesity and MetS, other contributing factors to AA hypovitaminosis and deficiency include demographic (advanced age, male sex, race) and environmental (smoking, pollution, extreme northern latitude geography) characteristics, physiological states (pregnancy and lactation), pathological states (acute infection/hospitalization, chronic illness, psychiatric illness, renal dialysis, malabsorption, malignancy), dietary characteristics (fad, restrictive, staple, cafeteria or Western diets, seasonal variation), socioeconomic characteristics (food deserts, food insecurity, social isolation, remote location, socioeconomic disadvantage) and malnutrition.^18–21^ In the 2017–2018 US NHANES study of 4932 adults aged 20 years or older, the prevalence of vitamin C deficiency (serum AA <11.4 µmol/L) was 6.8% (95% CI, 5.0%–9.0%). On analysis of significant risk factors, mean serum AA was 10 μmol/L lower in men (46.1 μmol/L) vs women (56.0 μmol/L), 8 μmol/L lower in persons with low incomes (46.3 μmol/L) vs high incomes (54.2 μmol/L), 11 μmol/L lower in persons with obesity (BMI ≥30 kg/m^2^, 45.4 μmol/L) vs healthy weight (BMI 18.5–24.9 kg/m^2^, 56.1 μmol/L), 15 μmol/L lower in smokers (39.9 μmol/L) vs nonsmokers (54.7 μmol/L), and 22.1 μmol/L less in individuals in the lowest dietary AA intake quartile (<20 mg AA/d, 41.2 μmol/L) vs the highest dietary AA intake (>110 mg AA/d, 63.3 μmol/L).^26^

### Definition of MetS

The revised National Cholesterol Education Program Expert Panel on Detection, Evaluation, and Treatment of High Blood Cholesterol in Adults (NCEP ATP III) definition of MetS is met when 3 of the 5 following criteria are present:

1. Central obesity: waist circumference (WC): adult Caucasian male >102 cm or female >88 cm
2. Hypertriglyceridemia: triglyceride ≥150 mg/dL (1.7 mmol/L)
3. Dyslipidemia: high-density-lipoprotein (HDL) cholesterol <40 mg/dL (1.03 mmol/L) for men or <50 mg/dL (1.3 mmol/L) for women
4. Hypertension: systolic/diastolic blood pressure ≥130/≥85 mm Hg
5. Glucose intolerance: fasting plasma glucose ≥100 mg/dL (5.6 mmol/L)

The International Diabetes Federation (IDF) definition differs in that central obesity (assumed if BMI is >30 kg/m^2^) is a prerequisite for MetS, and includes an ethnicity-specific WC: adult Asian male >90 cm or Asian female >80 cm.^27^ The consequences of the modern obesity epidemic and untreated MetS include prevalent hypertension, dyslipidemia, obstructive sleep apnea (OSA), cardiovascular disease, type 2 diabetes (T2D), nonalcoholic steatohepatitis (NASH), cancer, epigenetic aging, and premature death.^28–33^

## ASCORBIC ACID PHYSIOLOGY AND DAILY REQUIREMENTS

Vitamin C, also known as l-ascorbic acid, is a water-soluble vitamin that participates in a wide range of reduction/oxidation (redox) activities in biological systems.^26^^,^^34–42^ Humans are unable to synthesize AA due to a loss-of-function mutation in the gene for l-gulono-γ-lactone oxidase (*GLO*). *GLO* encodes the hepatic enzyme essential for the final step in the conversion of glucose to AA—the oxidation of l-gulono-1,3-lactone to l-ascorbic acid.^39^ Humans are auxotrophic for AA, and dietary consumption is thus essential. Some other animals, including nonhuman higher primates, capybara, guinea pigs, fruit-eating bats, Passeriformes bird, and teleost fish species, are also unable to endogenously synthesize AA.^36^ Animal models, including guinea pigs, senescence marker protein-30 (SMP30)/gluconolactonase (GNL) knockout mice, osteogenic disorder Shionogi (ODS) rats, osteogenic disorder (OD) Danish pigs with congenital deficiency in *GLO* (*od/od* pigs), and *GLO* knockout (*GLO* KO) mice, are used to study the effects of AA deficiency.^39^ Murine models of high-fat-diet or cafeteria (Western-type) diet–induced obesity using C57BL/6J (non-auxotrophic) mice resemble human obesity, as both develop low-grade chronic inflammation, oxidative stress, intestinal dysbiosis, endotoxemia, MetS, and age-related obesity.^43^

### Ascorbic Acid Absorption and Transport

Ascorbate is actively absorbed across the apical brush border in the small intestine by the low-affinity/high-capacity, dose-dependent, saturable sodium-linked vitamin C transporter 1 (SVCT1) with a V_max_ of 15 pmol/minute/cell and *K*_m_ of 65–252 µM.^44^ The oxidized form of AA is dehydroascorbate (DHA), which is absorbed via facilitated diffusion by glucose transporters (GLUT1 or GLUT3) on the apical membrane and (GLUT1 and GLUT2) on the basolateral membrane.^44^ Export of AA is handled by the high-affinity (*K*_m_ ≈8–69 μmol/L) but low-capacity (V_max_ =1 pmol/min/cell) sodium-dependent urate transporter SVCT2. SVCT2 is found in most tissues and enables transport from the blood to occur against a large concentration gradient, achieving very high AA intracellular concentrations in the brain (2–10 mmol/L), leukocytes (1.4–3.8 mmol/L), adrenal glands (4–10 mmol/L), and skeletal muscle (0.2–0.4 mmol/L).^45–47^ Transport of glucose and DHA by GLUTs is characterized by reciprocal competitive inhibition, as both molecules are transported by GLUT1, GLUT3, GLUT4, and GLUT10.^47^^,^^48^ In vitamin C auxotrophs, mature erythrocytes lack SVCT1 and SVCT2, and thus are wholly reliant on GLUT1 for DHA to be transported into the erythrocyte and then rapidly converted back to AA by intracellular glutathione (GSH), or by glutathione- or nicotinamide adenine dinucleotide (phosphate), reduced form (NAD[P]H)- linked enzymes.^35^^,^^39^ This represents an important antioxidant mechanism by which oxidized vitamin C (DHA) can be regenerated in AA auxotrophic animals.^44^ Indeed, the structural integrity of erythrocytes requires the AA-dependent cytoskeleton protein β-spectrin, a lack of which contributes to erythrocyte membrane rigidity, fragility under shear stress, and hemolytic jaundice in advanced human scurvy.^47^ Because human erythrocytes lack mitochondria (and sodium-glucose linked transporters [SGLTs]), they are reliant on a continuous supply of glucose by insulin-independent transmembrane GLUT1 transport for glycolysis-generated ATP and nicotinamide adenine dinucleotide, reduced form (NADH), production, and for the pentose phosphate pathway (PPP) for reducing elements (NAD[P]H).^45–48^ Intestinal ascorbate transport (SVCT1) is inhibited by low lumenal pH or absent lumenal sodium, and DHA transport (GLUT1) by elevated plasma or intestinal brush border membrane vesicle glucose concentrations (>5 mmol/L). As a water-soluble vitamin, ascorbate is freely filtered by the renal glomeruli and reabsorbed in the proximal renal tubules by SVCT1. The expression of SVCT1 and SVCT2 is under substrate regulation, which means large individual doses of vitamin C result in downregulation of SVCT transporters, as compared with divided doses. It has been suggested that this may contribute to rebound scurvy if high-dose AA supplementation is abruptly ceased.^49^

### Ascorbic Acid Plasma Concentrations and Definitions

Recognized definitions for AA plasma concentrations include saturation (≥70 µmol/L), adequate (≥50 µmol/L), inadequate (<50 µmol/L), hypovitaminosis C (≤23 µmol/L), and deficiency (≤11.4 µmol/L).^10–18^ Plasma AA concentrations less than 5 µmol/L are below the equipment detection level in some laboratories and are thus designated as “unrecordable.” Patients who have vitamin C inadequacy will actively reabsorb filtered vitamin C from the renal tubules, and as such, the mean renal threshold at which AA starts to appear in the urine in healthy men is at a plasma AA concentration of 48.5 ± 5.2 µmol/L and in women at 58.3 ± 7.5 µmol/L.^11^ However, saturation of renal tubular SVCT1 reabsorption is not equivalent to AA tissue saturation, due to the different affinity and kinetics of vitamin C transporters and considerable variation between patients. This is reflected by the AA “minimal elimination threshold” or “renal leak” being set as 2 SDs lower than the average renal threshold (ie, <38 µmol/L for males and <43 µmol/L for females).^11^ Chronic deficiency of vitamin C (plasma concentration ≤11.4 µmol/L) classically results in scurvy.^47^

### Hypovitaminosis C and Obesity

**Figure 1.**
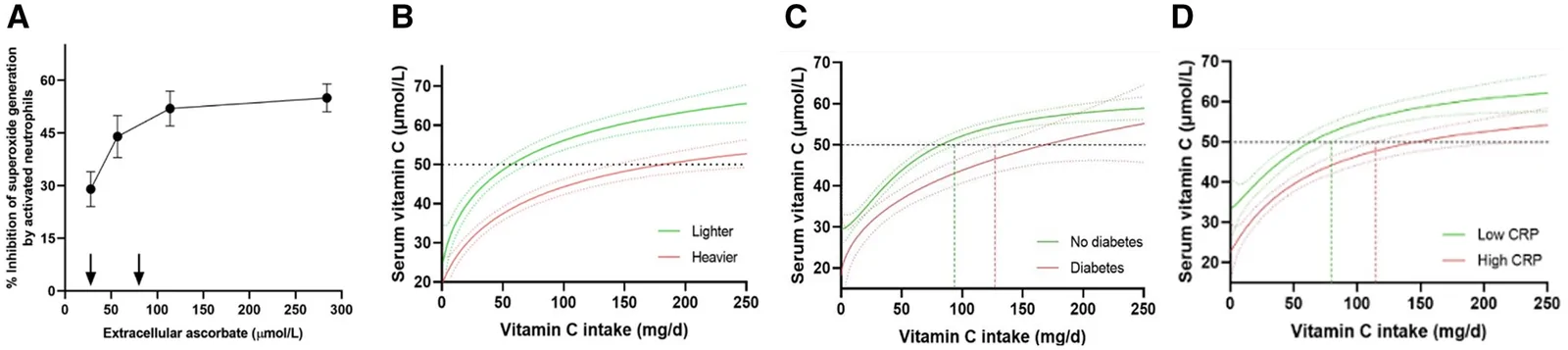
(A) Effect of Varying Extracellular Ascorbate Concentrations on Scavenging of Superoxide Produced by Activated Neutrophils (1 × 10^6^ neutrophils/mL). Arrows indicate the range of normal human plasma ascorbate concentrations (ie, 22–85 µmol/L) (US Institute of Medicine Panel on Dietary Antioxidants and Related Compounds, 2000). Reproduced from Carr and Lykkesfeldt^53^ with permission. (B) Vitamin C dose–response relationship relative to body weight in the NHANES cohort. The lighter weight (median [IQR]) group (63 [57, 68] kg; *n*=932) reached 50 µmol/L serum vitamin C concentrations at an oral vitamin C intake of 56 (45, 70) mg/day, whereas the heavier group (105 [67, 118] kg; *n*=930) required 177 (140, not attainable (ie, did not reach 50 µmol/L [NA]) mg/day. Sigmoidal (4 parameter logistic) curves were fitted to the dose–concentration data with asymmetrical 95% CIs indicated by dotted green and red curves. The dashed black horizontal line indicates 50 µmol/L serum vitamin C, which is considered adequate. Reproduced from Carr and Lykkesfeldt^52^ with permission. (C). Serum vitamin C concentrations relative to vitamin C intake stratified by diabetes status in the NHANES cohort. No-diabetes group (green line), *n* = 2340; diabetes group (red line), *n* = 488. Sigmoidal (4-parameter logistic) curves were fitted to the dose–concentration data with asymmetrical 95% CIs indicated by dotted green and red curves. The dashed black horizontal line indicates 50 µmol/L serum vitamin C, which is considered adequate. The vertical dashed green line corresponds to the upper limit of the 95% CI for oral vitamin C intake in the no-diabetes group (93 mg/d) to achieve an adequate serum vitamin C concentration. The vertical dashed red line corresponds to the lower limit of the 95% CI for oral vitamin C intake (126 mg/d) in the diabetes group to achieve an adequate serum vitamin C concentration. Reproduced from Carr et al.^13^ with permission. (D) Impact of CRP concentrations on the vitamin C dose–concentration relationship in the NHANES cohort. Low CRP (<1 mg/L; green line), *n* = 765; high CRP (>3 mg/L; red line), *n* = 1081. The low-CRP group had a median weight of 70 (61, 82) kg and BMI of 25 (22, 28) kg/m^2^; the high-CRP group had a median weight of 89 (75, 108) kg and BMI of 33 (28, 38) kg/m^2^. Sigmoidal (4-parameter logistic) curves were fitted to the dose–concentration data with asymmetrical 95% CIs indicated by dotted green and red curves. The dashed black horizontal line indicates 50 µmol/L serum vitamin C, which is considered adequate. The vertical dashed green line corresponds to the upper limit of the 95% CI for oral vitamin C intake in the low-CRP group (79 mg/d) to achieve an adequate serum vitamin C concentration. The vertical dashed red line corresponds to the lower limit of the 95% CI for oral vitamin C intake (114 mg/d) in the high-CRP group to achieve an adequate serum vitamin C concentration. Abbreviations: BMI, body mass index; CRP, C-reactive protein; NHANES, US National Health and Nutrition Examination Survey. Reproduced from Carr et al.^13^ with permission.

Inadequate micronutrient intake (including AA), impaired intestinal AA absorption, gut microbial dysbiosis, volumetric dilution, changes in AA partitioning between lean and adipose tissue, increased utilization of AA due to oxidative stress, or increased renal losses are thought to contribute to the prevalence of hypovitaminosis C in people who are overweight (BMI = 25*–*29.9 kg/m^2^) or obese (BMI ≥30 kg/m^2^), particularly those with T2D or MetS.^10–17^^,^^50–55^ From the 2017–2018 NHANES cohort, participants in the lighter weight tertile (63 [57, 68] kg), *n *= 932 reached 50-µmol/L AA plasma concentrations at an AA intake of 56 (45, 70) mg per day, whereas in the heavier weight tertile (105 [67, 118] kg), *n *= 930 required 177 (140, not attainable (ie, did not reach 50 µmol/L [NA])) mg per day. The participants with diabetes had lower plasma AA concentrations (median [IQR]) than those without diabetes (38 [17, 52] µmol/L vs 44 [25, 61] µmol/L; *P *< .0001), despite comparable AA dietary intakes between the 2 groups (51 [26, 93] vs 53 [24, 104] mg/d; *P *= .5).^13^ For the total cohort (*n *= 2828), an estimated AA intake of 97 [85, 106] mg per day was required to achieve adequate AA plasma concentrations (≥50 µmol/L); the group with diabetes (*n *= 488, type 1 diabetes [T1D] = 17, T2D = 471) required 166 (126, NA) mg AA per day; the nonsmokers in the diabetes group (*n* = 378) required 145 (102, NA) mg AA per day; and the group without diabetes (*n *= 2340) required 81 (72, 93) mg AA per day.^13^  **(****Figure 1**).

In a 2022 retrospective study comparing T1D (*n *= 73) and T2D (*n *= 63), patients with T2D had a higher proportion of AA insufficiency (95% <50 μmol/L), hypovitaminosis (68% ≤23 μmol/L), and deficiency (38% ≤11 μmol/L), which was influenced by greater body weight and renal dysfunction in patients with T2D compared to those with T1D.^12^ In a study of patients with diabetic renal impairment, those with clinical nephropathy had lower mean plasma AA (*P *= .0009) and higher renal clearance of AA (*P *= .005) than those with microalbuminuria.^51^ Dietary intake of vitamin C strongly influences plasma AA concentrations in healthy-weight persons without metabolic dysfunction but has less of an effect in individuals with hypovitaminosis C, T2D, obesity, or metabolic inflammation (metaflammation; elevated C-reactive protein [CRP]).^12–17^ A renal leak of AA may contribute to estimated daily AA losses of between 0.24 and 24 mg in T2D.^50^ Systemic inflammation (CRP >3mg/L) in patients with obesity/T2D and increasing visceral adiposity is associated with an additional requirement of 35 mg/day of AA, compared with those with low-risk CRP concentrations (<1 mg/L).^13^ There was a strong correlation between body weight and CRP concentrations in the total NHANES cohort (*r *= 0.408, *P *< .0001), corresponding to a 0.84-mg/L increase in CRP for every 10 kg in weight gain^13^ (**Figure 1**).

### Ascorbic Acid Deficiency and Scurvy

Vitamin C is a cofactor required by procollagen prolyl and lysyl hydroxylases to hydroxylate proline and lysine residues within procollagen during collagen synthesis and formation of its stable triple helical structure. Scurvy is a pathological syndrome characterized by AA deficiency and defective collagen production, poor wound healing, hair loss, capillary fragility, easy bruising, anemia, and skin, joint, periosteal, visceral, and mucosal hemorrhage. Frank scurvy tends to manifest at vitamin C plasma concentrations below 3–5 µmol/L, but this can vary between individuals. Early manifestations of scurvy may be nonspecific and include generalized malaise, unexplained fatigue, musculoskeletal aches, poor appetite, weight loss, irritability, avoidance of exercise, and hypochondriasis.^45–47^ Levine et al ^45^ showed that fatigue occurred when plasma AA concentrations were less than approximately 20 *μ*mol/L. As the disease progresses, pathognomonic features of scurvy emerge, including gingival bleeding, discomfort, and swelling; corkscrew hair formation; hyperkeratosis; and perifollicular petechiae, together with fever, confusion, anemia, shortness of breath, and infections.^34^^,^^45–47^ Other connective tissue proteins that require AA for biosynthesis include proteoglycans, elastin, bone matrix, fibronectin, and elastin-associated fibrillin. Loss of these extracellular matrix proteins may also contribute to the poor bone, skin, teeth, and gum health of patients presenting with scurvy.^41^

### Recommended Daily Allowances of AA

The provision of as little as 10 mg/day of AA was reported to prevent scurvy in the UK double-blind controlled trial of human vitamin C depletion.^53^^,^^56^ This was conducted between October 1944 and February 1946 at the Sorby Research Institute in Sheffield on 20 healthy participants (19 males, 1 female) who were mainly conscientious objectors to military service during World War II.^56^ The participants were assigned to receive 0, 10, or 70 mg of AA per day for an average of 9 months. They then had experimental skin wounds created, which were excised 3 weeks later, and the scar breaking strength was measured. Some of the AA-depleted participants were repleted with daily oral AA to study the dose–response in wound healing. The results of this experiment have recently been re-analyzed by robust parametric analysis, and it was found that repletion with an average dose of 75 mg/day of AA for 6 months failed to restore normal scar strength in the AA-depleted participants. In their review, Hujoel and Hujoel^56^ found that an average daily oral AA intake of 95 mg is required to prevent weak scar strength for 97.5% of the population, and in the Sorby study, for each additional 10 mg vitamin C intake above 10 mg/day, the scar strength increased by 2.9 Newtons (*P* < .02). This challenges the World Health Organization’s (WHO’s) later position that 10 mg AA per day was sufficient to prevent defective wound healing related to scurvy. Hujoel and Hujoel^56^ also emphasized that, in the Sorby study, 10 mg/day of AA did not prevent the onset of physical fatigue, intra-oral capillary weakness, and follicular hyperkeratosis. These findings have profound implications for current national and international AA Recommended Daily Allowance (RDA) guidelines.^53^

Indeed, the British Medical Research Council’s 1948 RDA of 30 mg of AA/day for adults was questioned in 1957 by Dr Erik Uhl of the Danish National Public Health Service in Copenhagen.^57^ He suggested that the AA RDA should be at least 10 times higher than the minimum requirement from the Sorby experiment (AA/d = 5–10 mg × 10) to prevent macroscopic features of scurvy and resist infection or bacterial toxins such as diphtheria toxin. This would be in accordance with the US National Health Research Council’s AA RDA of 75 mg/day in healthy adults set in 1953. It would not be until 1969 that the results of the US vitamin C depletion studies in Iowa State prisoners were published: the trial conclusions were similar to those of the Sorby experiment, but involved participants who were already vitamin C depleted before the trial began, and hence they developed scorbutic symptoms within 4 weeks of experimental vitamin C deprivation.^58^ The US Institute of Medicine (IOM) was established in 1970, and in 1974, the North American AA RDA was lowered from 60 to 45 mg/day for adult males and females; this was a dose that was believed to prevent scurvy for 30–45 days if all dietary vitamin C intake was ceased.^58–60^

Apart from extracellular matrix protein synthesis, AA is also required for maintaining redox status; free radical scavenging; lipid metabolism; carnitine, catecholamine, and neurotransmitter synthesis; epigenetic regulation of DNA translation; mitochondrial function; non-heme iron absorption; leucocyte phagocytosis and microbial killing.^38^ Vitamin C deficiency is implicated in the development or progression of multiple health conditions, including anemia, obesity, hypertension, cardiovascular disease, and cancer.^38^ Patients with obesity or diabetes, smokers, and older patients require greater daily amounts of AA to maintain adequate plasma AA levels (≥50 µmol/L)^61^ (**Figure 1**). There are several existing national, societal, and international guidelines for the RDA of AA, but none provide guidance on individuals with obesity.^53^^,^^62–64^ The 2004 WHO and Australasian RDA for vitamin C is 45 mg/day, and the 1991 UK Department of Health RDA is 40 mg/day for men and women, which were originally developed to prevent scurvy. These recommendations were based on the findings of the Sorby and Iowa experiments, but do not provide adequate wound healing or protection against oxidative stress or chronic diseases.^64^ In 2000, the US IOM increased the 1989 AA RDA of 60 mg/day to 90 mg/day for men, and to an extrapolated RDA (based on weight) of 75 mg/day for women.^65^ The IOM stated that “antioxidant protection is increasingly provided as ascorbate concentrations increase with the greatest change in protection seen for ascorbate concentrations between 28 and 57 μmol/L.”^65^ However, the ability to scavenge superoxide generated by the respiratory burst in activated neutrophils approached the asymptote at plasma ascorbate concentrations between 57 and 114 µmol/L (**Figure 1**).^53^^,^^56^^,^^65^ In 2001, Padayatty and Levine provided evidence that the extrapolated AA RDA of 75 mg/day for healthy women was inadequate.^46^ This was due to the steep linear portion of the sigmoidal dose–response between oral AA and plasma AA concentrations at oral AA doses between 30 and 100 mg/day, with wide SDs in response to small changes in oral AA intake; the first AA dose at which the curve flattened was at 100 mg/day; and AA doses greater than 100 mg/day would achieve plasma concentrations at which SVCT2 should function at or near V_max_.^45–47^

The 2013 European Food Safety Authority (EFSA) Population Reference Intake (PRI); the 2015 Nutrition Societies of Germany, Austria, and Switzerland (DACH) vitamin C Reference Intake (RI); and the 2023 Nordic Nutrition Recommendation (NNR) RI is 110 mg/day of AA for men and 95 mg/day for women, with an extra 40 mg/day of AA required in smokers.^9^^,^^66^ This was based on data from nonsmoking healthy adult males (mean weight, 70.7 kg), with assumed metabolic AA losses of 50 mg/day, a bioavailability of 80%, and urinary excretion of 25% of an oral vitamin C dose, to achieve a fasting ascorbate plasma level of 50 μmol/L. The AA reference value (RI) of 110 mg/day was thus derived from the Estimated Average Requirement (EAR) in men of 91 mg/day plus a coefficient of variation of 10% × 2. The lower dose for women was based on a smaller lean body mass (60.0 kg), total body water, and body size. The French Agency for Food Safety (AFSSA) based their 2001 AA PRI of 110 mg/day in men and women on a 3-day dietary recall and data from the French SU.VI.MAX cohort study.^66^ This showed that steady-state plasma values of 64 ± 1 μmol/L in women and 56 ± 3 μmol/L in men were achieved with AA oral intakes of 85.4 and 85.9 mg/day for men and women, respectively. A theoretical SD of 15% × 2 was added to obtain an AA PRI of 110 mg/day for both sexes. The AFSSA PRI for men and women older than 75 years of age was set at 120 mg/day, based on higher AA requirements in older people and considerations of immunity, cardiovascular risk, cancer risk, and cognition.^9^^,^^61^^,^^66^

The 2006 Australian National Health and Medical Research Council’s suggested dietary target (SDT) of AA for the prevention of chronic or noncommunicable diseases is much higher at 220 mg/day for men and 190 mg/day for women.^64^ This intake was to be attained by replacing nutrient-poor, energy-dense foods and drinks with “plenty of vegetables, legumes and fruit.”^64^ Obesity is more closely associated with inadequate plasma AA concentrations than dietary intake of AA.^53^^,^^62–64^^,^^66^^,^^67^ Daily AA intakes of between 200 and 400 mg will generally achieve a plasma AA saturation of 70 µmol/L in healthy individuals, due to its nonlinear dose distribution.^45–47^ To achieve adequate AA plasma concentrations (≥50 µmol/L), individuals whose weight exceeds 100 kg require oral intakes of more than 155 mg AA/day, compared with the general population (110 mg AA/d) and smokers (165 mg AA/d)^52^ (**Figure 1**).

Adequate plasma AA concentrations (≥50 µmol/L) are associated with normal or improved (1) insulin sensitivity and glucose tolerance; (2) lipid profile and body fat composition; (3) fatty acid β-oxidation; (4) systemic inflammation; (5) adiponectin release; (6) antioxidant defenses; (7) glycosylation markers; (8) protection from oxidative, glycative, and carbonyl stress; and (9) prevention of fasting and postprandial oxidative stress.^26^^,^^34–42^

Saturating plasma levels of AA (>70 µmol/L) obtained with more than 200 mg/day of oral AA are required for optimal neutrophil chemokinesis and chemotaxis, as well as cardiovascular protection from endothelial dysfunction.^68^ Ascorbic acid is required for microtubule assembly during neutrophil phagocytosis of microbial pathogens and degranulation, superoxide synthesis by AA/Fe-dependent neutrophil myeloperoxidase (MPO), and conversely, protection from superoxide collateral damage during neutrophil phagolysosome degranulation. SVCT2 transporters on neutrophil cell membranes generate an intracellular AA concentration of 1–2 mmol/L. However, during the oxidative burst, DHA is taken up via GLUT transporters, which is then regenerated to AA to achieve intracellular AA concentrations of 10–20 mmol/L^69^ (**Table 1**).^70–116^

**Table 1.** Studies of Vitamin C in Obesity and Metabolic Syndrome

Model	Study type	Model type	Vitamin C effect	Study
**Oxidative stress**				
Cell model	In vitro	3T3-L1 adipocytes, RAW264.7 macrophages	Vitamin C decreases nitric oxide formation from adipocyte–macrophage interactions and improves WAT inflammation.	Garcia-Diaz et al (2011)^70^
	In vitro	HepG2 cells	Vitamin C protects against ROS in hepatic steatosis.	Yang et al (2018)^71^
	In vitro	HepG2 cells	Vitamin C alleviates hepatocyte cellular stress	Gu et al (2019)^72^
	In vitro	Human plasma	Vitamin C protects against lipid peroxidation at pH 7.4 by regenerating α-tocopherol, mediated by membrane-bound ascorbyl free radical reductase enzyme. This effect is decreased in low pH.	Ojo and Leake (2021)^73^
Human study	RCT, placebo controlled	Healthy (*n* = 80) human adults (20–50 y)	Vitamin C (1 g bd PO/d for 2 wk) inhibits resistin release with antioxidant effects.	Bo et al (2007)^74^
	SRMA RCTs	Human, adults with T2D, 12 RCTs	Vitamin C (1 g/d for 6 wk) reduces fasting and postprandial oxidative stress (MDA).	Balbi et al (2018)^75^
**Fat oxidation**				
Animal model	Prospective nonrandomized	Animal (male guinea pig)	Vitamin C (100 mg/d for 14 d) significantly reduces triglyceride content and increases acid-soluble plasma carnitine.	Otsuka et al (1999)^76^
Human study	RCT	Human, (*n* = 22) hypovitaminotic C adults (plasma AA <28 μmol/L)	Vitamin C repletion (10, 125, and 250 mg vitamin C daily for weeks 1, 2, and 3) significantly reduces serum carnitine level.	Johnston et al (1996)^77^
	RCT	Human, (*n* = 22) overweight adults, 18–38 y	Oral vitamin C repletion (500 mg vitamin C/d) resulted in a 4-fold increase in fat oxidation during submaximal exercise (0.5 ± 0.1 kcal/kg vs 2.0 ± 0.4 kcal/kg, *P *= .01).	Johnston et al (2006)^78^
**Adipokines**				
Cell model	In vitro	Human cells (HeLa, monocytic U937, myeloid leukemia HL-60, breast Ca MCF7, primary endothelial cells)	Vitamin C decreases MCP-1 expression.	Cárcamo et al (2002)^79^
	In vitro	Human, Simpson–Golabi–Behmel syndrome adipocytes	Vitamin C has synergistic effects with other medications on increasing HMW adiponectin production.	Rose et al (2010)^80^
	In vitro	Epididymal VAT adipocytes from male Wistar rats	Vitamin C decreases leptin secretion and ROS production in insulin-incubated cells.	Garcia-Diaz et al (2010)^81^
	In vitro	3T3-L1 adipocytes and RAW264.7 macrophages	Vitamin C inhibits insulin-induced apelin secretion.	Garcia-Diaz et al (2011)^70^
	In vitro	HepG2 cells	Vitamin C reduces NF-κB signaling and proinflammatory cytokine release.	Yang et al (2018)^71^
	In vitro	HepG2 cells	Vitamin C increases the production of HMW adiponectin from adipocytes and hepatocytes.	Gu et al (2019)^72^
	In vitro	Human preadipocytes and adipocytes	Vitamin C supplementation increases HMW adiponectin secretion.	Luo et al (2022)^82^
Human study	RCT	Human, (*n* = 80) adults, 20–50 y	Vitamin C (1 g bd for 2 wk) decreased resistin concentrations (from 4.3 ± 1.5 to 2.9 ± 0.8 ng/mL).	Bo et al (2007)^74^
	Cross-sectional study	Human, (*n* = 219) females, 12 to 17 y	Increased dietary vitamin C estimated intake is associated with increased serum adiponectin level in adolescent females.	del Mar Bibiloni et al (2013)^83^
	RCT	Human, (*n* = 80) male adults with T2D	Supplementation with vitamin C (1000 mg/d for 4 wk) decreased leptin, serum amyloid A protein, and TNF-α concentrations in patients with T2D.	Jamalan et al (2015)^84^
	RCT	Human, (*n* = 84) adults	Vitamin C administration (1000 mg/d for 12 wk) significantly increases plasma total and HMW adiponectin.	He et al (2021)^85^
**Glucose metabolism and insulin resistance**				
Cell model	In vitro	Epididymal VAT adipocytes from male Wistar rats	Vitamin C inhibits glucose uptake in adipose tissue.	Garcia-Diaz et al (2010)^81^
Animal model	Prospective randomized interventional	HFD-fed obese C57BL/6J male mice	Vitamin C supplementation (1% wt/wt vitamin C for 15 wk); ↓plasma glucose concentration by 36%; ↓VAT and pancreatic islet hypertrophy.	Park et al (2018)^86^
	Prospective randomized interventional	Obese C57BL/6J ovariectomized female mice	Vitamin C supplementation (5% wt/wt in diet for 18 wk) significantly reduces insulin resistance and pancreatic steatosis.	Jeon et al (2023)^87^
Human study	RCT	Human, adults with T2D	Vitamin C 500 mg/d + metformin for 1 y ↓ HbA1c (OR, 2.31; 95% CI, 1.87–4.42; *P* < 0.001), ↓ mean FBS ±SD (7.65±2.52 to 5.18±1.66 mmol/L, *P*<.01), ↓ risk of diabetic complications.	Gillani et al (2017)^88^
	SRMA RCTs	Human, (*n* = 1574) adults with T2D, 28 RCTs	Vitamin C supplementation improved: HbA1c (%) (MD, -0.54; -0.90 to -0.17), MCID ≥0.5%; fasting BSL (mmol/L) (MD, -0.74; -1.17 to -0.31), MCID ≥1 mmol/L	Mason et al (2021)^89^
**Cholesterol metabolism and lipid profile**				
Animal model	Prospective interventional	Hypovitaminotic C guinea pigs	Vitamin C deficiency leads to cholesterol supersaturation and formation of gallstones.	Jenkin (1977)^90^
	Prospective interventional	Vitamin C–deficient guinea pigs	Long-term vitamin C deficiency leads to decreased hepatic cholesterol excretion, hypercholesterolemia, and accumulation of cholesterol in hepatocytes.	Gustafsson et al (1997)^91^
	Prospective randomized interventional	Vitamin C–deficient GLO^**−/−**^ mice	Vitamin C deficiency leads to significantly ↑PCSK9, ↓hepatic LDLR, ↑serum LDL, ↓serum HDL, and ↓HDL/LDL ratios, reversed by 2/52 AA supplementation (i.p., 100 mg AA/d/kg body weight).	Wang et al (2020)^92^
Human study	Prospective Interventional	Human, (*n* = 5) healthy volunteers	No effect of experimental AA deficiency (plasma AA = 5.1–8.5 μmol/L) on plasma cholesterol and triglycerides or biliary lipid composition and saturation index of gallbladder bile.	Duane and Hutton (1983)^93^
	Prospective Interventional	Human (*n* = 16), cholecystectomy for cholesterol gallstones	Preoperative vitamin C (500 mg qid PO for 2 wk) decreased cholic acid; increased DCA, UDCA, and LCA; and delayed gallstone nucleation time (7 vs 2 d) in gallbladder bile.	Gustafsson et al (1997)^91^
	Observational	Human (*n* = 2129), 18–65 y	Regular vitamin C supplementation is associated with a significantly decreased risk of gallstones (OR, 0.34; *P* = .01).	Walcher et al (2009)^94^
	SRMA RCTs	Human, adults with T2D and overweight/obesity, 28 studies	Vitamin C supplementation is associated with improvements in: Triglycerides (-0.2 mmol/L, -0.36 to -0.04), MCID ≥1 mmol/L Total cholesterol (-0.27 mmol/L, -0.43 to -0.1), MCID ≥1 mmol/L LDL-C (-0.23 mmol/L, -0.48 to +0.1, NS), MCID ≥ 0.3 mmol/L HDL-C (+0.06 mmol/L, 0.00 to +0.13), MCID ≥ 0.03 mmol/L Plasma MDA (standardized MD, -1.25; 95% CI, -1.88 to -0.62; *P*= .0001)	Mason et al (2021)^89^
**Gut microbiota**				
Animal model	Prospective randomized interventional	HFD-induced obese C57BL/6 male mice	Vitamin C supplementation (30 mg/kg/d for 16 wk) changes gut microbiota composition, improves gut barrier function, and reduces endotoxemia.	Chen et al (2023)^95^
Human study	RCT	Human, adults (*n* = 84) with NAFLD, 18–60 y	Vitamin C (250 mg or 1000 mg/d for 12 wk) improved fecal microbiota composition with an increase in alpha diversity.	He et al (2021)^85^
**NAFLD and NASH**				
Cell model	In vitro	LX-2 HSC cells	Vitamin C enhances collagen synthesis by hepatic stellate cells.	Smith-Cortinez et al (2021)^96^
	In vitro	HepG2 cells	Vitamin C supplementation decreases hepatic lipogenesis and hepatic lipid accumulation.	Wang et al (2022)^97^
Animal model	Prospective interventional	Male N-Mary rats	Vitamin C treatment (30 mg/kg body weight/d) reduces biochemical and histological features of NASH.	Rezazadeh et al (2012)^98^
	Prospective interventional	Male guinea pigs	High-dose vitamin C treatment (2.5 g/kg/d for 3 wk) improves NASH histology.	Park et al (2018)^100^
	Prospective interventional	HFD-fed C57BL/6 male mice	Low-dose (15 mg/kg/d) and medium-dose (30 mg/kg/d) vitamin C reduced NAFLD development in HFD-fed mice, with significantly lowered TBW, perirenal WAT mass, and hepatic steatosis.	Zeng et al (2020)^101^
	Prospective interventional	SMP30 knockout mice	Vitamin C deficiency led to suppressed hepatic lipogenesis. However, hepatocyte cholesterol accumulation was reversed by vitamin C supplementation (1.5 g/L vitamin C containing water for 11 wk).	Lee et al (2021)^102^
	Prospective interventional	Female Hartley guinea pigs	Vitamin C deficiency delays NASH regression in HFD-fed animals changed to an LFD.	Skat-Rørdam et al (2021)^103^
	Prospective interventional	C57BL6 mice wild-type	Oral vitamin C in the drinking water (1.5 g/L) significantly suppressed the development of hepatic steatosis and progression to NASH in wild-type C57BL6 mice fed a high-fat and high-fructose diet (fast-food diet) for 11 wk, as measured by LFTs, hepatocyte triglyceride accumulation, liver inflammation (IL-1β, IL-6, MPO), and liver histology.	Lee et al (2022)^99^
Human study	RCT	Human, adults (*n* = 49) with obesity and biopsy-proven NASH	Vitamin C (1000 mg/d) combined with vitamin E (1000 IU/d) for 6 mo significantly improved NASH fibrosis on histopathology (*P* = .002).	Harrison et al (2003)^104^
	Cross-sectional study (CSS)	Human, (*n* = 101) adults with NAFLD/NASH	No difference in plasma vitamin C concentration between healthy participants and patients with NAFLD/NASH.	Da Silva et al (2014)^105^
	RCT	Human, (*n* = 107) adults with NASH	Vitamin C combined with vitamin E (vitamin E 800 mg/d plus vitamin C 500 mg/d for 12 mo) significantly improves ALT levels, liver fibrosis, and steatosis score.	Barbakadz et al (2019)^106^
	Retrospective CSS	Human, (*n* = 4494) adults	Higher serum vitamin C concentrations have an inverse linear association with liver steatosis, fibrosis, and cirrhosis.	Xie et al (2021)^107^
	RCT	Human, adults (*n* = 84), 18–60 y	Vitamin C (1000 mg/d, 12 wk) significantly decreases AST and ALT levels in pre-existing NAFLD.	He et al (2021)^85^
**Obesity and MetS**				
Human study	RCT	Human, adults (*n* = 41) with obesity	Vitamin C supplementation (1 g/tds for 6 wk) achieved significant weight loss in obese patients (2.53 ± 2.39 kg vs placebo 0.95 ± 1.68 kg, *P* < .012).	Naylor et al (1985)^108^
	RCT, double-blind crossover	Human, adults (*n* = 40) with overweight and NIDDM	Vitamin C supplementation (0.5 g bd for 4 mo) significantly decreased insulin (90 vs 73 pmol/L, *P* < .04), total cholesterol (7.3 vs 5.8 mmol/L, *P* < .03), LDL-C (5.6 vs 4.1 mmol/L, *P* < .05), and triglycerides (2.58 vs 2.08 mmol/L, *P* < .04).	Paolisso et al (1995)^109^
	CSS	Human, (*n* = 580) adult females with overweight and obesity	Plasma vitamin C level is inversely associated with BMI and waist circumference.	García et al (2012)^110^
	Prospective cohort	Human, (*n* = 7569) adults	Borderline association between vitamin C intake and decreased weight gain.	Larsen et al (2014)^111^
	SRMA observational studies	Human, adults with MetS vs control subjects, 28 studies	Dietary vitamin C (RR = 0.93; 95% CI, 0.88–0.97; *P *= .003) and plasma vitamin C (RR = 0.60; 95% CI, 0.49–0.74; *P*< .001) are inversely associated with MetS.	Guo et al (2021)^112^
	Retrospective CSS	Human, (*n* = 8983) adults, with MetS (*n* = 1443)	Higher serum vitamin C concentration is associated with reduced MetS risk.	Pei et al (2023)^113^
	RCT	Human, adults (*n* = 72) with MetS	Micronutrient + vitamin C supplementation (1000 mg/d for 12 wk) achieved small improvements in BMI, fasting glucose, insulin sensitivity, and MetS severity *z* score compared with placebo (*P*< .05).	Vlasiuk et al (2024)^114^
**Hypertension**				
Human study	RCT (double-blind placebo-controlled)	Human, adults (*n* = 84) with T2D	Vitamin C supplementation (250 mg/qid for 45 d) significantly decreased systolic (-7.6 mm Hg, *P* < .0001) and diastolic blood pressure (-4.9 mm Hg, *P* < .0001) in patients with T2D (baseline BMI in AA group 30.6 ± 4.6 kg/m^2^).	Shateri et al (2016)^115^
	RCT (double-blind, placebo-controlled)	Human, adults (*n* = 31) with T2D	Vitamin C supplementation (500 mg/bd for 4 mo) significantly decreased systolic (-7 mm Hg) and diastolic (-5 mm Hg) blood pressure (*P* < 0.05) in patients with T2D (mean BMI, 29.1 ± 3.1 kg/m^2^).	Mason et al (2019)^116^
	SRMA RCTs	Human, adults (*n* = 1574) with T2D, 28 RCTs	Vitamin C supplementation significantly decreased systolic BP (mean difference, -6.27; 95% CI, -9.60 to -2.96 mm Hg; *P* = .0002), and diastolic BP (-3.77; 95% CI, -6.13 to -1.42 mm Hg; *P* = .002) in patients with T2D.	Mason et al (2021)^89^

Abbreviations: AA, ascorbic acid; ALT, alanine aminotransferase; AST, aspartate aminotransferase; bd, twice daily; BMI, body mass index; BP, blood pressure; BSL, blood sugar level; CSS, cross-sectional study; DCA, deoxycholic acid; FBS, fasting blood sugar; GLO, l-gulono-γ-lactone oxidase; HbA1c, glycated hemoglobin; HDL, high-density lipoprotein; HDL-C, high-density-lipoprotein cholesterol; HepG2, human hepatoma; HFD, high-fat diet; HMW, high molecular weight; IL, interleukin; LCA, lithocholic acid; LDL, low-density lipoprotein; LDL-C, low-density-lipoprotein cholesterol; LDLR, low density lipoprotein receptor; LFT, liver function tests; MCID, minimal clinically important difference; MCP-1, monocyte chemoattractant protein-1 (CCL-2); MD, mean difference; MDA, malondialdehyde; MetS, metabolic syndrome; MPO, myeloperoxidase; NAFLD, nonalcoholic fatty liver disease; NASH, nonalcoholic steatohepatitis; NS, not significant; NF-κB, nuclear factor–kappa light chain enhancer of activated B cells; NIDDM, non-insulin–dependent diabetes; OR, odds ratio; PCSK9, proprotein convertase subtilisin/kexin 9; PO, per oral; qid, 4 times per day; RCT, randomized controlled trial; ROS, reactive oxygen species; RR, relative risk; SMP30; senescence marker protein-30; SRMA, systematic review and meta-analysis; T2D, type 2 diabetes, TBW, total body weight; tds, 3 times per day; TNF-α, tumor necrosis factor alpha; UDCA, ursodeoxycholic acid; VAT, visceral adipose tissue; WAT, white adipose tissue.

## FUNCTIONS OF AA OF RELEVANCE TO OBESITY

### Ascorbic Acid and Oxidative Stress

**Figure 2.**
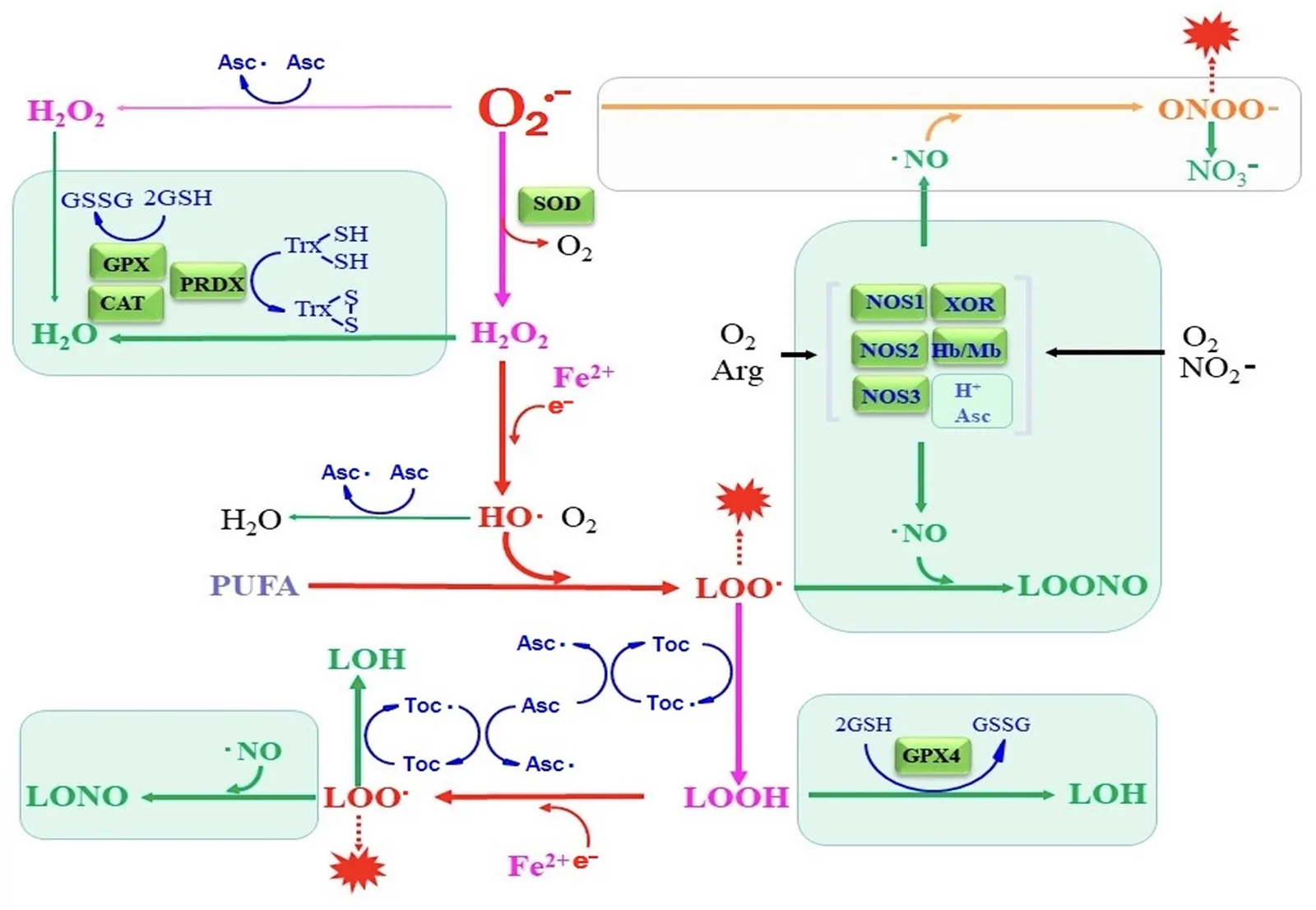
Interaction Between ROS/Lipid Radicals With Antioxidants In Vivo. Detoxification reactions performed by endogenous antioxidative enzymes catalase (CAT), superoxide dismutase (SOD), glutathione peroxidase (GPX), thioredoxin (Trx), and peroxiredoxin (PRDX), and low-molecular-weight antioxidants are represented by green arrows. In addition to antioxidative enzymes, α-tocopherol (Toc), ascorbate (Asc), and nitric oxide (•NO) can terminate free radical chain reactions. One electron oxidation of ascorbate to the stable and nonreactive ascorbyl radical (Asc•) enables regeneration of α-tocopherol from the α-tocopherol radical (Toc•). Hydroxyl radicals (HO•) can be formed from hydrogen peroxide (H_2_O_2_) reacting with ferrous cation (Fe^2+^). Abbreviations: Arg, arginine; Hb, hemoglobin; LOH, hydroxylated lipid; LOOH, lipid hydroperoxide; LOONO, organic peroxynitrite; LOO•, lipid peroxyradical; Mb,myoglobin; NOS, nitric oxide synthase; NO_3_^-^, nitrate; NO_2_^-^, nitrite; ONOO^-^, peroxynitrite; PUFA, polyunsaturated fatty acids; XOR, Xanthine oxidoreductase. Reproduced from Fujii et al.^119^ with permission.

Oxidative stress occurs when the generation or accumulation of reactive oxygen species (ROS) overwhelms the existing antioxidant capacity in a biological system.^117^^,^^118^ Reactive oxygen species are oxygen-containing molecules—for example, superoxide ($O_{2}^{•-}$) hydrogen peroxide (H_2_O_2_), hydroxyl radicals (^•^OH), peroxyl radicals (^•^OOH), and singlet oxygen (^1^O_2_), which can cause oxidative damage to nucleic acids, lipids, and proteins.^38^ Superoxides are generated from the oxidative burst of phagocyte NAD(P)H oxidase (NOX) 2 (NOX-2), from cell membrane-bound nonphagocytic NOX-4, from mitochondria leaking electrons onto molecular oxygen in the electron transport chain, or from xanthine oxidase, cyclo-oxygenase, lipoxygenase, or P450 mono-oxygenase activity.^119^ Superoxides can be dismutated to the nonradical hydrogen peroxide by mitochondrial superoxide dismutase (SOD). Hydrogen peroxides are converted to water and oxygen mainly by catalase, but if they undergo Fenton reactions with ferrous iron can generate highly damaging hydroxyl radicals.^118^ Because superoxides react more quickly with nitric oxide (NO) than with SOD, they readily form peroxynitrites (ONOO^-^) in the presence of NO or uncoupled endothelial NO synthase (eNOS), which form lipid peroxy radicals (LOO^•^), lipid hydroperoxides (LOOH), and secondary products such as the reactive carbonyls malondialdehyde (MDA) and 4-hydroxynonenal (4-HNE).^119^ Endogenous antioxidant enzymes include catalase (CAT), SOD, glutathione peroxidase (GPx), and peroxiredoxin (PRDX), and antioxidant molecules uric acid, bilirubin, glutathione, and thioredoxin (TRX).^119^ Exogenous antioxidants obtained from dietary consumption include vitamins A, C, and E; carotenoids; and flavonoids^120^ (**Figure 2**).

Obesity is associated with an increase in markers of systemic oxidative stress, including MDA (polyunsaturated fatty acid [PUFA] peroxidation), oxidized low-density lipoprotein (ox-LDL; lipid peroxidation), 8-iso-prostaglandin F_2_α (arachidonic acid peroxidation), and 8-hydroxy-deoxyguanosine (DNA oxidation).^121–123^ Excessive superoxide and peroxide generation are related to insulin, glucose, or palmitate stimulation of membrane-bound NOX-4 in white adipose tissue (WAT) in the early stages of obesity.^121–123^ As WAT inflammation progresses, infiltrating macrophages generate ROS via NOX-2 when attempting to phagocytose saturated fatty acids and cholesterol crystals in WAT hypertrophy and in crown-like structures (CLS) in NASH. Excessive nutrient flux also leads to dysfunctional mitochondria and mitochondrial ROS release.^121–123^ Peroxides derived from NOX-4 can stimulate further mitochondrial ROS generation, and peroxides from phagocyte NOX-2 can lead to a positive feedback loop with nonphagocytic NOX-4, thus creating further oxidative products, termed ROS-induced ROS release.^121–123^

A pathological increase in ROS levels in obesity can lead to DNA/RNA damage, lipid peroxidation, stress-induced cellular senescence, metabolic inflammation (metaflammation), endothelial dysfunction, and insulin resistance.^124^^,^^125^ Patients with MetS have increased ox-LDL and DNA damage response (DDR), decreased plasma ascorbate, and impaired antioxidant protection against ROS.^126^ The hydroxyl groups in AA are donors of electrons or protons, which creates a strong reducing capability of AA against oxygen free radicals, but not hydrogen peroxide. Ascorbate scavenges superoxide radical and singlet oxygen more than 100 times faster than glutathione.^117^ During its oxidation, ascorbate loses a first electron to form ascorbyl radical (SDA^•^/Asc^•-^) and then a second electron to form DHA.^127^ Further irreversible oxidation of DHA results in oxalic acid, which can also be a pro-oxidant.^128^

**Figure 3.**
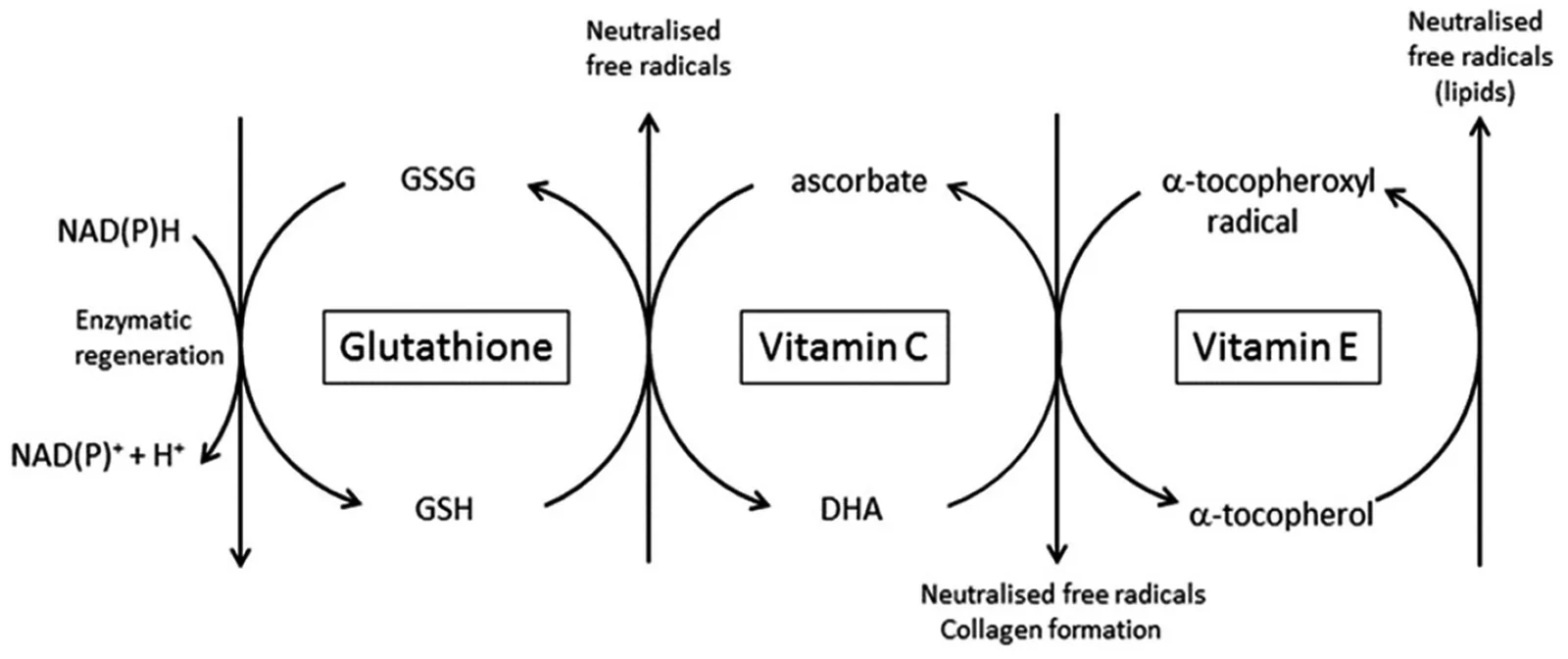
Interdependence of Vitamins E (α-Tocopherol) and C, Glutathione, and NAD(P)H in the Scavenging of Free Radicals and Regeneration of the Reduced Antioxidants Is Shown. Regeneration of α-tocopherol from α-tocopheroxyl radical by ascorbate and protection against lipid peroxidation occurs at the lipid/aqueous phase interface. Abbreviations: DHA, dehydroascorbate; GSH, reduced glutathione; GSSG, oxidized glutathione; NAD(P)H, nicotinamide adenine dinucleotide (phosphate), reduced form. Reproduced from Pullar et al.^129^ with permission.

Ascorbate not only scavenges hydroxyl, peroxyl, and superoxide radicals, but also regenerates other antioxidants including urate, α-tocopherol (vitamin E), β-carotene, and glutathione.^129^^,^^130^ Ascorbate regenerates oxidized glutathione (GSSG) to reduced glutathione (GSH) in the aqueous phase. Although ascorbate is a water-soluble antioxidant, it also regenerates α-tocopherol by reducing its oxidation product, the α-tocopheroxyl radical, thereby protecting against lipid peroxidation (**Figure 3**).^129^ This effect can be mediated by membrane-bound ascorbyl free radical reductase enzyme, which regenerates ascorbate at the liquid/lipid interface of cell membranes.^73^ Supplementation with AA and vitamin E can improve endogenous enzymatic antioxidant (catalase, glutathione peroxidase, and GSH) levels in erythrocytes and plasma by scavenging of free radicals, regeneration of enzymatic antioxidants, and lowering lipid peroxidation (MDA formation) and ox-LDL formation.^131^

However, multiple large clinical trials of antioxidant supplementation failed to show benefit in the prevention of insulin resistance and T2D.^132^ It has been suggested that, because physiological amounts of peroxide are necessary for redox signaling, endoplasmic reticulum (ER) oxidative protein folding, and insulin sensitivity, suboptimal antioxidant dosing may inhibit ER hormesis and explain some of the disappointing results from clinical trials compared with preclinical trials.^132^ For example, peroxide is both generated and consumed by the ER protein disulfide isomerase (PDI)/ER oxidoreductin-1 (ERO1)/PRDX4 pathway, which utilizes molecular oxygen and DHA via GLUT10 transport in the folding of proteins. This redox-dependent system can be disrupted by altering the concentrations of ER ascorbate, DHA, GSH, GPX7, GPX8, or PRDX4.^133^^,^^134^

Cellular protection from excessive ROS can be regulated by nuclear factor erythroid-derived 2-like 2 (NFE2L2), SOD1, and quinine oxidoreductase-1 (NQO1).^135^^,^^136^ Ascorbic acid induced NFE2L2 signaling and inhibited ER stress marker genes in oleic acid (OA)–treated liver hepatoma (HepG2) cells, and provided in vitro cellular protection against ROS in OA-induced HepG2 steatosis.^71^ In another in vitro study, HepG2 cells were treated with tumor necrosis factor alpha (TNF-α) to mimic hepatocyte stress during nonalcoholic fatty liver disease (NAFLD)/NASH.^72^ Administration of ascorbate inhibited the expression and decreased the protein level of hypoxia inducible factor 1 alpha (HIF-1α), monocyte chemoattractant protein 1 (MCP-1), and glucose-regulated protein (GRP), and increased the expression of fibroblast growth factor 21 (FGF21) and its receptor, FGFR2, as well as adiponectin in HepG2 cells. Ascorbate provision effectively alleviated hepatocyte cellular stresses, including hypoxia, inflammation, and ER stress.^72^

Obesity is associated with WAT macrophage M1 polarization, metaflammation, and increased systemic oxidative stress.^70^ In co-cultures of 3T3-L1 adipocytes and RAW264.7 macrophages, AA decreased NO formation from adipocyte-macrophage interactions.^70^ Ascorbic acid may inhibit inducible NO synthase (iNOS) mRNA and iNOS expression induced by hyperinsulinemia and hyperglycemia. Ascorbic acid also decreased MCP-1 expression in co-cultures of adipocytes and macrophages, which may diminish macrophage chemotaxis and WAT inflammation.^70^

In a trial of healthy human adults, AA (1 g twice daily per oral [PO]) for 2 weeks inhibited resistin release and contributed to an antioxidant effect, as demonstrated by decreased serum nitrotyrosine and elevated erythrocyte concentrations of the reduced form of glutathione (GSH).^74^ Ascorbic acid has a dual action of reducing oxidative stress and subsequent inflammation. Ascorbate acts as a hydrophilic scavenger of intracellular oxygen free radicals, thereby generating DHA. Dehydroascorbate, but not ascorbate, can directly inhibit IκB kinase α/β (IKKα/β) and block activation of the nuclear factor–kappa light chain enhancer of activated B cells (NF-κB) inflammasome.^137^

### Ascorbic Acid–Dependent Oxygenase Enzyme Function

Ascorbic acid is a cofactor for transition-metal dependent mixed-function oxygenase, mono-oxygenase, and dioxygenase enzymes, as it maintains the central copper or iron atoms of these enzymes in a reduced state after each catalytic cycle.^94^^,^^117^ Ascorbic acid reduces ferric cation (Fe^3+^) to ferrous cation (Fe^2+^) in dioxygenase enzymes. This enables 2-oxoglutarate and molecular oxygen to oxidize the substrate, resulting in a hydroxylated product together with the formation of succinate and carbon dioxide. The net result is a (highly controlled) pro-oxidant activity of these enzyme systems on specific substrates due to the reducing capacity of AA (**Figure 5**).^117^ The oxygenases require a high intracellular concentration of AA for their optimal function since the *K*_m_ for AA ranges from 140 to 300 µmol/L. The non-heme iron-dependent and 2-oxoglutarate–dependent dioxygenases comprise over 80 enzymes, including those required for the following:

1. Collagen and high-molecular-weight (HMW) adiponectin synthesis: procollagen proline 2-oxoglutarate 4-dioxygenase (procollagen prolyl-4-hydroxylase [CP4H] and procollagen prolyl-3-hydroxylase [CP3H]), procollagen lysine 2-oxoglutarate 5-dioxygenases (PLODs; lysyl hydroxylases)
2. Carnitine synthesis: trimethyllysine hydroxylase epsilon (TMLHE), gamma-butyrobetaine dioxygenase (BBOX)
3. HIF-1α regulation by hydroxylation: prolyl hydroxylase domain-containing proteins (PHDs), factor inhibiting HIF (FIH; asparaginyl hydroxylase enzyme)
4. Epigenetic regulation of DNA translation by histone deacetylation or DNA demethylation: Jumonji-C domain histone demethylase (JHDMs), lysine demethylases (KDMs), alkylated DNA repair protein AlkB homologs (AlkBHs), fat-mass and obesity-associated protein (FTO), Myc-induced nuclear antigen 53 (MINA53), nucleolar protein 66 (NO66), and ten-eleven translocation methylcytosine dioxygenase (TET). (**Figure 5**)

The copper-dependent mono-oxygenases rely on AA to reduce the central copper atom, from the stable cupric ion (Cu^2+^) to the unstable and highly reactive cuprous ion (Cu^+^), and are required for catecholamine synthesis by dopamine-β-hydroxylase (noradrenaline) and hormone and neurotransmitter synthesis by α-amidation of peptides via peptidyl-glycine α-amidating mono-oxygenase (PAM)—for example, calcitonin, oxytocin, thyrotropin-releasing hormone (TRH), vasopressin, growth hormone, substance P, neuropeptide Y, glucagon-like peptide 1 (GLP-1), gastrin, and cholecystokinin production.^35^

Ascorbic acid is also required to regenerate tetrahydrobiopterin (BH4) for the activity of non-heme (FeII)–dependent aromatic amino acid mono-oxygenases, including the following: (1) tyrosine hydroxylase in the conversion of the amino acid l-tyrosine to the dopamine precursor l-3,4-dihydroxyphenylalanine (l-DOPA) and (2) tryptophan hydroxylase in the conversion of l-tryptophan to serotonin (5-hydroxytryptamine).^120^ Enzyme cofactor functions of relevance to obesity will be discussed in more detail below.

### Ascorbic Acid and l-Carnitine Synthesis

Classical symptoms of AA deficiency include lethargy, muscle weakness and tenderness, aversion to exercise, and easy fatigue, thought to be, in part, related to l-carnitine deficiency.^34^^,^^138^ It has also been suggested that the lassitude, decreased physical activity, malaise, and mood swings of early AA deficiency are related to inadequate catecholamine/neurotransmitter production, as AA is a co-substrate for tyrosine hydroxylase, which converts tyrosine to l-DOPA, and a co-factor for dopamine-β-monooxygenase, which converts dopamine to noradrenaline.^34^^,^^138^

l-Carnitine is important for the transport and mitochondrial oxidation of fatty acids.^139^ It is a quaternary ammonium compound (β-hydroxy-γ-N-trimethylaminobutyric acid) derived from the amino acids lysine and methionine, and is obtained from red meat or endogenously manufactured in humans in the liver.^139^ It is then distributed via the bloodstream to cardiac and skeletal muscle. The recommended daily allowance of l-carnitine is 500–2000 mg/day, with the bioavailability of l-carnitine derived from whole foods being high (red meat eaters, 54%–72%). However, supplemental oral l-carnitine has poor intestinal bioavailability (5%–25%). Healthy individuals adapted to low-carnitine diets, including those who avoid meat and dairy consumption, can usually increase intestinal absorption of dietary l-carnitine to 66%–86%, maintain high renal tubular l-carnitine reabsorption (98%–99%), or synthesize enough l-carnitine to avoid deficiency.^139^  l-Carnitine is essential for the transportation of long-chain fatty acyl coenzyme As (CoAs) across the mitochondrial membrane by carnitine palmitoyltransferase 1/2 (CPT-1, CPT-2) and the carrier carnitine/acylcarnitine translocase (CACT) for subsequent mitochondrial fatty acid β-oxidation.^139^ There are 2 peroxisomal carnitine acyltransferases, carnitine octanoyltransferase (COT) and carnitine acetyltransferase (CAT), which transport very-long-chain fatty acids (VLCFAs), dicarboxylic acids (DCAs), and branched-chain fatty acids (BCFAs) into the peroxisome for β-oxidation.^140^ Impaired mitochondrial and peroxisomal fatty acid β-oxidation and metabolic dysfunction secondary to carnitine insufficiency are associated with obesity, cardiovascular disease, hypertension, and diabetes.^141^^,^^142^ Ascorbic acid is a cofactor for ε-N-trimethyl-l-lysine hydroxylase and γ-butyrobetaine hydroxylase, which are essential enzymes in l-carnitine biosynthesis.^143^

By increasing l-carnitine synthesis, AA may stimulate fatty acid oxidation and reduce triglyceride accumulation.^77^^,^^144^ Repletion with high-dose AA (100 mg/d) in guinea pigs previously fed an AA-deficient diet led to increased muscle acid-soluble carnitine concentration and decreased plasma triglyceride content.^76^ The relationship between plasma carnitine, fatty acid oxidation (FAO), and marginal AA status was also studied in sedentary young human adults aged 18 to 38 years in an 8-week double-blind, placebo-controlled AA depletion-repletion trial.^78^ The study showed that AA-depleted (mean plasma AA ± SE = 9.7 ± 1.0 μmol/L) young adults (mean BMI = 25 ± 1.3 kg/m^2^) oxidized 4 times less fat and were fatigued during 60 minutes of treadmill walking, compared with participants with adequate AA concentrations (respective fat oxidation, 0.5 ± 0.1 kcal/kg vs 2.0 ± 0.4 kcal/kg; *P *= .01). Fat oxidation by participants during submaximal exercise (50% of their VO_2_max) was inversely related to fatigue (*r *= -0.6, *P *= .009). Ascorbic acid–depleted individuals had higher levels of protein oxidation compared with repleted individuals (0.9 ± 0.3 and 0.4 ± 0.1 kcal/kg, respectively; *P *= .06), which suggested that oral AA repletion (500 mg/d) resulted in improved fat oxidation and protein sparing during aerobic exercise.^78^ Plasma vitamin C concentrations were only weakly correlated with plasma carnitine concentrations, and muscle carnitine was not measured. The authors suggested that AA deficiency could contribute to obesity due to exercise intolerance, easy fatigue, and reduced mitochondrial fat oxidation.^78^

Ascorbic acid also increases serotonin and noradrenaline levels, which can affect mood, motivation, exercise performance, level of exhaustion, and subjective perception of effort.^34^^,^^138^^,^^145^^,^^146^ However, whether AA deficiency directly leads to l-carnitine depletion is controversial. A study in AA-insufficient guinea pigs suggested that increased urinary loss of l-carnitine, instead of decreased l-carnitine biosynthesis, significantly contributed to the observed carnitine depletion.^147^ A study using SMP30 and GLO KO mouse models showed no significant difference in tissue or serum l-carnitine concentrations between AA-supplemented and AA-deprived mice, suggesting that AA is not essential for l-carnitine biosynthesis. This is due to GSH potentially replacing AA to maintain adequate l-carnitine synthesis.^148^ There is no consistent relationship between AA and plasma l-carnitine concentrations in humans. However, it is thought that the reduction in tissue carnitine concentrations may contribute to the early signs of AA deficiency, including fatigue and exercise aversion^78^ (**Table 1**).

## ASCORBIC ACID, THE EPIGENOME, AND DNA METHYLATION

The modern obesity epidemic appears to have occurred too rapidly to be explained by classic Darwinian evolutionary theory. However, epigenetic control of DNA expression and intergenerational epigenomic inheritance of both maternal and paternal ancestral environmental and dietary experiences can influence eating behavior, energy consumption, thermogenesis, and phenotype.^28–32^ The DNA epigenome is involved in obesity and aging, including promoter cytosine and guanine dinucleotide (CpG) methylation, histone acetylation and methylation, and noncoding RNA (lncRNA, miRNA). DNA methylation is related to oxidative stress upregulation of DNA methyltransferases (DNMTs). Together with dietary methyl donors (methionine), DNMTs catalyze and promote DNA methylation. Epigenetic control of gene expression is also related to excessive acetyl-CoA generation in obesity, which contributes to DNA histone acetylation and diminished sirtuin 1 (SIRT-1).^28–32^^,^^149^

For example, global cytosine and guanine dinucleotide (CpG) island hypomethylation of adipogenic genes was found in post-obese women after bariatric surgery, compared with never-obese women.^150^ Methylation of adipocyte leptin (*LEP*), fat mass and obesity-associated (*FTO*), insulin receptor substrate 1 (*IRS1*), and *HIF3A* genes is associated with obesity-induced WAT hypertrophy and T2D. Differentially methylated CpGs in the promoter region genes of adiponectin and FGF21 are also associated with WAT dysfunction and MetS.^151^

DNA and histone methylation is regulated by the AA-dependent TET protein family (TET1, TET2) and JHDM, respectively. Both TET1 and TET2 can be recruited by peroxisome proliferator-activated receptor (PPAR) gamma (PPAR-γ) and induce CpG demethylation to suppress adipogenesis.^151^ Ascorbic acid acts as a cofactor for TET and JHDM dioxygenase enzyme function by enabling the reduction of enzymatic ferric cation (Fe^3+^) to ferrous cation (Fe^2+^) (**Figure 5**). Ascorbic acid can effectively suppress lipid production, reverse adipocyte hypertrophy, and reduce hepatic steatosis in high-fat-diet (HFD)–fed Tet1 haplo-insufficient mice by activation of TET1, normalization of DNA methylation, and subsequent increase in hormone-sensitive lipase and PPAR-α gene expression.^152^^,^^153^ However, the relationship between TET and obesity is complex, as complete suppression of adipocyte TET leads to increased β_3_-adrenergic receptor (β3-AR) expression and activity, triglyceride lipolysis, and thermogenesis in beige adipose tissue with associated systemic energy expenditure.^154^^,^^155^ This improved cold intolerance and protected against body-weight gain and metabolic disorders under HFD feeding in male C57BL/6J TET KO mice.^154^^,^^155^ Extrapolating this finding to humans may be problematic as lipolysis, thermoregulation, and adipocyte AR receptor regulation substantially differ between mice and humans.

## ASCORBIC ACID AND ADIPOKINES

White adipose tissue is a functional endocrine organ that releases various adipokines to regulate metabolic physiology.^156^ Adipokines play an important role in obesity, insulin resistance, and inflammation. Adipokines can be either proinflammatory (leptin, interleukin [IL]-6, TNF-α, retinol binding protein 4 [RBP4], resistin, chemerin, angiopoietin-like 2 [ANGPTL2], C-C motif ligand 2 [CCL2], CCL5) or anti-inflammatory (adiponectin, secreted frizzled-related protein 5 [SFRP5], Omentin-1, Apelin, C1q/TNF-related protein [CTRP] family, FGF21).^156^ In obesity, adipocyte hypertrophy, ROS generation, lipotoxicity, and hypoxia lead to a stress response with altered adipokine secretion favoring proinflammatory adipokines. Leptin affects lipid metabolism in multiple systems, regulates innate immunometabolism, and contributes to the inflammatory state in obesity. Conversely, adiponectin has an anti-inflammatory effect by inhibiting proinflammatory cytokines (IL-6 and TNF-α) and promoting secretion of anti-inflammatory cytokines (IL-10).^157^

Adipocyte-derived adiponectin also improves the efflux of cholesterol from lipid-laden macrophages (foam cells) into high-density-lipoprotein (HDL) cholesterol, thereby preventing atherosclerosis and WAT CLS formation.^158^ This is achieved by adiponectin activating liver X receptor α and PPAR-γ, which leads to increased expression of the ATP-binding cassette transporter (ABCA1). ABCA1 is the cholesterol efflux regulatory protein (CERP) in humans, which induces HDL assembly through reverse cholesterol transport. In humans, serum adiponectin concentrations are highest in lean individuals with functional adipocytes, and reduced in obesity by AA deficiency (see mechanism below), adipocyte hypoxia, ER and oxidative stress, and inflammatory cytokines.^82^^,^^158^ Serum adiponectin concentrations are inversely proportional to BMI and waist–hip ratio.^158^ Obesity is also characterized by decreased adiponectin sensitivity in skeletal muscle and liver, which creates a feed-forward effect of progressive insulin resistance, as hyperinsulinemia represses adiponectin receptor 1 and 2 (AdipoR1/R2) mRNA expression via activation of phosphatidylinositol 3-kinase (PI3K) and inactivation of Forkhead box protein O1 (Foxo1).^159^

### Ascorbic Acid, Adiponectin, and Hypoxia

Adiponectin shares homologous domains with collagen X, VIII, and with complement protein C1q.^82^^,^^160^ Ascorbic acid is a cofactor for the prolyl-hydroxylases and lysyl-hydroxylases that are essential for the hydroxylation of low-molecular-weight adiponectin and formation of HMW adiponectin multimers through interactions between its collagen-like domains.^82^ Failure of adiponectin multimerization causes retention of folded adiponectin protein in adipocyte ER, resulting in ER stress and low serum adiponectin concentrations.^82^

In human adipocytes, treatment with AA (100 μmol/L) resulted in a 5-fold increase in HMW adiponectin secretion in vitro within 24 hours.^82^ Ascorbic acid increases the production of HMW adiponectin from adipocytes and hepatocytes, and ameliorates hepatocyte stress response to hypoxia, TNF-α–related inflammation and ER stress by activation of the FGF21/FGFR2/adiponectin pathway, and inhibition of HIF-1α.^72^ A positive association between oral AA intake and serum adiponectin has been reported in adolescent females aged 12–17 years.^83^ Ascorbic acid may also have a synergistic effect on increasing HMW adiponectin production when combined with other medications—for example, thiazolidinediones, statins, or angiotensin II type 1 receptor (AT1R) blockers (losartan, irbesartan).^80^ The elevation of plasma adiponectin by AA administration was independent of the AA dosage in a clinical randomized controlled trial (RCT) in humans with NAFLD using oral doses of 200 mg, 1000 mg, and 2000 mg per day.^85^

White adipose tissue hypoxia is a feature of obesity due to increased adipocyte oxygen consumption, inadequate WAT capillary density, impaired neo-vascularization, local angiotensin release, WAT fibrosis, OSA, and adipocyte hypertrophy exceeding the O_2_ diffusion limit (>100 µm).^17^ Oxygen and AA are both required for the normal synthesis, oxidative protein folding, and release of adiponectin from adipocyte ER.^82^ During normoxia, AA is essential for the hydroxylation of the HIF-1α protein by HIF prolyl-hydroxylases (PHDs) and asparagine hydroxylases (FIH). Hydroxylated HIF-1α can be ubiquitinated by the von Hippel-Lindau (VHL) protein and then rapidly destroyed in the proteosome^161^ (**Figure 5**).

HIF-1α protein contributes to the chronic inflammation and insulin resistance of obesity, and is stabilized by hypoxia, OSA, adipocyte hypertrophy, leptin, insulin, ROS, IL-1β, adipogenesis, iron deficiency, or AA deficiency.^162^^,^^163^ High-fat-diet feeding in mice resulted in saturated fatty acid stimulation of the inner mitochondrial membrane protein adenine nucleotide translocase 2 (ANT2), leading to uncoupled mitochondrial respiration, increased oxygen consumption, and a state of relative adipocyte cellular hypoxia. This resulted in induction of HIF-1α and proinflammatory signaling as early as 1 and 3 days, respectively, after initiation of HFD feeding, and before the development of obesity.^82^^,^^164^ HIF-1α inhibits the expression of the adipocyte adiponectin gene (*ADIPOQ*) through a SOCS3–signal transducer and activator of transcription 3 (SOCS3-STAT3) pathway. Human 3T3-L1 adipocytes exposed in vitro to excessive palmitate and TNF-α produced much greater levels of HIF-1α and its downstream HRE transcription targets vascular endothelial growth factor A (VEGFA) and GLUT1, which could be completely reversed by AA administration. Ascorbic acid also significantly reduced markers of inflammation (IL-6, MCP1, TNF-α) and ER stress (spliced X-box-binding protein-1 [sXBP1], CCAAT-enhancer-binding protein homologous protein [CHOP] and Glucose-Regulated Protein 78 [GRP78]). The reduction of ADIPOQ mRNA expression in hypertrophic human adipocytes could not be reversed by AA administration; however, the ER production of HMW adiponectin was increased by AA under all conditions. This effect was replicated in in vivo murine models.^82^^,^^164^

### Ascorbic Acid and Leptin

Leptin is a 16-kDa protein hormone secreted by adipocytes, and circulating leptin concentrations are positively correlated with fat mass.^81^^,^^84^^,^^165–167^ Leptin is involved in regulation of food intake, energy expenditure, and appetite via leptin receptors and H_2_O_2_ signaling in anorexigenic pro-opiomelanocortin/cocaine-amphetamine related transcript (POMC/CART)–co-expressing neurons and orexigenic agouti-related peptide/neuropeptide Y (AgRP/NPY) neurons in the hypothalamic arcuate nucleus (ARC).^81^^,^^84^^,^^165–167^ Leptin resistance is specifically related to ER stress, the ER unfolded protein response (UPR), and inhibition of leptin-induced STAT3 phosphorylation, involving hypothalamic neurons, hepatocytes, and adipocytes. ER stress occurs very early in lean animals fed an HFD, even before the development of obesity. Improving the folding capacity of the ER by supplementation with the chaperones tauro-ursodeoxycholic acid or 4-phenyl butyric acid improves ER stress and leptin signalling.^166^ It has been reported that a higher level of DHA can lead to leptin resistance via upregulation of ER-stress related genes (GRP78, CHOP, spliced XBP1), and inhibition of leptin-induced STAT3 phosphorylation. Increased oxidation of ascorbate to DHA is commonly found in diabetes.^167^ Supplementation with AA can decrease leptin, serum amyloid A (SAA) protein, and TNF-α concentrations in diabetic patients with leptin resistance and metaflammation.^84^ A concentration-dependent decrease in leptin secretion from insulin-incubated cells in response to AA has been reported.^81^

### Ascorbic Acid and Other Adipokines

Ascorbic acid can also inhibit insulin-induced apelin secretion.^70^ This decreases the WAT inflammatory status by reducing the interaction between adipocytes and macrophages.^70^ Vitamin C supplementation significantly decreases resistin concentrations in human adults, with each unit increase of serum vitamin C corresponding to 0.1 unit of resistin concentration decrease on linear regression analysis.^74^ Increased secretion of MCP-1 by adipose tissue in hypertrophic obesity promotes inflammatory adipokine secretion and macrophage chemotaxis, leading to WAT macrophage infiltration and inflammation. Ascorbic acid can decrease MCP-1 expression by inhibiting TNF-α–induced NF-κB activation and thereby modulate interactions between adipocytes and macrophages.^70^^,^^79^ Ascorbic acid effectively reduced NF-κB signaling and proinflammatory cytokine (IL-1α, IL-6, IL-8, and TNF-α) release in HepG2 cells exposed to oleic acid in an in vitro model of NAFLD, although astaxanthin (ATX) was more effective than AA or N-acetyl cysteine (NAC) in inhibiting hepatocyte cell death, lipotoxicity, and inflammation.^71^

## ASCORBIC ACID AND GLUCOSE METABOLISM

Insulin resistance, glucose metabolism dysfunction, ROS generation, lipoprotein oxidation, M1-like macrophage polarization, cytokine release, and hepatocyte, pancreatic islet β-cell, and adipocyte oxidative stress arise from inflammatory Western diets and sedentary lifestyles.^28^^,^^168^ Exercise can increase glucose transport in contracting muscles by 50-fold in humans, which is important in maintaining plasma glucose levels and insulin sensitivity.^169^ Chronic oxidative stress inhibits insulin receptor substrate (IRS) signaling and insulin-induced GLUT4 translocation in adipocytes, leading to a decrease in glucose uptake, insulin resistance, and plasma hyperglycemia.^168^ Increased insulin production contributes to pancreatic islet β-cell ER stress; dysfunctional folding of proteins, such as insulin, adiponectin, and collagen in the ER lumen; and the ER UPR. While mild ER stress enables increased production of insulin, unresolved UPR during chronic glycotoxicity and lipotoxicity (palmitate exposure) activates UPR death receptor pathways, resulting in pancreatic islet β-cell apoptosis and eventual pancreatic endocrine failure and T2D.^170^^,^^171^

Ascorbic acid is a stimulatory cofactor for mitochondrial glycerol-3-phosphate dehydrogenase (GPDH), based on studies of scorbutic guinea pigs.^172^ Ascorbic acid deficiency causes loss of activity of Flavin adenine dinucleotide (FAD)-dependent mitochondrial GPDH, and therefore impairs the glycerol-3-phosphate shuttle, required for re-oxidation of glycolysis-derived NADH, mitochondrial oxidative phosphorylation, ATP-dependent insulin secretion, and glucose tolerance.^172^ Excessive flux of long-chain fatty acids through the TCA cycle during nutrient overload, increased plasma non-esterified fatty acid (NEFA) levels, and obesity leads to premature mitochondrial transfer of electrons onto molecular oxygen and mitochondrial generation of superoxides.^173^ These superoxides are dismutated to peroxides by mitochondrial MnSOD and can be distributed to the ER and cytoplasm via aquaporin 11. Elevated intracellular peroxides can oxidize thiols on the dedicated peroxide binding site in the glyceraldehyde-3-phosphate dehydrogenase (GAPDH) enzyme, which compromises its glycolytic activity in both mouse and human cells. This leads to accumulation of upper glycolytic intermediates in the cytoplasm, including glucose-6-P, fructose-6-P, and glyceraldehyde-3-P.^173^

Ascorbic acid in the mitochondrial matrix is important for supporting SOD2, GSH, Trx, Prx, and catalase function in ROS scavenging.^174^ Hyperglycemia inhibits GLUT1 transporters and DHA uptake, contributing to mitochondrial AA and GSH depletion, and mitochondrial oxidative stress.^8^^,^^172^ Mitochondrial oxidation of excess NADH to NAD^+^ and peroxide stress during hyperglycemia, particularly in hepatocytes and pancreatic islet β-cells, contributes to cytoplasmic GAPDH inhibition.^173^^,^^175^ GAPDH acts as a redox sensitive switch to divert glucose flux away from pyruvate generation and the TCA cycle toward the polyol, hexosamine, protein kinase C, and toxic aldehyde (methylglyoxal)/advanced glycation end-products (AGE) pathways.^173^^,^^175^ Diverting glucose-6-P into the oxidative arm of the pentose phosphate pathway (ox-PPP) can maintain reductive capacity by the increased production of NAD(P)H. NAD(P)H is a cofactor for the glutathione reductase/glutathione peroxidase/GSH, Trx reductase (TrxR)/Trx/peroxiredoxin, and catalase systems, which allow cells to scavenge elevated peroxide levels and tolerate and survive oxidative stress^121–123^^,^^173^^,^^174^ (**Figure 3**). However, many of the metabolic complications of human obesity, including intracellular sorbitol and fructose accumulation, diacylglycerol (DAG) synthesis*,* hepatic de novo lipogenesis, T2D nephropathy/peripheral neuropathy, microvascular disease, aberrant protein glycosylation (eg, glycated hemoglobin [HbA1c]), advanced glycation end-product (AGE)–Receptor for AGE (RAGE) interactions, metaflammation, formation of neutrophil extracellular traps (NETosis), accelerated atherosclerosis, NAD(P)H oxidase activation, and further oxidative and carbonyl stress are related to the GAPDH redox switch effect and hyperglycemia.^175–178^ During hyperglycemia when hexokinase becomes saturated, up to 30% of glucose is directed to the polyol pathway, as compared with only 3% flux of glucose under normal redox potential and euglycemic conditions.^179^ In the polyol pathway, aldose reductase converts glucose to sorbitol which consumes NAD(P)H, and sorbitol dehydrogenase oxidizes sorbitol to fructose, which depletes NAD^+^ and forms excessive NADH.^176–178^ Sorbitol dehydrogenase is deficient in the lens and absent in the retina, renal glomeruli, and Schwann cells, and thus sorbitol accumulation in diabetes contributes to the development of cataracts, retinopathy, nephropathy, and peripheral neuropathy.^180^ Downstream effects of excessive fructose metabolism include acetylation of CPT-1*α,* inhibition of mitochondrial FAO, depletion of ATP, and a state of cellular “pseudohypoxia,” leading to abnormal feeding behavior and fat deposition^175–178^ (**Figure 6**).

### Ascorbic Acid, Insulin, and Glycotoxicity—Cell and Animal Models

Ascorbic acid can affect glucose metabolism by competing with glucose for transmembrane transport. An in vitro study showed dependent inhibition of glucose uptake in rat epididymal primary adipocytes, due to competitive inhibition of GLUT1 and GLUT3 transporters by DHA.^81^ Conversely, in conditions of hyperglycemia, DHA transport into cells by GLUT1 is inhibited by substrate competition with glucose in vitro and in vivo, but also by GLUT1 glycosylation.^47^^,^^181^ Erythrocytes are exposed to endogenous peroxide production from oxyhemoglobin auto-oxidation by Fenton reactions and also exogenous peroxides, and failure of GLUT1 transport of DHA during hyperglycemia causes erythrocyte oxidative stress and loss of membrane β-spectrin.^47^ It has been suggested that such impaired DHA transport and associated membrane cytoskeletal changes contribute to the impaired erythrocyte deformability, microangiopathy, and microvascular hypoxia found in poorly controlled diabetes (HbA1c >8%),^47^^,^^182^ but this remains to be confirmed.^183^ Increased glycosylation of hemoglobin (HbA1c) and 2,3-diphosphoglycerate erythrocyte binding sites increases hemoglobin affinity for oxygen, which also impairs tissue oxygen delivery in T2D.^47^^,^^181^^,^^184^ Ascorbic acid (5% wt/wt in diet for 18 weeks) effectively reduced insulin resistance and pancreatic steatosis by suppressing adipose tissue inflammation (WAT CLS and CD68-positive macrophages), and visceral adipocyte hypertrophy in ovariectomized HFD obese C57BL/6J mice, in a murine model of postmenopausal human obesity.^87^ A significant reduction in pancreatic islet hypertrophy was reported in HFD-induced obese C57BL/6J mice administered AA, together with 36% lower plasma glucose concentrations, and a 36% and 15% respective decrease in expression of lipogenic stearoyl CoA desaturase 1 (SCD1) and sterol regulatory element-binding protein 1c (SREBP-1c) mRNA.^86^

### Ascorbic Acid Intake and T2D in Human Studies

Reducing the levels of oxidative stress and improving serum AA by plant-based diets or oral supplementation may be beneficial in humans with obesity, hyperinsulinemia, dyslipidemia, and T2D. However, the magnitude of the effect of oral AA supplementation on insulin sensitivity, fasting blood glucose, HDL cholesterol, lipid oxidation, and hemoglobin glycosylation (HbA1c) in clinical trials and meta-analyses can be clinically relevant, marginal, or ineffective^75^^,^^88^^,^^89^^,^^181^^,^^185–188^ (**Table 1**). For example, Mason et al^89^ in their systematic review and meta-analysis (SRMA) of 28 RCTs involving AA supplementation in adult humans with T2D found a mean difference of -0.54% in HbA1c with a mean clinically important difference (MCID) of 0.5% or greater HbA1c, decreased fasting blood sugar level (mmol/L; MD, -0.74; -1.17 to -0.31; MCID ≥1 mmol/L), and improved HDL cholesterol (mmol/L; MD, +0.06; 0.00 to +0.13; MCID ≥0.03 mmol/L) and MDA (standardized mean difference, -1.25; 95% CI, -1.88 to -0.62; *P *= .0001), but with no significant effects on fasting insulin, Homeostatic Model Assessment of Insulin Resistance (HOMA-IR), or insulin clamp sensitivity. Contributing to this uncertainty are study heterogeneity, protocol variation, low cohort numbers (<100 patients) in individual studies, short duration of AA administration (<12 weeks), oral AA doses less than 1000 mg/day, and lack of measurement of baseline and prospective serum AA concentrations.^89^^,^^186^

When combined with metformin, oral AA 500 mg/day for 1 year was twice as likely to reduce HbA1c in patients with T2D than metformin alone (odds ratio [OR], 2.31; 95% CI, 1.87–4.42; *P* < .001), and more effective in reducing fasting blood sugar (FBS; mean FBS ± SD, 7.65 ± 2.52 to 5.18 ± 1.66 mmol/L; *P *< .01) and the risk of future diabetic complications.^88^ In a human RCT lasting 24 weeks, patients with T2D were initially randomized to a calorie-restricted (500 kcal/d) vegetarian diet or an isocaloric diabetic control diet for 12 weeks. Both dietary regimens were then combined with 12 weeks of aerobic exercise (60% maximal heart rate, 1 h, 4×/wk). The experimental group showed greater loss of visceral fat and lower oxidative stress markers. The vegetarian diet plus exercise was associated with a significant improvement in ascorbate, adiponectin, SOD, and GSH concentrations; insulin sensitivity (30% [95% CI, 24.5%–39%] vs 20% [95% CI, 14%–25%]; *P* = .04), and body weight (-6.2 kg [95% CI, -6.6 to -5.3 kg] vs -3.2 kg [95% CI, -3.7 to -2.5 kg]; *P* = .001) compared with the control diet plus exercise group.^185^ These findings were complemented by a subsequent RCT involving participants with T2D, which found oral AA oral supplementation (2 × 500 mg/d for 4 mo compared with placebo) abrogated skeletal muscle oxidative stress during hyperinsulinemia and significantly increased insulin-mediated glucose disposal (delta rate of glucose disappearance; ΔRd; *P* = .009) and peripheral insulin-sensitivity index (*P* = .046).^189^

### Role of GLUT10 in Diabetes and MetS

Mice with orthologous human GLUT10^G^^128^^E^ gene variants, which are more susceptible to HFD-induced obesity, insulin resistance, and glucose intolerance, achieved a greater improvement in MetS, visceral adipose tissue metaflammation, and fibrosis compared with wild-type mice after AA supplementation.^190^ Nondiabetic humans with GLUT10 mutations can gradually develop a diet–gene–related intermediate type of T2D. This is due to GLUT10 regulating intracellular ascorbate concentrations and ascorbate-dependent DNA demethylation, which positively regulates expression of CCAAT/enhancer-binding protein-alpha (CEBPA) and PPAR-γ and promotes adipogenesis.^190^ GLUT10 polymorphisms cause loss of function of the GLUT10 transporter for DHA, thus inhibiting maturation of preadipocytes and limiting fat storage capability under HFD feeding.

**Figure 4.**
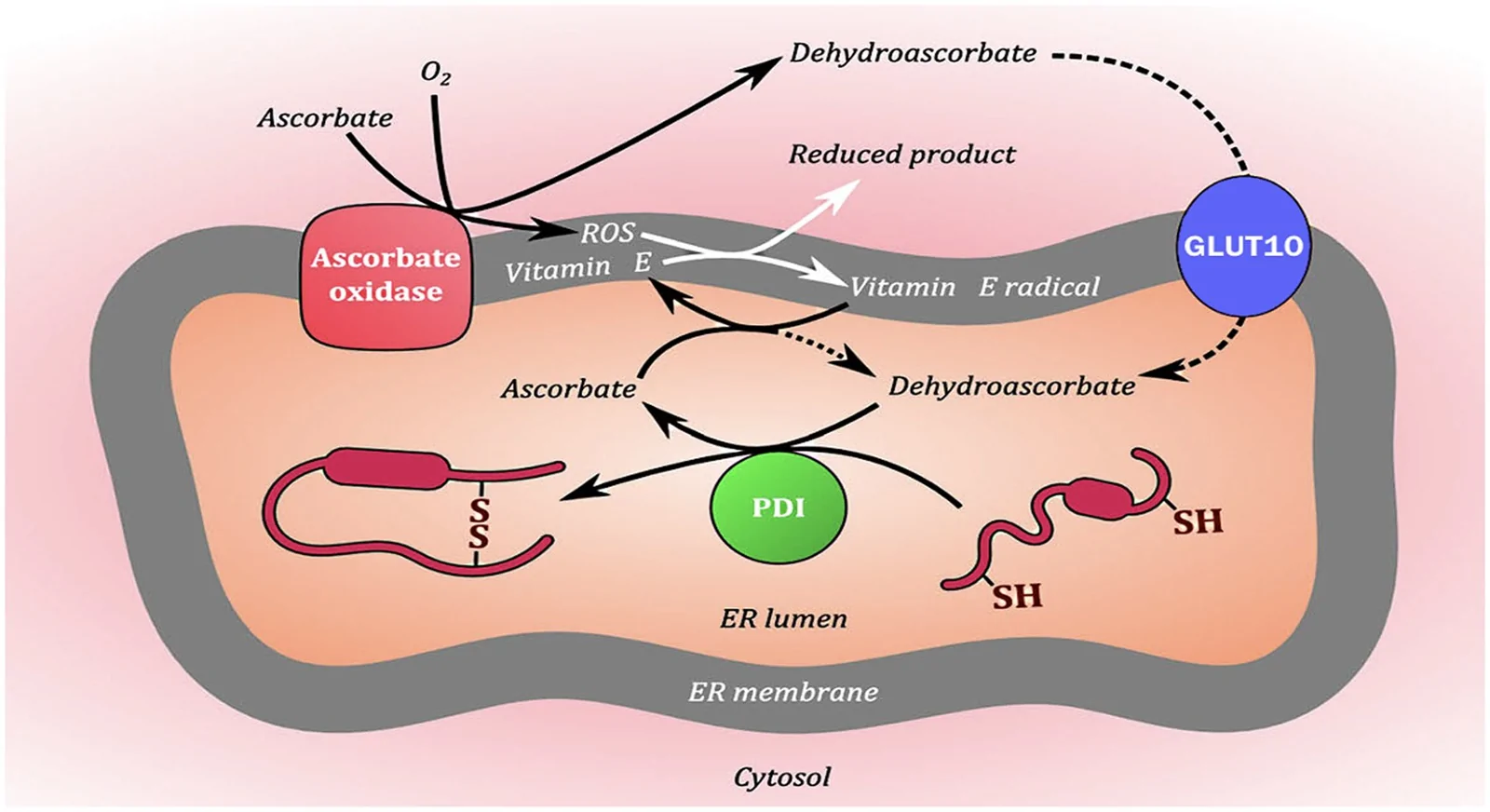
Hypothesized Role of DHA in Oxidative Protein Folding in the Endoplasmic Reticulum. Cytoplasmic ascorbate can be oxidized by an ascorbate oxidase localized in the ER membrane with the concomitant production of H_2_O_2_ and other ROS. The generated DHA can reach the ER lumen via GLUT10 transport. Tocopherol scavenges the ROS generated during the action of ascorbate oxidase. Tocopherol loses a hydrogen atom, and tocopheroxyl radical is formed. The tocopheroxyl radical is reduced by luminal ascorbate, while DHA is formed. ER DHA can be reduced by PDI oxidizing the active center dithiols of the enzyme. Oxidized PDI reacts with reduced nascent proteins yielding protein disulfides, allowing ER protein folding and regenerating PDI. Abbreviations: DHA, dehydroascorbate; ER, endoplasmic reticulum; GLUT10, glucose transporter 10; PDI, protein disulfide isomerase; ROS, reactive oxygen species, -SH, sulfhydryl group of the cysteine residue thiol; -SS-, disulfide bond between paired cysteine residues. Reproduced from Szarka and Lőrincz^134^ with permission.

In addition, GLUT10 is critical in reducing mitochondrial ROS levels and oxidative stress in mature adipocytes and vascular smooth muscle.^190^ Because mitochondria lack membrane SVCTs, they rely on GLUT10 to transport DHA from the cytosol. Dehydroascorbate is then regenerated to ascorbate in the mitochondria by GSH, thioredoxin, or GRX5. GLUT10 is co-localized with PDI in the ER, which is also important in regulating ER protein folding and ER stress^190^ (**Figure 4**). The absence of ER GLUT10 in humans leads to lack of ascorbate-facilitated hydroxylation of prolyl and lysyl residues in elastin and collagen and failure of their maturation in the ER (arterial tortuosity syndrome). It is hypothesized that ascorbate consumption by collagen prolyl hydroxylation in the ER also decreases the availability of ascorbate in the nucleus for DNA and histone demethylases. This may prevent the differentiation of pluripotent stem cells into mesenchymal cells or adipocytes, thus potentially limiting the expandability of subcutaneous adipose tissue (SAT) storage capacity.^191^ Transport of DHA into the nucleus by GLUT10 is also important for scavenging superoxides, as the nucleus lacks SOD activity, and, like the mitochondria, also lacks the enzymes required for the subcompartmental synthesis of GSH.^117^^,^^192^

**Figure 5.**
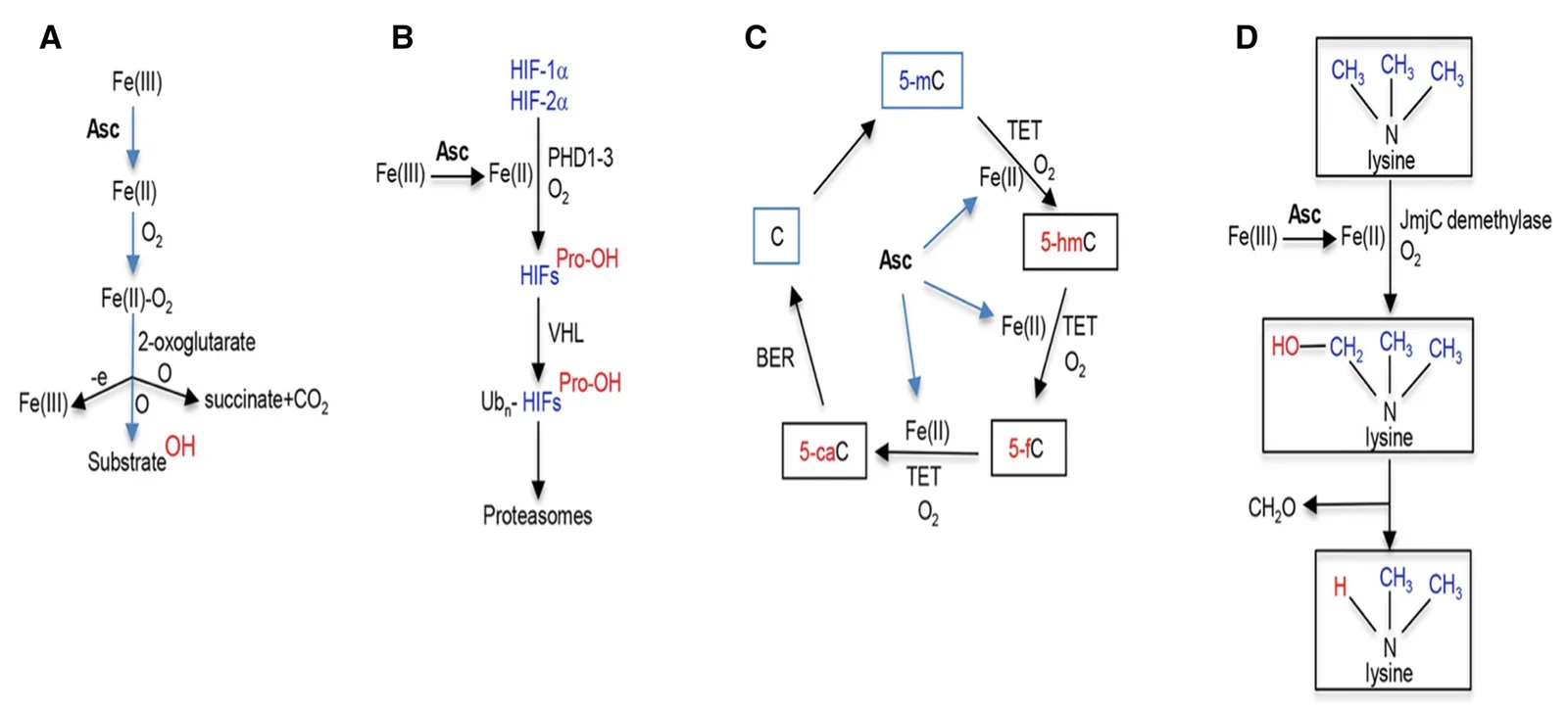
Role of Ascorbate in the Activity of Fe(II)/2-Oxoglutarate-Dependent Dioxygenases. (A) General mechanism of oxidative hydroxylation by Fe(II)/2-oxoglutarate–dependent dioxygenases. (B) Constitutive degradation of hypoxia-inducible transcription factors HIF-1α and HIF-2α in normoxic cells. (C) Removal of genomic 5-methylcytosine (5-mC) by a sequential oxidation of the methyl group by TET enzymes. (D) Role of ascorbate in catalyzing Jumonji histone demethylation of histone lysines. Abbreviation: Asc, ascorbate; BER, base excision repair pathway; Fe(II), ferrous ion; Fe(III), ferric ion; 5-caC, 5-carboxylcytosine; 5-fC, 5-formylcytosine; 5-hmC, 5-hydroxylmethylcytosine; PHD1-3, HIF proline hydroxylase 1-3; Pro-OH, hydroxylated proline residue in HIF-α; TET, ten-eleven translocation methylcytosine dioxygenase; Ub_n_-HIFs, Ubiquitinated HIF-αs; VHL, von Hippel-Lindau protein. Reproduced from Zhitkovich^117^ with permission.

**Figure 6.**
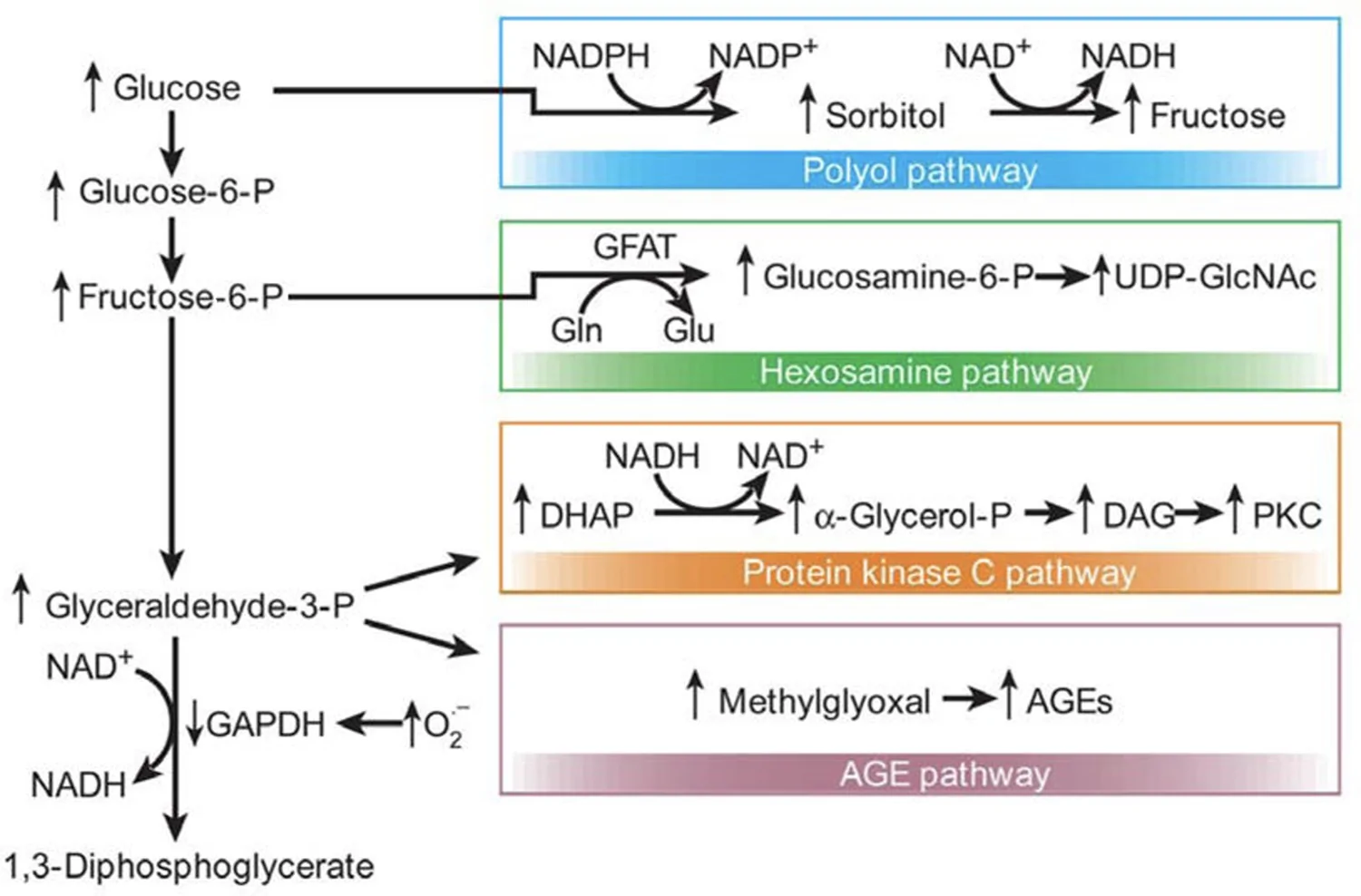
Flux of Glucose Into Alternative Pathways When Cytosolic GAPDH Is Inhibited by Peroxides From Mitochondrial Superoxide Dismutation, Due to Mitochondrial Oxidative Stress During Glucotoxicity or Subcompartmental Ascorbate Deficiency. These include polyol, hexosamine, protein kinase C, and AGE pathways, which further worsens cellular oxidative stress and metabolic syndrome. Abbreviations: AGE, advanced glycation end-product; DHAP, Dihydroxyacetone phosphate; GAPDH, glyceraldehyde-3-phosphate dehydrogenase; GFAT, glutamine:fructose-6-phosphate amidotransferase. Reproduced from Rahangdale et al.^178^ with permission, and from the original with permission of Springer Nature, from Brownlee M. Biochemistry and molecular cell biology of diabetic complications. Nature. 2001 Dec 13;414(6865):813-20. doi:10.1038/414813a; permission conveyed through Copyright Clearance Center, Inc.

## ASCORBIC ACID AND THE GUT–LIVER AXIS

### Cholesterol Excretion, Gallstones, and NAFLD

Cholesterol excretion via bile acids is the most important process by which humans excrete cholesterol. Metabolic syndrome is associated with the development of gallstones (OR = 1.61; *P* < .0001), and causation was recently confirmed in a Mendelian randomization study of patients with European ancestry.^193^^,^^194^ Hepatocyte synthesis of primary bile acids from cholesterol involves the mixed-function heme oxidase cytochrome P450 enzyme cholesterol 7α-hydroxylase (CYP7A1). This is the first and rate-limiting step of the classic pathway of bile salt biosynthesis, utilizing AA as a cofactor. The transcription of CYP7A1 is tightly regulated by ligand-activated transcription factors, bile acid responsive elements (BAREs), DNA methylation, histone acetylation, and environmental inputs including the intestinal microbiome, dietary nutrients, and diurnal rhythm.^195^^,^^196^ Long-term AA deficiency in guinea pigs leads to decreased hepatic cholesterol excretion, hypercholesterolemia, lipotoxicity, and accumulation of cholesterol in hepatocytes, manifested by hepatocyte ballooning, hepatic steatosis, and NASH.^91^^,^^103^^,^^197^ The effects of AA deficiency are more pronounced in animals fed an atherogenic diet.^197^ Loss-of-function polymorphisms of the *CYP7A1* gene in humans is associated with high total cholesterol and LDL-cholesterol and low HDL-cholesterol plasma levels, T2D, accelerated atherosclerosis, acute coronary syndrome, hepatic steatosis, and gallstone disease.^198^^,^^199^

Cholesterol supersaturation in bile promotes the formation of cholesterol microcrystals and eventually gallstones, which is observed in AA-deficient animals and is related to a low rate of cholesterol-7α-hydroxylation.^90^ Supplementation of AA can improve cholesterol excretion from the liver by increasing CYP7A1 activity in the conversion of cholesterol to the primary bile acids—cholic acid and chenodeoxycholic acid.^200^ Ascorbic acid supplementation in guinea pigs resulted in a 15-fold increase in the activity of cholesterol-7α-hydroxylase compared with AA-deficient guinea pigs.^94^ However, this effect was not demonstrated in a 1983 human trial of AA depletion and repletion.^93^ Experimental AA deficiency (plasma AA range, 8.0–13.3 µmol/L) in humans had no overall effect on biliary lipid composition and saturation index of gallbladder bile; plasma cholesterol and triglycerides; plasma cholesterol in HDL, very-low-density lipoprotein (VLDL), and LDL fractions; pool size, fractional turnover or new synthesis of either cholic or chenodeoxycholic acids; output of fecal acid or neutral sterols; or fecal sterol balance, although responses varied widely between individuals.^93^ Similarly, a study of patients having elective cholecystectomy for cholesterol gallstones found that preoperative AA supplementation (500 mg PO 4 times/d) for 2 weeks did not change the activity of cholesterol-7α-hydroxylase, 3-hydroxy-3-methylglutaryl coenzyme A (HMG-CoA) reductase, or the plasma lipid profile. However, compared with the control group who did not receive AA, AA supplementation significantly decreased cholic acid and increased deoxycholic acid (DCA), ursodeoxycholic acid (UDCA), and lithocholic acid (LCA) proportions in gallbladder bile (*P* < .05); increased bile phospholipids by 11% (*P* < .01); and significantly prolonged cholesterol nucleation time from 2 to 7 days (*P* < .01).^91^

In humans, an observational study involving 2129 randomly selected participants showed that regular AA supplementation is associated with a significantly decreased risk of gallstones (OR, 0.34; 95% CI, 0.14 to 0.81; *P *= .01).^94^ Long-term AA supplementation (800 mg/d) for 6 months in humans eventually resulted in negative feedback effects on CYP7A1 activity, due to recycling of the increased bile acid pool via the enterohepatic circulation.^201^ This could be effectively suppressed, and the LDL-cholesterol lowering effect maintained, by the addition of dietary fiber, such as citrus pectin (15 g/d), to AA supplementation. Pectin, like cholestyramine, acts as a bile acid sequestrant to disrupt bile acid reabsorption from the terminal ileum.^201^ The negative feedback effect of re-absorbed hydrophobic bile salts in humans is mediated by activation of farnesoid X receptor (FXR)/FGF19/FGFR4 signaling in hepatocytes, and also stimulation of cytokine release (TNF-α, IL-1β) from Kupffer cells.^196^

### Ascorbic Acid, Endotoxins, Gut Permeability, and the Gut–Liver Axis

A Western diet is associated with intestinal dysbiosis and increased intestinal permeability, particularly in obesity (metabolic endotoxemia).^202^ Human consumption for 4 weeks of a fast-food-style Western diet consisting of a high-fat content (40% of total calories) with high saturated fats (20.8% of total calories) and low fiber (12.5 g/d) resulted in a significant increase (71%) in postprandial endotoxemia compared with baseline.^202^ Conversely, 4 weeks of a “prudent diet” consisting of low fat content (20% of total calories), low saturated fats (5.8% of total calories), and high fiber (31 g/d) was associated with significant decreases in endotoxemia (38%) and serum TNF-α (17%). Prudent-style diets have greater levels of vitamin C, vitamin D, omega-3 fatty acids, complex carbohydrates, and fermentable fiber.^202^ High-fat diets increase the proportion of gram-negative bacteria such as *Enterobacter cloacae*, *Escherichia coli*, and *Klebsiella pneumoniae,* which promote intestinal endotoxin absorption. In contrast, high-fiber plant-based diets generally increase the numbers of anaerobic gram-positive bacteria, which ferment prebiotics, such as Bifidobacteria, Anthobacteria, and *Lactobacilllus* spp, resulting in decreased endotoxemia. Generation of short-chain fatty acids (SCFAs) by bacterial fermentation of dietary fiber decreases the colonic pH and suppresses the growth of pathogenic bacteria, including *E coli*, *Salmonella*, and *Campylobacter* spp.^203–207^ In an RCT involving humans with a BMI of 18–30 kg/m^2^, oral vitamin C supplementation (500 mg/d) formulated for targeted colon delivery improved the alpha diversity of colonic microbiota, reduced fecal pH and redox potential, and significantly increased the fecal concentrations of SCFAs (butyrate and propionate) when compared with baseline and control patients.^208^^,^^209^

Endotoxins are mainly derived from lipopolysaccharides (LPS) of gram-negative bacteria. The major lipidic component of LPS is the microbial outer membrane Lipid-A, which is a pathogen-associated molecular pattern (PAMP) and a ligand for the pattern recognition receptor Toll-like receptor 4 (TLR-4) and its co-receptors CD14 and myeloid differentiation 2 (MD-2). Because saturated fatty acids (SFAs), including lauric and myristic acids, are part of the Lipid-A molecule, a high-saturated-fat diet can also stimulate TLR-4 receptors in enterocytes, immune cells, and hepatocytes.^207^ Modifying the fat component of the diet by substituting SFAs with fish oils rich in omega-3 (n-3) polyunsaturated fatty acids (PUFAs) significantly reduced porcine ex vivo intestinal mucosal to serosal endotoxin transport permeability by 50% and postprandial systemic endotoxemia in an in vivo porcine model. The protective effect of fish oils was not produced by vegetable or olive oils.^210^ This is related to n-3 PUFAs changing gut bacterial biodiversity and enterocyte TLR4-mediated transcellular transport of LPS and live bacteria by lipid rafts and endocytosis, as well as alteration of the fatty acyl chains on Lipid-A and its immunogenicity. High-fat diets increase the incorporation of endotoxins into apolipoprotein B48 chylomicrons by enterocytes, which enhances postprandial endotoxin absorption. It has also been shown that SFAs disrupt enterocyte tight junctions and enhance paracellular permeability of macromolecules, including endotoxins.^211^ Individuals with obesity (mean BMI, 36.6 kg/m^2^) in comparison to normal-weight individuals (mean BMI, 24.3 kg/m^2^) had significantly higher postprandial serum endotoxin concentrations and cytokines (CRP, IL-6) on a high-fat diet (45.5%; 39.6 g SFAs/d) than on a low-fat diet (25.9%; 12.8 g SFA/d).^211^ Endotoxemia is enhanced by the diminished synthesis of zonula occludens-1 and -2 (ZO-1, ZO2) tight junction binding proteins in patients with obesity/NAFLD and increased gut permeability, and inhibited by the maintenance of colonocyte tight junction barrier function with healthy high-fiber diets, which generate microbial-derived SCFAs such as butyrate (reviewed in Portincasa et al^212^).

Lipopolysaccharides impair the absorption of AA by decreasing the expression of both SVCT1 and SVCT2, and a high intake of glucose inhibits the intestinal absorption of DHA by brush border GLUT.^213^ This may partly explain the lack of improvement in serum AA concentrations after AA supplementation without intensive dietary intervention in patients with obesity. Impaired intestinal barrier function is a feature of scurvy, and translocation of gram-negative bacteria across the intestinal epithelium is inhibited in vitro by AA, potentially by catalyzing collagen synthesis.^213^ Endotoxemia in humans is also increased after strenuous exercise, which is abrogated by oral AA supplementation (1000 mg).^213^

Ascorbic acid supplementation changes gut microbiota composition and improves gut barrier integrity, which can alter intestinal FXR signaling and also prevent LPS absorption into the portal vein.^95^ Portal vein endotoxins activate TLR-4 receptors in hepatocytes, Kupffer cells, and hepatic stellate cells, leading to activation of NF-κB signaling, release of proinflammatory cytokines and ROS, and escalation of liver injury in NAFLD. Obesity-related hyperleptinemia can also result in hepatic hyperresponsiveness to endotoxins, as leptin increases expression of the endotoxin receptor CD14 in Kupffer cells. This may provoke the progression of simple hepatic steatosis to NASH in children and adults, as measured by hepatic fibrosis, portal inflammation, and retention of triglycerides in hepatocytes (ballooning).^213^^,^^214^ LDL and chylomicron remnants with their sequestered endotoxins are endocytosed by hepatocyte LDL receptors (LDLRs), a process impaired by the serine protease proprotein convertase subtilisin/kexin 9 (PCSK9). Ascorbic acid inhibits PCSK9 transcription, thus increasing LDLR number and activity, and by inference improving lysosome degradation of endotoxins and clearance by hepatocytes. This may be beneficial in obese patients with increased gut permeability, chronic endotoxemia, NAFLD, and hepatic insulin resistance, but remains to be proven.^215^ Currently, the normal range of plasma PCSK9 in patients not on fibrates or statins is 170–220 ng/mL, with *E coli* endotoxin clearance being impaired at PCSK9 serum concentrations above 250 ng/mL in cultures of human hepatocytes.^216^

Ascorbic acid and/or vitamin D supplementation inhibited bile acid salt reflux–related apical sodium-dependent bile acid transporter (ASBT) and activated bile acid synthesis (via CYP7A1), FXR, and bile acid transportation–related bile salt export pump (BESP) in the gut–liver axis of male C57BL/6 mice.^95^ FXR was shown to inhibit hepatic fatty acid synthase (FAS) mRNA and protein expression of FAS. Levels of MDA and proinflammatory cytokines (TNF-α, IL-1β, and IL-6) were also reduced in the liver and ileum. The above effects on the gut–liver axis were thought to contribute to the improvement in or prevention of NAFLD in HFD-induced obesity in C57BL/6 mice by AA supplementation.^95^ In humans with MetS, decreased enterocyte chylomicron and hepatic VLDL incorporation of dietary α-tocopherol is also related to high-fat diets, AA deficiency, and intestinal inflammation. This decreased α-tocopherol bioavailability, as well as impaired regeneration of α-tocopherol from α-tocopherol free radicals due to inadequate AA, may contribute to lipid peroxidation, hepatic oxidative stress, insulin resistance, hyperglycemia, and dyslipidemia.^213^

### Ascorbic Acid, Fatty Acid β-Oxidation, and Hepatic Steatosis

Impaired fatty acid oxidation and accumulation of hepatic triglycerides/SFAs derived from visceral adipocytes contribute to obesity, NAFLD, T2D, and atherosclerosis.^8^^,^^9^ Ascorbic acid significantly increases the level of PPAR-α in HFD-fed C57BL/6J mice.^35^^,^^217^ PPAR-α is a ligand-activated transcription factor for fatty acyl-CoA oxidase (ACOX), CPT-1, medium-chain acyl-coenzyme A dehydrogenase (MCAD), and very-long-chain acyl-coenzyme A dehydrogenase (VLCAD). PPAR-α thus increases circulating triglyceride hydrolysis, fatty acid transport, and fatty acid β-oxidation (in mitochondria, peroxisomes, and microsomes).

PPAR-γ is an important stimulator of adipogenesis and is involved in the regulation of hepatic lipogenesis and glucose metabolism. PPAR-γ was decreased when steatotic human hepatoma (HepG2) cells were treated with AA.^71^ Ascorbic acid also increased adenosine 5′-monophosphate kinase (AMPK) phosphorylation in HepG2 cells in vitro, and in the liver from HFD obese C57BL/6J mice.^218^ Liver X receptor α (LXRα) is a nuclear receptor regulating fatty acid synthesis in the liver, which contributes to the development of liver steatosis.^218^ In both in vivo and in vitro models, LXRα nuclear translocation was inhibited by increased AMPK phosphorylation through AA supplementation. Decreased LXRα expression subsequently suppressed lipogenic genes, including SREBP1c, acetyl-CoA carboxylase 1 (ACC1), and fatty acid synthase (FASN). Ascorbic acid supplementation thus decreased hepatic lipogenesis and hepatic lipid accumulation.^97^^,^^218^ Interestingly, in HFD-fed SMP30 KO mice, AA deficiency led to suppressed hepatic lipogenesis by impairment of nuclear translocation of SREBP-1c, despite increased cytoplasmic levels of SREBP-1c.^102^ This delayed the progression of hepatic steatosis to NASH. It was thought that the inhibition of both SREBP-1c activity and de novo hepatic lipogenesis was due to hepatocyte accumulation of cholesterol during long-term AA deficiency. Hepatocyte cholesterol accumulation was reversed by AA supplementation in AA-deficient, HFD-fed SMP30 KO mice, which allowed nuclear translocation of SREBP-1c but promoted cholesterol excretion in bile.^102^ Cholesterol-7α hydroxylase is required for 90% of bile acid synthesis from cholesterol in humans^102^ (**Table 1**).

### Ascorbic Acid, LDL, and Cholesterol Homeostasis

As a prelude to the excretion of cholesterol in bile, the liver is involved in the capture and clearance of excess circulating LDL cholesterol.^92^ This is achieved by LDL binding to LDLRs on the hepatocyte cell membrane, allowing LDL endocytosis and catabolism by hepatocyte lysosomes. The expression of LDLR is decreased in hepatocytes and its intracellular degradation is increased by PCSK9, resulting in elevated serum LDL-cholesterol concentrations. PCSK9 reduces the number of recycled LDLRs, which normally are recycled up to 150 times to the hepatocyte cell membrane after delivering LDL to lysosomes. PCSK9 expression is inhibited by adiponectin receptor agonists in apolipoprotein E null (ApoE^−/−^) mice but not in wild-type mice fed a high-fat Western diet. ApoE^−/−^ HFD mice provide a murine model for human hypercholesterolemia, atherosclerosis, NASH, and MetS, as wild-type HFD mice, in contrast to humans, carry most of their cholesterol as HDL cholesterol not LDL cholesterol. PCSK9 expression is promoted by leptin and resistin activation of STAT3 in HepG2 cells. Hyperleptinemia was significantly associated with increased plasma PCSK9 in humans with a BMI less than 25 kg/m^2^, but not those with a BMI greater than 25 kg/m^2^.^219^ PCSK9 is positively correlated with intracellular de novo lipogenesis enzymes, including diacylglycerol acyltransferase 2 (DGAT2), FAS, and stearoyl-coenzyme A desaturase (SCD), and degree of hepatic steatosis in animal and human studies.^220^

In human HepG2, Huh7, and murine C57BL/6J primary hepatocytes, LDLR activity was enhanced and PCSK9 transcription was reduced by AA treatment, demonstrating that the beneficial effect of AA is not species dependent.^92^ In GLO-deficient (GLO^**−/−**^, C57BL/6J background) mice fed an HFD, AA deficiency was associated with increased serum PCSK9 concentrations and hepatic PCSK9 expression, resulting in reduced hepatic LDLR expression, significantly increased serum LDL, and decreased serum HDL and HDL-to-LDL ratios. This was reversed by 2 weeks of AA supplementation (intraperitoneal [i.p.], 100 mg AA/d/kg body weight), which also increased the activity of SREBP2 and FoxO3a. Serum PCSK9 decreased from 137 ± 5 ng/mL to 94 ± 6 ng/mL (*P* < .01) after AA replacement in the GLO^**−/−**^ mice.^92^ The AA-induced increase in FoxO3a activity resulted in inhibition of hepatocyte nuclear factor 1a (HNF-1a) binding to the promoter of PCSK9 transcription, and activation of LDLR protein expression. Ascorbic acid activation of the SREBP2 pathway resulted in promotion of LDLR transcription. In a human cohort, serum AA concentrations had significant negative correlations with serum PCSK9 (*r* = -0.610; *P* = .0015), total cholesterol (*r* = -0.448; *P* = .028), and LDL (*r* = -0.421, *P* = .041).^92^

### Ascorbic Acid and Progression of NAFLD to NASH Fibrosis

Ascorbic acid can prevent liver fibrosis by suppression of hepatic stellate cell (HSC) proliferation and H_2_O_2_-induced type I collagen (Col1a1) expression, which was observed in in vitro studies of human HSC LX-2 cells and rat primary HSCs.^99^ However, AA can enhance collagen synthesis by HSCs in vivo due to increased procollagen prolyl hydroxylase activity, and potentially promote liver fibrosis in NAFLD. This may be related to hepatocytes having both SVCT1 and SVCT2 transporters, and the sole AA transporter (SVCT2) in HSCs being upregulated in human NASH cirrhosis.^96^^,^^221^

It has been proposed that the effect of AA is dependent on the stage of NAFLD.^102^ In hepatic steatosis, AA mainly regulates de novo lipogenesis and maintains lipid homeostasis, while in NASH, AA exerts its antioxidant effect to ameliorate oxidative stress–induced hepatic inflammatory lesions.^102^ Higher AA plasma concentrations (60.2 vs 39.2 µmol/L) are associated with a lower risk of NAFLD (OR = 0.59; 95% CI, 0.48–0.74; *P* < .001), after adjustment for other variables. This applies to both women (OR = 0.63; 95% CI, 0.49–0.80; *P* < .001) and men (OR = 0.73; 95% CI, 0.55–0.97; *P* = .03). However, a causal association could not be demonstrated in a Mendelian randomization study.^222^ This used multivariable logistic regression analysis of 5578 individuals with complete serum AA levels and NAFLD results in US National Health and Nutrition Examination Surveys (NHANES) 2005–2006 and 2017–2018, based on genetic data from large-scale human genomewide association studies.^222^

### Nonalcoholic Fatty Liver Disease, Ferroptosis, and AA

Nonalcoholic fatty liver disease is associated with hepatic oxidative stress, increased iron deposition, lipid peroxidation markers (MDA and 4-hydroxynonenal), and ferroptosis.^223^ Ferroptosis promotes hepatocyte death by Fenton reaction–mediated lipid peroxidation and the progression of hepatic steatosis to NASH.^223^ This is, in part, related to depletion of GSH and decreased activity of glutathione peroxidase 4 (GPX4). Ascorbic acid can increase non-heme iron absorption from a single meal in a dose-dependent way, but not from a complete diet.^224^ Removal of iron by chelation (deferoxamine) or phlebotomy may assist in the hepatic iron overload of NAFLD, and whether AA improves or worsens iron overload and ferroptosis is an important management question.^223^^,^^225–229^

**Figure 7.**
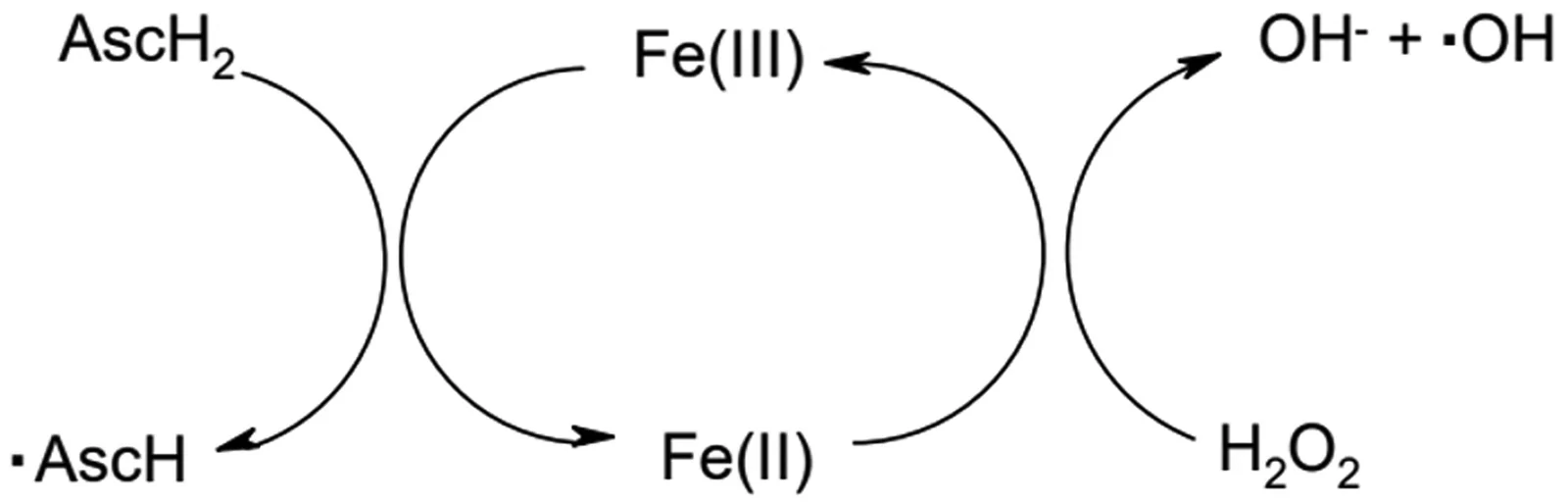
Fenton Reaction. Role of AA (AscH_2_) in the reduction of ferric ion [Fe(III)] to ferrous ion [Fe(II)] with generation of ascorbyl radical (•AscH), coupled with formation of hydroxyl radicals (•OH) from hydrogen peroxide (H_2_O_2_). Abbreviation: AA, ascorbic acid. Reproduced from Timoshnikov et al.^229^ with permission.

Under normal physiological conditions, iron is bound in its stable ferric form by proteins such as transferrin, ferritin, and neutrophil gelatinase–associated lipocalin.^226–229^ Ascorbate concentrations between 1.2 and 1.7 mmol/L provide half-maximal rates of iron release from ferritin, potentially elevating cellular concentrations of free or labile iron pool. In the presence of further ferrous ions, peroxides are converted to damaging hydroxyl radicals by the Fenton reaction. This can cause peroxidation of PUFA chains of membrane phospholipids, resulting in organelle or plasma membrane rupture and ferroptosis^230^ (**Figure 7**).

Ascorbic acid can spontaneously oxidize when exposed to free iron that is not bound in a redox inert state in proteins.^127^ Ascorbate is known to protect LDL against oxidation by copper, iron, peroxynitrate, neutrophils, or cigarette smoke. However, in vitro at a lysosomal pH = 4.5, ascorbate (30 µmol/L) may not protect against copper-induced LDL oxidation as it successfully does at pH = 7.4. In addition, DHA at lysosomal pH markedly enhanced LDL oxidation by ferritin, suggesting that high levels of oxidative stress, ascorbate oxidation, and lysosomal activity of macrophages in NAFLD/NASH may indeed promote hepatic ferroptosis.^73^

Nonalcoholic fatty liver disease is associated with hepatic iron overload and elevated serum ferritin concentrations.^231^ This could lead to the promotion of AA auto-oxidation and subsequent H_2_O_2_ formation and oxidative stress.^101^ However, an in vitro study with HepG2 cells showed that the provision of AA alleviated palmitic acid/oleic acid–induced intracellular lipid retention via ferritin suppression and labile iron pool induction.^232^ Even in the presence of iron overload, AA suppressed lipid peroxidation in vivo, and iron overload with low ascorbate concentrations was associated with elevated plasma triglycerides.^233^ Currently, to our knowledge, there is no clear evidence showing that oral AA supplementation leads to hepatic ferroptosis in humans.^232^^,^^234^

### Nonalcoholic Steatohepatitis, Neutrophils, Foam Cells, and AA

**Figure 8.**
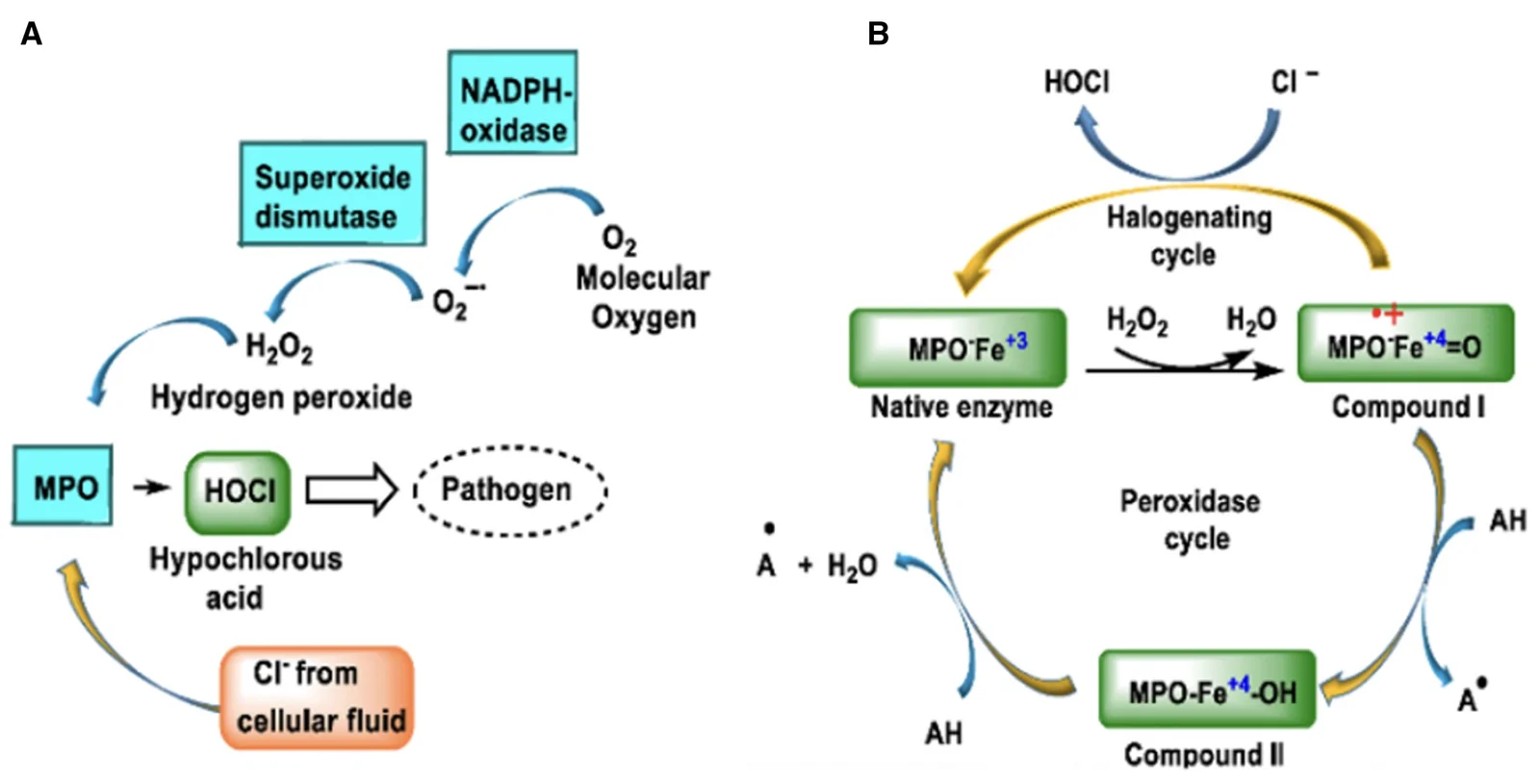
Normal Catalytic Cycling of Granulocyte Myeloperoxidase Involving Ascorbate. (A) Schematic representation of HOCl production during the respiratory burst. The leukocyte respiratory burst involves reducing molecular oxygen to reactive oxygen intermediates (ROS), such as superoxide radicals (•$O_{2}^{-}$), via NAD(P)H oxidase. The superoxide undergoes a disproportionation reaction by SOD to give oxygen (O_2_) and hydrogen peroxide (H_2_O_2_). MPO deposited in the azurophilic granules catalyzes the reaction of H_2_O_2_ and chloride anion (Cl^−^) to form hypochlorous acid (HOCl) and other hypohalous acids. HOCl is a relatively strong oxidizing agent and toxic to bacteria. It also causes damage to host cells in the case of prolonged and extended MPO activation. (B) The native state of the neutrophil enzyme, ferric MPO, reacts with hydrogen peroxide to form the redox intermediate compound I. Compound I oxidizes chloride to regenerate ferric MPO via the halogenation cycle. Compound I can be reduced to compound II by an excess of H_2_O_2_ or by the presence of a reducing agent (ascorbate). This compound II, although quite stable, can be reduced to ferric MPO in the presence of a reducing agent (ascorbate) or the superoxide radical or it can also be oxidized to compound III under high concentrations of H_2_O_2_. On the other hand, native MPO reacts with superoxide to give compound III. Abbreviations: AH, ascorbic acid; A^•^, ascorbate radical; MPO, myeloperoxidase; NAD(P)H, nicotinamide adenine dinucleotide (phosphate), reduced form; ROS, reactive oxygen species; SOD, superoxide dismutase. Reproduced from Andrés et al.^236^ with permission.

Progression of NASH to fibrosis is also related to hepatic infiltration by activated neutrophils and the release of heme peroxidase enzymes, such as neutrophil MPO.^235^ This is specific to diet (high-fructose, high-medium-chain *trans* fats/cholesterol)–induced NASH but is not essential in other causes of hepatic fibrosis, such as carbon tetrachloride injury or bile duct ligation in murine models.^235^ In human patients, those with biopsy-proven NAFLD had moderately increased MPO, but those with NASH had greatly increased plasma MPO (40.7 ng/mL vs 16.1 ng/mL; *P* = .0044), when compared with healthy-liver control patients (with normal liver function tests and hepatic elastography). Increased gene expression of MPO in liver biopsies from patients with NAFLD/NASH was also significantly correlated with increasing BMI and impaired glucose homeostasis (HbA1c). MPO produces the oxidizing agents hypochlorous acid (HOCl), hypobromous acid (HOBr), or hypothiocyanous acid (HOSCN) from H_2_O_2_ via Fenton reactions, and AA at low micromolar concentrations acts as a substrate for the MPO enzyme.^38^^,^^236^^,^^237^ Neutrophil chemotaxis into the liver is thought to be related to hepatic expression of CXC chemokines IL-8 and GRO-α. IL-8 secretion is increased by abnormal triglyceride accumulation in hepatocytes, and also by HOCl modification of lipoproteins, creating a feed-forward effect of increasing inflammation, neutrophil recruitment, MPO release, and lipoprotein oxidation during NAFLD^235^^,^^238^ (**Figure 8**).

Ascorbic acid at higher concentrations can protect against the damaging effects of neutrophil-derived HOCl or chloramine when they are released from phagolysosomes into the neutrophil cytoplasm or extracellular space, preventing protein and DNA damage from halogenation and lipoprotein oxidation (ox-LDL). The lipid moiety of LDLs (especially PUFAs) can be oxidized by metal cations (iron [Fe^3+^] and copper [Cu^2+^]), lipoxygenases, and reactive nitrogen species, while the protein moiety of LDL (apoB-100) is targeted by HOCl and MPO. MPO oxidation of LDL (mox-LDL) prevents the recognition of apoB-100 by the LDL receptor, and mox-LDL is then scavenged by endothelial macrophages and smooth muscle cells. These lipid-laden cells in atherosclerotic plaques were first described as “foam cells” by Rudolf Virchow in 1858 and may also involve hepatic sinusoidal endothelium and Kupffer cells.^237^^,^^239^ Accumulation of intracellular ox-LDL promotes cholesterol crystallization, PCSK9 expression, and the degradation of IκB-α (the inhibitor of NF-κB), activation of the NOD-, LRR- and pyrin domain-containing protein 3 (NLRP3) inflammasome, release of inflammatory cytokines (IL-8, TNF-α), and Matrix metalloproteinases (MMP) from macrophages and endothelial cells, endothelial inflammation and instability of atheromatous plaques, and formation of CLS in NASH.^237^^,^^240^

Inhibition of copper-mediated LDL lipid peroxidation and MPO-mediated LDL oxidation by vitamin C in vitro appears to be dose related.^241^^,^^242^ Vitamin E does not reduce the reaction rate of LDL oxidation mediated by MPO, but vitamin E does inhibit the CD36 scavenger receptor for ox-LDL on macrophages, endothelial cells, and arterial smooth muscle cells.^237^^,^^243^ The inhibitory effect of AA on the chlorination of lipoprotein tyrosine residues can be measured by decreased serum 3-chlorotyrosine concentrations.^243^ Chlorination of HDL by HOCl prevents direct binding of HDL to the transporter ABCA1, which inhibits the efflux of excessive intracellular cholesterol from macrophages. This furthers the formation of macrophage foam cells and progression of atherosclerosis and NAFLD/NASH.^235^^,^^236^^,^^244–246^

## ASCORBIC ACID IN THE MANAGEMENT OF OBESITY-RELATED CONDITIONS

### Ascorbic Acid and NAFLD—Cross-sectional Studies

The risk of liver steatosis and NASH fibrosis is associated with obesity, MetS, and low serum AA concentrations.^107^^,^^247^^,^^248^ From the cross-sectional US 2017–2018 NHANES data involving 4494 adult patients, higher serum AA concentrations had an inverse linear association with liver steatosis, fibrosis, and cirrhosis as measured by transabdominal ultrasound and liver transient elastography.^107^ The inverse association between serum AA level and odds of risk of liver fibrosis was also reported in several other cross-sectional studies of US populations with NAFLD from NHANES data,^247^^,^^248^ with a serum AA concentrations of 50.0 ± 27.8 μmol/L in patients without fibrosis and 39.2 ± 27.8 μmol/L in patients with fibrosis in a weighted linear regression model (*P* < .0001). It has thus been proposed that AA supplementation could achieve protective and therapeutic effects in patients with NAFLD.

However, conflicting results exist. A small cross-sectional Canadian study showed no difference in plasma AA concentration between healthy participants and patients with NAFLD.^105^ The effect of AA supplementation on steatosis and liver fibrosis may also depend on obesity status. A cross-sectional study involving 1926 participants showed a more significant protective effect of AA against NAFLD fibrosis as measured by transient elastography among overweight (OR = 0.38; 95% CI, 0.16–0.92) or obese (OR = 0.63; 95% CI, 0.43–0.92) patients.^248^ This association was not found among underweight or normal-weight patients.^248^ Another cross-sectional study involving 3471 participants aged 40–80 years found a significant inverse association between AA intake and NAFLD, but only in nonobese patients.^249^ Possible confounding factors include the high susceptibility of NAFLD among obese patients; variation in NAFLD/fibrosis diagnostic methods; the difference in cutoffs between steatosis, NASH, and liver fibrosis; and retrospective estimations of dietary AA intake.^250^

### Animal Models of AA Supplementation and NAFLD

The prophylactic and therapeutic effects of AA supplementation on NAFLD have been demonstrated in murine models.^98^^,^^99^ N-Mary rats with methionine- and choline-deficient diet-induced NASH subsequently treated with AA (30 mg/kg/d) for 8 weeks had reduced biochemical and histological features of NASH.^98^ Liver histological improvement was observed in guinea pigs with methionine- and choline-deficient diet–induced NASH treated with megadose AA (2.5 g/kg/d).^100^ Supplementation with low-dose AA (15 mg/kg/d) and medium-dose AA (30 mg/kg/d) in HFD-fed C57BL/6 mice reduced the risk of hepatic steatosis development. In the same study, administration of 30 mg/kg per day of AA significantly ameliorated HFD-induced steatosis. However, low-dose AA supplementation did not achieve improvement in existing liver steatosis. High-dose AA supplementation (90 mg/kg/d) did not successfully prevent or improve HFD-induced liver steatosis, and, in fact, aggravated liver steatosis and increased body weight in mice.^101^ This suggests a dose-dependent effect of AA supplementation on HFD-induced liver steatosis.

### Human Studies of AA Supplementation and NAFLD

Ascorbic acid supplementation has pleiotropic effects in human NAFLD, including increased plasma ascorbate, total and HMW adiponectin, and the improvement in insulin resistance, lipid handling, liver steatosis, hepatocellular injury, and intestinal microbiota diversity.^85^^,^^234^^,^^251^^,^^252^ In a cross-sectional study involving 40–70-year-old participants, only intakes of AA greater than 180 mg/day (with an average dietary caloric consumption of 2000 kcal/d) could achieve a protective effect against NASH.^251^ The AA supplementation dose was also inversely associated with the level of steatosis measured by SteatoTest^®^ (BioPredictive, Paris).^251^ In an RCT, 45 patients receiving daily treatment of a combined 1000 mg of AA and 1000 IU of vitamin E for 6 months vs placebo achieved significant improvement in liver fibrosis score based on pre- and post-treatment liver biopsies.^104^ A combination of AA (500 mg/d) plus vitamin E (800 mg/d) for 12 months achieved a significant reduction in alanine transaminase (ALT), liver steatosis score, and fibrosis score in adults with NASH.^106^ Another RCT showed a 71% decrease in odds of hepatic steatosis in a population with pre-existing NAFLD after administration of AA (1000 mg daily), vitamin E (1000 IU daily), and atorvastatin (20 mg daily).^253^ A recent double-blind RCT investigated the effect of AA monotherapy in patients with NAFLD.^85^ Daily supplementation of low-dose (250 mg/d), medium-dose (1000 mg/d), or high-dose (2000 mg/d) AA for 12 weeks each achieved significant improvement in liver function. However, the improvement in liver function was significantly higher in those patients receiving medium-dose AA (1000 mg/d) compared with the low- or high-dose AA groups^85^ (**Table 1**).

### Ascorbic Acid and Obesity/Metabolic Dysfunction

**Figure 9.**
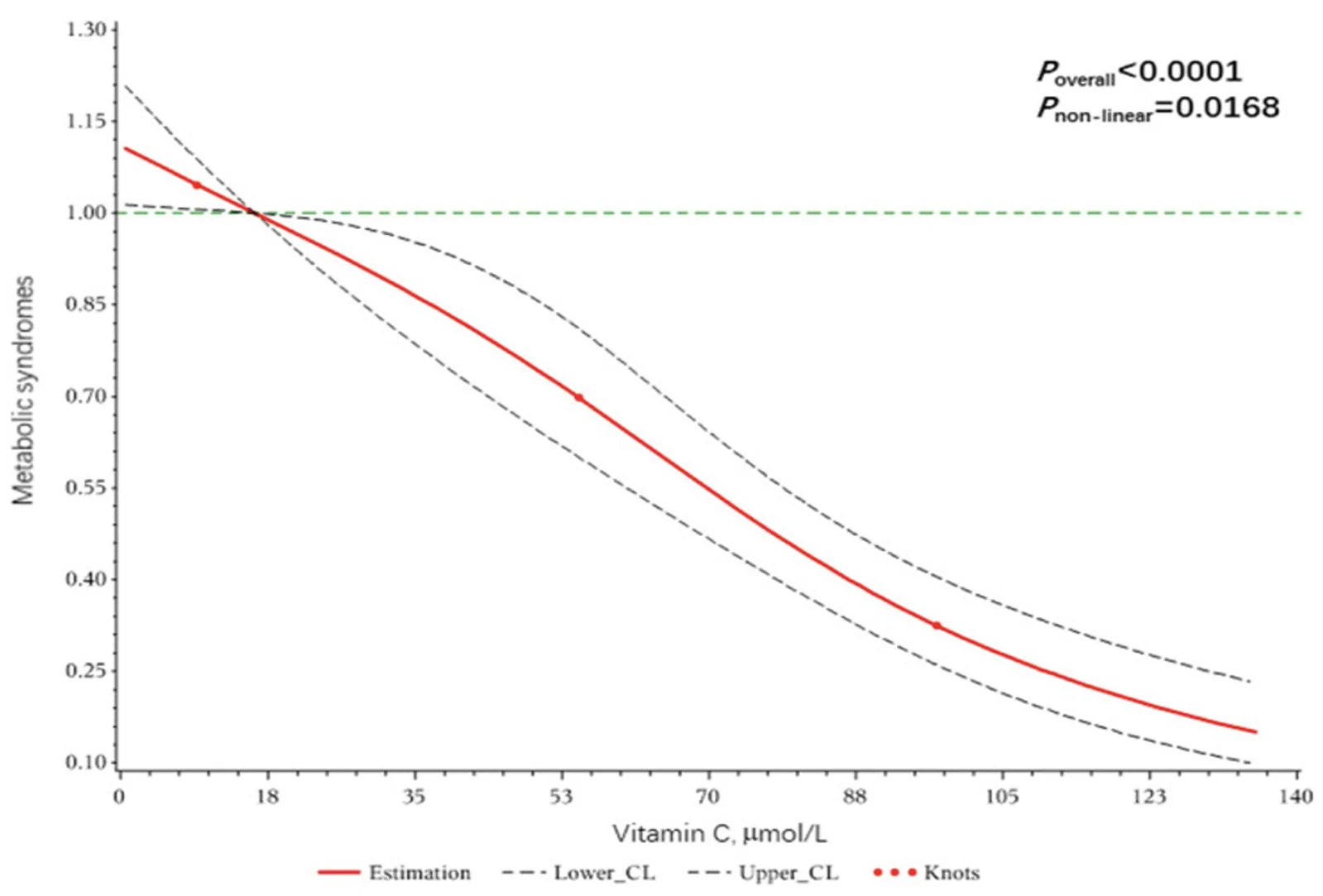
Dose–Response Relationship Between Serum Ascorbic Acid and Metabolic Syndrome Risk. Multivariate-adjusted logistic regression model analysis of the US 2003–2006 NHANES data. Abbreviations: CL, confidence limit; NHANES, National Health and Nutrition Examination Survey. Reproduced from Pei et al.^113^ with permission.

Cross-sectional studies have suggested an inverse relationship between serum AA concentrations and MetS.^254^ In the recent multivariate-adjusted logistic regression model analysis of the US 2003–2006 NHANES data, the third (OR = 0.67; 95% CI, 0.48–0.94) and highest (OR = 0.52; 95% CI, 0.35–0.76) quartiles of serum AA were found to be respectively associated with a 33% and 48% reduction in MetS risk.^113^ In the analysis of MetS components, higher serum AA quartiles were significantly associated with lower waist circumference (OR = 0.63; 95% CI, 0.43–0.93), plasma triglyceride (OR = 0.73; 95% CI, 0.55–0.96), blood pressure (OR = 0.63; 95% CI, 0.49–0.80), and fasting plasma glucose (OR = 0.70; 95% CI, 0.52–0.94), and higher AA quartiles were associated with higher HDL concentrations^113^ (**Figure 9**).

Retrospective cross-sectional studies have demonstrated an inverse association between plasma AA level and BMI/waist circumference.^110^^,^^160^ A meta-analysis of 28 observational studies of the relative risk of MetS found that the highest vs lowest AA intake (RR = 0.93; 95% CI, 0.88–0.97; *P *= .003) and highest vs lowest plasma ascorbate concentrations (RR = 0.60; 95% CI, 0.49–0.74; *P *< .001) had significant inverse associations with MetS^112^ (**Table 1**). A recent Mendelian randomization analysis in a Korean population of 64 409 adults found inadequate AA intake (<100 mg/d) to be significantly associated with MetS and its components hyperglycemia, hypertriglyceridemia, and hypertension on inverse-variance weighting but not MR-Egger testing.^181^ A prospective study involving 7569 Danish adults found no significant association between AA intake and change in BMI or waist circumferences in the general cohort.^111^ Only a borderline association between AA intake and decreased body-weight gain among the cohort of patients who were genetically predisposed to a high BMI was found.^111^ Patients with obesity or hypovitaminosis C require large doses of AA for long periods to achieve adequate serum AA concentrations, and this is not achieved by an oral AA intake of 75 mg per day.^255^

### Randomized Controlled Trials of AA Therapy in Human Obesity and MetS

A number of RCTs have investigated the use of AA as monotherapy for obesity or metabolic dysfunction in humans, with variable results reported (**Table 1**). An RCT involving 41 severely obese patients showed significant, but clinically modest, weight loss (2.5 ± 2.4 kg) in the group receiving 3 g of daily oral AA for 6 weeks, whereas the placebo-treated group lost 1.0 ± 1.7 kg (*P *< .01).^108^ The effect of 12 weeks of AA supplementation (500 mg/d) combined with physical endurance activity (30 min/d) was investigated in a double-blind RCT in 120 adult patients with MetS.^256^ Oral AA supplementation improved serum AA concentrations from the study baseline in the AA-supplemented group (49 ± 20 μmol/L to 74 ± 18 μmol/L; *P *< .05) and AA-plus-physical-activity group (46 ± 19 μmol/L to 84 ± 27 μmol/L; *P *< .05), with no significant change in baseline serum AA in the placebo or placebo-plus-physical-activity groups. There was no significant change in weight or BMI across the 4 groups after 12 weeks. The study suggested that a combination of AA supplementation and physical activity could achieve improved systolic blood pressure and serum level of total cholesterol compared with the placebo group. Ascorbic acid supplementation plus physical activity significantly decreased systolic blood pressure compared with the AA-only group (122 ± 10 vs 130 ± 9 mm Hg; *P *= .02).^256^

**Figure 10.**
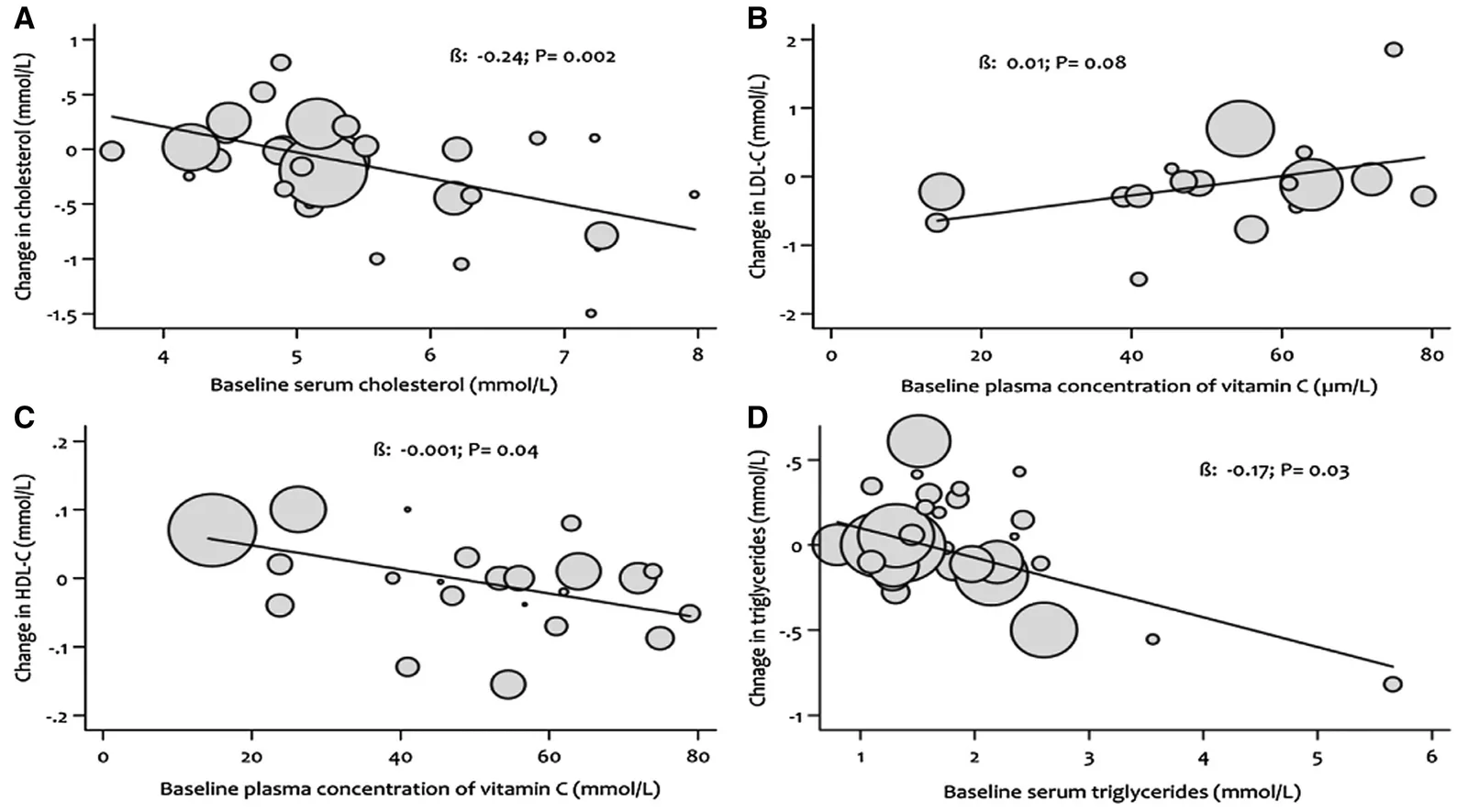
Associations Between Baseline Concentrations of Serum Lipids and of Ascorbic Acid (AA) and the Effect of AA Supplementation on Change in (A) Total Cholesterol, (B) Low-Density-Lipoprotein Cholesterol (LDL-C), (C) High-Density-Lipoprotein Cholesterol (HDL-C), and (D) Triglyceride Concentrations. The size of each circle is proportional to the number of participants in that study. Reproduced from Ashor et al.^260^ with permission.

A placebo-controlled RCT of daily supplementation with 1000 mg AA over 4 months in patients with T2D demonstrated a decrease from baseline in total-cholesterol (7.3 ± 0.5 vs 5.8 ± 0.4 mmol/L; *P *< .03), LDL-cholesterol (5.6 ± 0.6 vs 4.1 ± 0.3 mmol/L; *P *< .05), and triglyceride (2.6 ± 0.1 vs 2.1 ± 0.04 mmol/L; *P *< .04) concentrations.^109^ In contrast, a 4-week RCT of 800 mg AA/day vs placebo (500 mg citrate/d) in patients with T2D (*n* = 32) and obesity, a mean BMI of 34 ± 1 kg/m^2^, and mean plasma ascorbate of 23 ± 2 µmol/L did not show improvement in fasting glucose, insulin resistance, or endothelial dysfunction.^257^ However, ascorbate concentrations in the AA intervention group showed only partial replenishment (48 ± 6 μmol/L) after 4 weeks of supplementation, suggesting that the oral AA dose or the intervention period was inadequate.^257^ This contrasted with the predicted dose–response of a saturated plasma AA concentration of 80 μmol/L with equivalent supplementation in healthy patients. Partial replenishment of plasma AA was unable to improve the cardinal features of T2D in the patient cohort, including insulin resistance (HOMA-IR), elevated blood pressure, endothelial dysfunction, or peripheral vascular resistance. One suggested reason for this finding is that vitamin C saturation is required to improve NO-dependent endothelial activity, as AA can effectively compete with NO for superoxide anion scavenging and regenerate tetrahydrobiopterin at AA plasma concentrations of more than 80 μmol/L.^257^ Insulin also interacts with NO to facilitate microvascular perfusion via capillary and terminal arteriolar recruitment in muscles, which is important for glucose disposal and maintaining insulin sensitivity.^258^ A meta-analysis of 13 pooled RCTs in patients with hypercholesterolemia found that supplementation of at least 500 mg/day of AA for between 3 and 24 weeks resulted in a respective estimate effect in serum LDL cholesterol of -5% (-7.9 mg/dL; 95% CI, -12.3 to -3.5; *P *= .000), in HDL cholesterol of +2.3% (+1.1 mg/dL; 95% CI, -0.2 to 2.3; *P* = not significant), and triglycerides of -8.8% (-20.1 mg/dL; 95% CI, -33.3 to -6.8; *P *< .003).^259^ The authors concluded the AA-associated -0.60 decrease in the LDL- to HDL-cholesterol ratio represented a 16.2% reduction from baseline, with an estimated 10.2% potential reduction in coronary heart disease risk.^259^ A more recent meta-analysis of 40 clinical RCTs of AA monotherapy on lipid profile showed significantly improved total cholesterol, triglyceride, or HDL cholesterol in patients with higher baseline concentrations of total cholesterol and triglyceride, T2D, or those with low baseline plasma concentrations of AA.^260^ However, no significant effect on lipid profile was demonstrated among the overall trial population, which comprised healthy individuals with normal weight (12 trials) or those with cardiovascular disease (5 trials), chronic kidney disease (3 trials), hyperlipidemia (3 trials), hypertension/prehypertension (2 trials), T2D with overweight BMI (10 trials), or smokers (4 trials) (**Figure 10**).^260^ For context, in a meta-analysis of clinical RCTs of statin therapy for hypercholesterolemia, a decrease of 1 mmol/L in LDL cholesterol was associated with a 12% reduction in all-cause mortality, 17% reduction in cerebrovascular accident (CVA) and a 23% reduction in acute myocardial infarction (AMI).^260^ When applied to the Ashor et al^260^ study, the improvements in lipid profile with AA supplementation provided potential reductions of AMI (2.2%), CVA (1.6%), and all-cause mortality (all-cause mortality [1.2%]) in the overall cohort. The authors concluded that a personalized approach to vitamin C interventions for the prevention of cardiovascular disease may be beneficial in individuals with hypovitaminosis C or T2D, but further studies were required.

### Randomized Controlled Trials of Combined Antioxidant Therapy in Human Obesity

The effect of combined antioxidants in human obesity has also been studied. An RCT of combined nutraceutical-containing bergamot extract (120 mg flavonoids), phytosterols, AA, and chlorogenic acid from dried artichoke extract significantly improved triglyceride, LDL cholesterol, and HOMA-IR in overweight human participants compared with baseline and placebo treatment after an 8-week treatment period.^261^ Daily supplementation with 500 mg of AA, 400 IU of vitamin E, and 50 μg of selenium for 4 months improved these antioxidant concentrations and also oxidative stress markers (8-iso-prostaglandin F2α), but not markers of systemic inflammation (CRP) in overweight and obese children.^262^ Oxidative stress can be exacerbated post-exercise in patients with obesity. Daily supplementation of 500 mg of AA, 800 IU of vitamin E, and 10 mg of beta-carotene effectively reduced oxidative stress and improved inflammatory markers in obese patients after 30 minutes of cycle exercise.^263^ However, high-dose AA supplementation immediately after strenuous exercise in athletes with normal plasma ascorbate may be counterproductive, as it can inhibit beneficial exercise-induced hormesis.^264^

Negative results from clinical RCTs of combined micronutrients are also reported. An RCT involving 70 overweight adolescents (13–18 y) showed no significant improvement in metabolic dysfunction by anti-inflammatory nutrition supplement (containing 1000 mg eicosapentaenoic acid, 1000 mg docosahexaenoic acid, 567 mg AA, 390 mg α-tocopherol, 416 mg green tea extract, and 16.5 mg lycopene).^265^ In the SUpplementation en VItamines et Minéraux AntioXydants (SU.VI.MAX) primary prevention RCT, long-term daily supplementation with 120 mg of AA, 30 mg of vitamin E, 6 mg of beta-carotene, 20 mg of zinc, and 100 μg selenium over a 7.2-year period did not prevent the development of MetS compared with placebo in 5220 human adults who were generally healthy and well nourished at baseline. However, higher baseline serum beta-carotene and AA concentrations showed consistent significant negative associations with incident MetS. In addition, the 263 patients who developed MetS during the study were significantly more likely to have unhealthy lifestyles and higher BMI (29.6 ± 4.3 kg/m^2^), be smokers, and engage in lower physical activity at the end of the trial period.^266^ Proposed factors influencing the effectiveness of AA treatment for metabolic dysfunction include baseline metabolic phenotype, baseline dietary consumption, anthropometry, biochemistry, and gut microbiota.^265^ Patients with higher LDL and higher insulin resistance are more likely to respond to AA-containing treatment for insulin resistance.^265^ Some high-risk patients with existing obesity, MetS, elevated serum CRP, and baseline AA deficiency may not achieve adequate AA plasma concentrations despite long-term AA and micronutrient supplementation in RCTs^114^^,^^255^ (**Table 1**).

### Ascorbic Acid and Hypertension

Arterial hypertension is 1 of the 5 components of MetS in obesity.^267^^,^^268^ Endothelial dysfunction and hypertension during obesity are multifactorial, but closely related to hyperglycemia, exosome release from hypertrophic adipocytes, OSA, endothelial senescence, oxidative stress, ox-LDL, and impaired gastric or endothelial NO synthesis.^269^^,^^270^ Nutritional risks for hypertension during obesity include poor AA ingestion; high-fat, ultra-processed inflammatory Western diets; inadequate consumption of nitrate (NO_3_)-containing vegetables; and proton pump inhibitor (PPI) therapy.^245^^,^^271–273^ Foods that are rich in nitrates include dark-green leafy vegetables (such as spinach, kale, and romaine lettuce), beets, and celery; these also tend to be relatively high in AA. Adequate dietary nitrates (300–400 mg/d) are rapidly absorbed in the upper small intestine and circulate in the blood, 25% of which are excreted in saliva: the enterosalivary circulation. These salivary nitrates are then converted to nitrites (NO_2_; ∼10–20 mg/d) by nitrate-reducing bacteria in the mouth. Under conditions of normal gastric microbiota, gastric HCl acidity (pH <4), and gastric lumenal ascorbate concentrations, these swallowed nitrites are converted to nitrous acid (HNO_2_) and then to NO in the stomach, with the preferential formation of *S*-nitrosothiols instead of N-nitrosamines.^245^^,^^271–273^  *S*-nitrosothiols are readily absorbed and act as NO donors in the vascular circulation. Poor dietary AA intake as well as impaired gastric mucosal secretion of ascorbate contribute to a decreased gastric juice AA to nitrite ratio and failure of gastric NO formation. This is also related to gastric achlorhydria, *Helicobacter pylori* gastritis, gastric dysbiosis, PPI therapy, or ingested cigarette smoke–derived salivary thiocyanates, which favors nitrite persistence in the stomach and intragastric formation of carcinogenic N-nitroso compounds (NOCs).^245^^,^^271–273^

**Figure 11.**
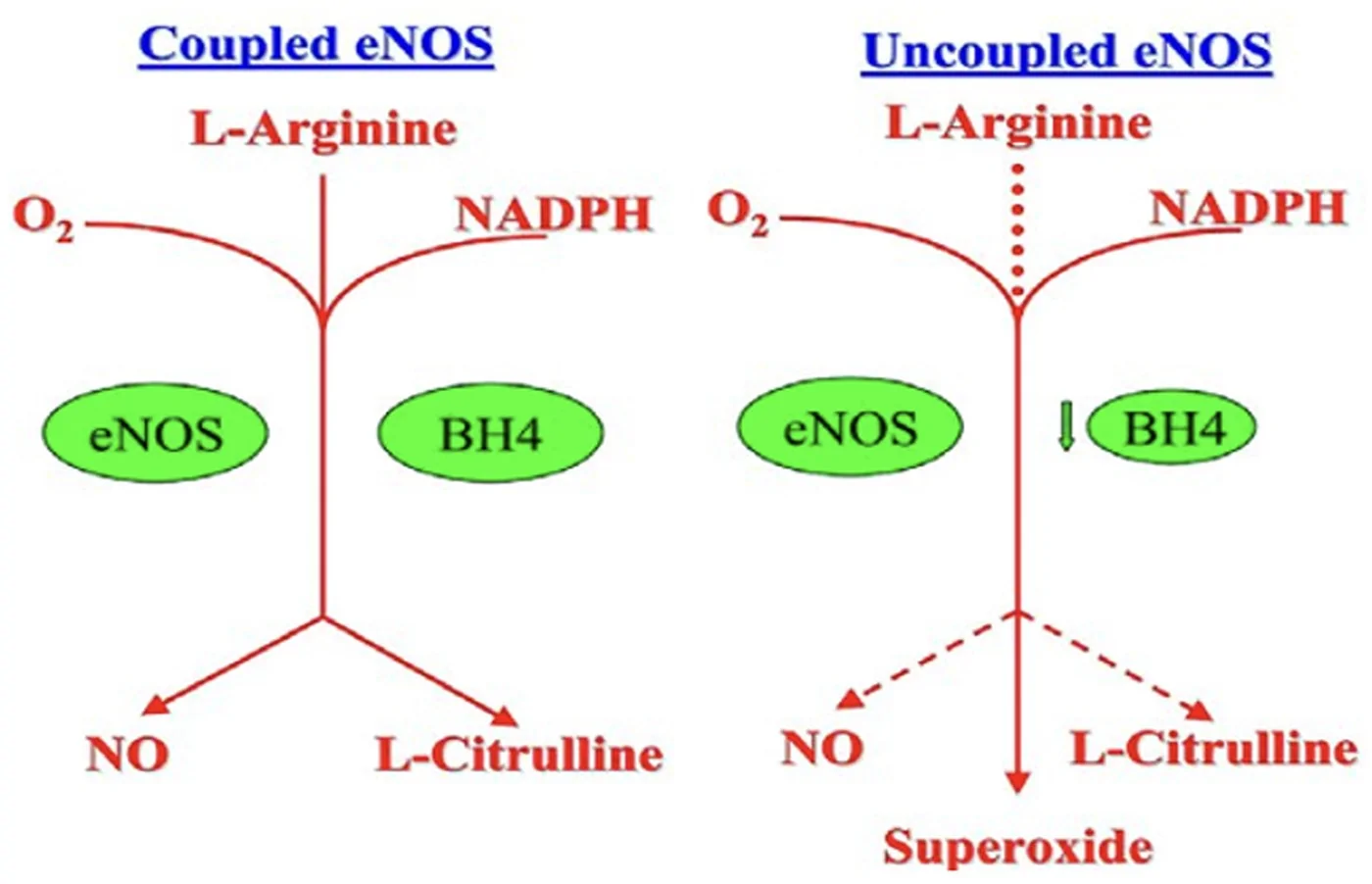
Coupled and Uncoupled Endothelial Nitric Oxide Synthase. (A) Coupled eNOS utilizes O_2_, l-arginine, and NAD(P)H to produce nitric oxide (NO) and l-citrulline. (B) eNOS can be uncoupled by endothelial tetrahydrobiopterin (BH4) deficiency or ascorbate deficiency to produce superoxide rather than NO, which may further reduce available NO by combining with it to form peroxynitrite. Abbreviations: eNOS, endothelial nitric oxide synthase; NAD(P)H, nicotinamide adenine dinucleotide (phosphate), reduced form; NO, nitric oxide. Reproduced from Rahangdale et al.^178^ with permission.

Plasma ascorbate also prevents uncoupling of endothelial tetrahydrobiopterin (BH4) and eNOS. Uncoupled eNOS results in endothelial superoxide generation, oxidized LDL, impaired NO production, vascular smooth muscle contraction, asymmetric dimethylarginine (ADMA) release, arterial hypertension, and accelerated atherosclerosis.^108^^,^^274–277^ Superoxide reacts with NO to form peroxynitrite, which further reduces available NO concentrations. Endothelial NOS also diminishes with aging, and thus dietary intake of nitrates and AA from vegetable and fruit consumption represents a valuable “default” source of NO for preventing progressive endothelial dysfunction and arterial hypertension^108^^,^^274–277^ (**Figure 11**).

A recent meta-analysis of 20 RCTs of AA monotherapy using a median oral dose of 758 mg/day for 6 weeks in adults found a modest reduction overall in systolic blood pressure of -3.0 mm Hg (95% CI, -4.7 to -1.3 mm Hg; *P* = .001).^278^ The effect on systolic blood pressure was more pronounced in patients with T2D (-4.6 mm Hg; 95% CI, -8.9 to -0.3 mm Hg; *P* = .03) and pre-existing hypertension (-3.2 mm Hg; 95% CI, -5.2 to -1.2 mm Hg; *P* = .002). This more pronounced effect of AA in patients with T2D was also reported in RCTs of AA monotherapy, with an SRMA of 28 RCTs involving 1574 patients with T2D finding a reduction in systolic blood pressure (mean difference, -6.27; 95% CI, -9.60 to -2.96 mm Hg; *P *= .0002) and diastolic blood pressure (-3.77; 95% CI, -6.13 to -1.42 mm Hg; *P *= .002)^89^^,^^115^^,^^116^ (**Table 1**). A meta-analysis of 18 prospective studies (451 291 participants, 145 492 hypertension cases) of high fruit and vegetable consumption compared with low consumption and the relative risk of hypertension was published in 2023.^279^ It found that high fruit and vegetable consumption of 880 g/day (RR = 0.90; 95% CI, 0.85–0.95) and high fruit consumption of 550 g/day (RR = 0.91; 95% CI, 0.85–0.98), but not vegetable consumption alone (RR = 0.97; 95% CI, 0.93–1.02), were associated with a significantly lowered risk of hypertension. For context, a recent metanalysis of 48 RCTs by the Blood Pressure Lowering Treatment Triallists’ Collaboration involving pharmacological antihypertensive interventions found that a reduction in systolic blood pressure of 5 mm Hg decreased both the primary and secondary risk of future major cardiovascular events (MACEs) in patients by 10%.^280^ However, most clinical intervention trials involving AA supplementation have not shown a benefit in the primary or secondary prevention of MACEs and the progression of atherosclerosis.^281–286^

One of the rare presentations of advanced scurvy is that of right-heart failure and severe pulmonary hypertension, which is rapidly reversible with AA administration but will progress to cardiogenic shock and cardiac arrest without prompt recognition and treatment.^287^ This is an example of the importance of AA in maintaining endothelial function and preventing uncoupling of BH4 from eNOS and the development of pulmonary vasoconstriction^178^ (**Figure 11**).

Patients with congestive heart failure (CHF) often have plasma AA deficiency, which is not explained by dietary intake alone. The effect of AA administration on cardiac function was examined in a meta-analysis of 6 clinical trials involving 246 cardiac patients.^288^^,^^289^ In the meta-analysis, mean left ventricular ejection fraction (LVEF) improved by 12.0% (95% CI, 8.1%–15.9%; *P *< .001) after either oral or intravenous AA administration. The effect was greater in patients with poorer LVEF and was not affected by AA dose. Suggested mechanisms include improved myocardial contractility and mechanical efficiency, endothelial function and baroreflex sensitivity, cardiac perfusion after percutaneous coronary intervention (PCI), diastolic function in an exercise test, and systolic myocardial velocity during hyperoxia, as well as reduced blood pressure, atrial fibrillation, HIF-1α, and oxidative stress.^288^^,^^289^

The severity of CHF correlates with higher levels of oxidative stress, lipid peroxidation (8,12-isoprostane F[2ɑ]-VI [8,12-iso-IPF2α-VI]), and lower plasma AA concentrations.^290^ In a study of patients with New York Heart Association (NYHA) severity II and III CHF, plasma 8,12-iso-IPF2α-VI levels were higher by more than 40% in patients with severe CHF (NYHA class III) than in patients with moderate disease (NYHA class II) (*P *= .0012). In a cumulative analysis of the entire cohort, there was a significant inverse association between LVEF, plasma 8,12-iso-IPF2α-VI levels, and plasma AA concentrations, but also with lipophilic antioxidants including vitamin E, vitamin A, lutein, and lycopene.^290^ This suggests that the lower concentrations of plasma AA in patients with heart failure or cardiovascular disease are related to antioxidant depletion by oxidative stress, lipid peroxidation, and endothelial dysfunction, rather than solely related to inadequate dietary intake.

## DIETARY INTERVENTIONS AND OBESITY

The obesogenic Western diet is characterized by overconsumption of saturated fats, highly processed grains, and sucrose/fructose-sweetened food and beverages, with a lack of fruit and vegetables, micronutrients, and dietary fiber.^28^^,^^291^ Adequate micronutrient consumption is dependent on a balanced and diverse diet. A recent cross-sectional study involving 41 021 adults from the US NHANES 2005–2018 data found that a high level of 11 dietary antioxidants and trace metals (AA, vitamin E, retinol, vitamin A, α-carotene, β-carotene, β-cryptoxanthin, iron, zinc, selenium, and copper) is related to a lower prevalence of obesity and abdominal obesity.^267^ The 2 micronutrients with the greatest inverse effect were iron and AA. On threshold effect analysis, the critical inflection point in this relationship for AA occurred at a similar daily intake of 43 mg for overall obesity and abdominal obesity.^267^

Several dietary interventions have been proposed for the management of obesity. A low-fat diet consists of less than 25% of energy from fat. A low-carbohydrate diet usually refers to a diet with carbohydrates that account for less than 45% of dietary caloric intake. A meta-analysis comparing a low-fat diet with other dietary interventions suggested no evidence of a superior effect on long-term weight loss by low-fat diet intervention.^292^ Another meta-analysis suggested a more significant decrease in body weight (weighted mean difference [WMD] = -2.2 kg; 95% CI, -3.4 to -1.0) and triglycerides (WMD = -0.3 mmol/L; 95% CI, -0.4 to -0.2), a greater increase in HDL cholesterol (WMD = +0.14 mmol/L; 95% CI, 0.1 to 0.2) but also increased LDL cholesterol (WMD = +0.2 mmol/L; 95% CI, 0.0 to 0.3) with a low-carbohydrate diet compared with a low-fat diet intervention.^293^

### Mediterranean Diet

The Mediterranean diet is rich in fruits and vegetables, nuts, seeds, seafoods, and olive oil. As such, it has a high content of antioxidants, polyphenols, fermentable fiber, and micronutrients and a lower glycemic load.^294^ A meta-analysis of clinical RCTs involving the Mediterranean dietary intervention showed improved body weight (mean difference [MD] = -1.8 kg; 95% CI, -2.9 to -0.6 kg) and BMI (MD = -0.6 kg/m^2^; 95% CI, -0.9 to -0.2 kg/m^2^), despite the relatively high dietary fat content. The meta-analysis also showed that the Mediterranean diet was associated with significant weight loss when combined with energy restriction (MD = -3.9 kg; 95% CI, -6.5 to -1.2 kg) and increased physical activity (MD = -4.0 kg; 95% CI, -5.8 to -2.2 kg) compared with the Mediterranean diet alone.^295^

### Dietary Approaches to Stop Hypertension Diet

The Dietary Approaches to Stop Hypertension (DASH) diet includes an increased intake of fruits and vegetables, whole grains, low-fat dairy, low-fat poultry meat, and seafood, and a reduced intake of cholesterol and fats.^296^ The effectiveness of the DASH diet was examined in a meta-analysis of 13 pooled RCTs involving patients with obesity/overweight, hypertension, or heart failure, which found a significant decrease in weight (WMD = -1.4 kg; 95% CI, -2.0 to -0.8) in 8–24 weeks, BMI (WMD = -0.4 kg/m^2^; 95% CI, -0.6 to -0.2) in 8–52 weeks, and waist circumference (WMD = -1.0 cm; 95% CI, -1.6 to -0.5 cm) in 24 weeks in participants consuming a DASH diet. The reported effect was greater in overweight or obese participants: weight (-1.6 kg; 95% CI, -2.2 to -1.0) and BMI (-0.5 kg/m^2^; 95% CI, -0.8, -0.2).^297^

### Dietary Intervention Plus AA Supplementation in Human Obesity

There are limited studies evaluating the combination of AA supplementation with dietary interventions in the management of obesity. A 1-year lifestyle RCT intervention with combined physical exercise (45 min/d aerobic physical exercise) and dietary changes (a hypocaloric diet consisting of carbohydrate [50%–60%], fat [23%–30%], and protein [15%–20%]) effectively induced weight loss and improvement in liver enzymes and markers of insulin resistance in children with NAFLD.^298^ The addition of AA and α-tocopherol to the lifestyle intervention did not increase the efficacy of the treatment.^298^ An RCT involving 81 overweight participants with MetS demonstrated that the incorporation of low-sodium vegetable juice into the DASH diet could achieve greater weight loss, but the effect was modest.^299^ A combination of 100% orange juice (OJ) with nutritional supervision and a balanced diet (Brazilian Food Guide) was investigated in another RCT involving 72 patients with MetS.^300^ Baseline and post-intervention antioxidant capacity was measured, but not plasma ascorbate concentration. Both groups who consumed a balanced diet (BD) over 12 weeks showed a mean reduction in glucose (-3%), total cholesterol (-8%), HDL cholesterol (-8%), BMI (-2%), waist circumference (-6%), and respective systolic and diastolic blood pressure (-8% and -10%). However, the addition of OJ (500 mL/d; containing 189 mg AA) to the balanced isocaloric diet decreased insulin (-9%; *P *= .17), HOMA-IR (-8%; *P *= .02), LDL cholesterol (-4%; *P *= .49), CRP (-28%; *P *= .05), and high-sensitivity CRP (hsCRP) concentrations of 1.0 mg/dL or greater (-61%; *P* < .001). The group who received OJ increased their respective AA intake by 133% compared with the control group. The authors postulated that the improvement in glycemic control and dyslipidemia could also be related to soluble fiber or citrus flavanones present in 500 mL of 100% OJ, including 121.6 mg hesperidin and 26.6 mg naringin. Hesperidin can regenerate ascorbate oxidized by ROS. Both intervention groups showed similar reversion (36% BD; 39% OJ+BD) of MetS towards normal (defined as <3 risk factors for MetS); however, the OJ+BD mitigated more MetS risk factors than the BD alone.^300^

## LIMITATIONS OF AA INTERVENTIONS AND FUTURE DIRECTIONS

There is evidence of the beneficial effect of dietary intervention in the management of obesity including the Mediterranean and DASH diets.^293–297^ The efficacy of synthetic AA supplementation in humans as monotherapy for obesity can be more modest when compared with comprehensive dietary interventions.^298^^,^^300^ This may be related to other phytonutrients and phytochemicals found in whole foods in addition to AA that provide beneficial or synergistic effects in oxidative stress, energy metabolism, metaflammation, and MetS in obesity. These include the following—vitamin B_9_ (folate), vitamin B_12_, vitamin A, and vitamin E (α-tocopherol); carotenoids; prebiotic fiber (resistant starch, inulin, rice bran); trace metals (zinc); and polyphenols, such as flavonoids, phloroglucinols, quinones, stilbenes, lignans, phenylpropanoids, and diarylheptanoids.^261^^,^^267^

There are associations between hypovitaminosis C and body weight/waist circumference/MetS, with several plausible biological mechanisms. However, causality has not been proven.^111^^,^^181^ Oral AA monotherapy is not sufficient to reverse established obesity, and the importance of both significant and clinically relevant improvements in the features of MetS is emphasized.^301^^,^^302^ Studies of AA supplementation in humans often do not differentiate between participants with baseline AA deficiency or those who are replete, which may dilute the results of the intervention. Low AA intake may be a surrogate marker for an unhealthy diet with inadequate fruit and vegetable consumption, rather than an independent risk factor for obesity and MetS. Extrapolating results from highly controlled animal models of obesity to humans, or the use of animal models with intact GLO function, may not be valid.^112^^,^^303^^,^^304^ Human RCTs of AA supplementation are often limited by small cohort size, with heterogeneity in methodological design (dose and duration of AA supplementation, combination treatments), and population characteristics (age, gender, BMI and comorbidities, T2D, study duration).^75^

Incretin therapy in obesity using GLP-1R agonists has been successful in terms of weight loss and resolution of MetS.^305–307^ However, the 16%–39% reduction in caloric consumption with glucagon-like peptide-1 receptor agonist (GLP-1RA) therapy is also associated with inadequate intake of iron, calcium, magnesium, zinc, and vitamins A, D, E, K, B_1_, B_12_, and C, which can exacerbate pre-existing deficiencies from ultra-processed diets.^307^ As such, there is still an important role for medical nutrition therapy during weight loss to prevent loss of lean mass, sarcopenia, osteopenia, vitamin deficiencies, and malnutrition during GLP-1RA therapy, and weight regain after cessation. This was exemplified in the Semaglutide Treatment Effect in People with Obesity 1 (STEP 1) Trial Extension study, which showed that mean total body weight loss ± SD was 17.3% ± 9.3% in the subcutaneous semaglutide 2.4 mg/week group after 68 weeks of treatment. However, at 12 months after cessation of semaglutide treatment, registered dietitian nutrition support, and lifestyle interventions, participants had regained 11.6% of total body weight with a return of features of MetS.^305–307^ Both a macronutrient and micronutrient dietary intervention is important in the prevention of weight recidivism after bariatric surgery^171^ or in GLP-1RA management of obesity.^307^

## CONCLUSION

Vitamin C plays an important role in the maintenance of healthy weight and energy metabolism, due to its beneficial effects on dioxygenase, mono-oxygenase, and mixed function oxygenase enzyme activity; oxidative stress and free radical scavenging; fatty acid transport and β-oxidation; cholesterol excretion, triglyceride metabolism, and hepatic steatosis; white adipose tissue inflammation and visceral adipose tissue dysfunction, cytokine release, and adipokine secretion; lipid peroxidation, endothelial dysfunction, and hypertension; and insulin sensitivity and fructose and glucose metabolism. Inadequate vitamin C intake and low plasma ascorbate concentrations correlate with BMI, fat mass, waist-hip ratio, and features of MetS, although causation has not been proven. This association may be a surrogate marker of an unhealthy lifestyle and a diet lacking fresh fruit and vegetables. People with obesity often have inadequate micronutrient intakes, and also require higher doses of vitamin C for extended periods to achieve adequate tissue saturation and protection against oxidative stress and systemic inflammation. The existing in vitro and in vivo preclinical data demonstrate the effectiveness of AA as both a prophylactic and a therapeutic intervention for obesity and MetS. The outcomes in human intervention studies are more modest and with noted limitations; but nevertheless, AA supplementation has shown clinically relevant improvements in lipid profile, hypertension, HbA1c, and hepatic steatosis. The addition of vitamin C to dietary interventions and increased physical activity may improve the efficacy of treatments for obesity and metabolic dysfunction. However, more data are required to understand the synergism between vitamin C supplementation, plant-based whole-food diets, and adequate exercise in weight control and MetS management. In addition, global harmonization of disparate international, national, and societal guidelines for AA RDA and SDT is required, particularly for high-risk individuals in the population including those with obesity and MetS.

## Data Availability

No datasets were generated or analyzed during the current study.

## References

[1] World Health Organization. Obesity and overweight. Accessed January 1, 2025. https://www.who.int/news-room/fact-sheets/detail/obesity-and-overweight

[2] Fryar CD , Carroll MD, Afful J. Prevalence of overweight, obesity, and severe obesity among adults aged 20 and over: United States, 1960–1962 through 2017–2018. NCHS Health E-Stats. 2020. Accessed January 1, 2025. https://www.cdc.gov/nchs/data/hestat/obesity-adult-17-18/obesity-adult.htm#Citation

[3] Waddell IS , Orfila C. Dietary fiber in the prevention of obesity and obesity-related chronic diseases: from epidemiological evidence to potential molecular mechanisms. Crit Rev Food Sci Nutr. 2023;63:8752-8767. 10.1080/10408398.2022.2061909 35471164

[4] GBD 2019 Risk Factors Collaborators. Global burden of 87 risk factors in 204 countries and territories, 1990-2019: a systematic analysis for the Global Burden of Disease Study 2019. Lancet. 2020;396:1223-1249. 10.1016/s0140-6736(20)30752-2 33069327 PMC7566194

[5] Okunogbe A , Nugent R, Spencer G, Powis J, Ralston J, Wilding J. Economic impacts of overweight and obesity: current and future estimates for 161 countries. BMJ Glob Health. 2022;7:e009773. 10.1136/bmjgh-2022-009773 PMC9494015 36130777

[6] He F , Liu J, Huang Y, et al Nutritional load in post-prandial oxidative stress and the pathogeneses of diabetes mellitus. NPJ Sci Food. 2024;8:41. 10.1038/s41538-024-00282-x 38937488 PMC11211471

[7] Clemente-Suárez VJ , Beltrán-Velasco AI, Redondo-Flórez L, Martín-Rodríguez A, Tornero-Aguilera JF. Global impacts of Western diet and its effects on metabolism and health: a narrative review. Nutrients. 2023;15:2749. 10.3390/nu15122749 PMC10302286 37375654

[8] Chen L , Chen XW, Huang X, Song BL, Wang Y, Wang Y. Regulation of glucose and lipid metabolism in health and disease. Sci China Life Sci. 2019;62:1420-1458. 10.1007/s11427-019-1563-3 31686320

[9] Lykkesfeldt J , Carr AC. Vitamin C—a scoping review for Nordic Nutrition Recommendations 2023. Food Nutr Res. 2023;67:67. 10.29219/fnr.v67.10300 PMC10770653 38187788

[10] Wilson R , Willis J, Gearry R, et al Inadequate vitamin C status in prediabetes and type 2 diabetes mellitus: associations with glycaemic control, obesity, and smoking. Nutrients. 2017;9:997. 10.3390/nu9090997 PMC5622757 28891932

[11] Ebenuwa I , Violet PC, Padayatty S, et al Abnormal urinary loss of vitamin C in diabetes: prevalence and clinical characteristics of a vitamin C renal leak. Am J Clin Nutr. 2022;116:274-284. 10.1093/ajcn/nqac063 35537862 PMC9257470

[12] Carr AC , Spencer E, Heenan H, Lunt H, Vollebregt M, Prickett TCR. Vitamin C status in people with types 1 and 2 diabetes mellitus and varying degrees of renal dysfunction: relationship to body weight. Antioxidants (Basel). 2022;11:245. 10.3390/antiox11020245 PMC8868094 35204128

[13] Carr AC , Lunt H, Wareham NJ, Myint PK. Estimating vitamin C intake requirements in diabetes mellitus: analysis of NHANES 2017-2018 and EPIC-Norfolk cohorts. Antioxidants (Basel). 2023;12:1863. 10.3390/antiox12101863 PMC10604478 37891943

[14] Yin J , Du L, Sheng C, You H, Wang X, Qu S. Vitamin C status and its change in relation to glucose-lipid metabolism in overweight and obesity patients following laparoscopic sleeve gastrectomy. Eur J Clin Nutr. 2022;76:1387-1392. 10.1038/s41430-022-01134-1 35422089

[15] Sun H , Karp J, Sun KM, Weaver CM. Decreasing vitamin C intake, low serum vitamin C level and risk for US adults with diabetes. Nutrients. 2022;14:3902. 10.3390/nu14193902 PMC9573084 36235555

[16] Park S , Kim K, Lee BK, Ahn J. A Healthy diet rich in calcium and vitamin C is inversely associated with metabolic syndrome risk in Korean adults from the KNHANES 2013-2017. Nutrients. 2021;13:1312. 10.3390/nu13041312 PMC8073625 33923450

[17] Bird JK , Feskens EJM, Melse-Boonstra A. A systematized review of the relationship between obesity and vitamin C requirements. Curr Dev Nutr. 2024;8:102152. 10.1016/j.cdnut.2024.102152 38666038 PMC11039309

[18] Rowe S , Carr AC. Global vitamin C status and prevalence of deficiency: a cause for concern? Nutrients. 2020;12:2008. 10.3390/nu12072008 PMC7400810 32640674

[19] Ravindran P , Wiltshire S, Das K, Wilson RB. Vitamin C deficiency in an Australian cohort of metropolitan surgical patients. Pathology. 2018;50:654-658. 10.1016/j.pathol.2018.07.004 30177219

[20] Golder JE , Bauer JD, Barker LA, Lemoh CN, Gibson SJ, Davidson ZE. Prevalence, risk factors, and clinical outcomes of vitamin C deficiency in adult hospitalized patients in high-income countries: a scoping review. Nutr Rev. 2024;82:1605-1621. 10.1093/nutrit/nuad157 38219216 PMC11465154

[21] Robitaille L , Hoffer LJ. A simple method for plasma total vitamin C analysis suitable for routine clinical laboratory use. Nutr J. 2016;15:40. 10.1186/s12937-016-0158-9 27102999 PMC4839128

[22] Langlois K , Cooper M, Colapinto CK. Vitamin C status of Canadian adults: findings from the 2012/2013 Canadian Health Measures Survey. Health Rep. 2016;27:3-10. 27192205

[23] Gan R , Eintracht S, Hoffer LJ. Vitamin C deficiency in a university teaching hospital. J Am Coll Nutr. 2008;27:428-433. 10.1080/07315724.2008.10719721 18838532

[24] Hoffer LJ. Re: “Vitamin C deficiency in a population of young Canadian adults.” Am J Epidemiol. 2010;171:387; author reply 387-8. 10.1093/aje/kwp400 19969531 PMC2878111

[25] Bhattacharyya P , Schemann K, Min SS, Sullivan DR, Fuller SJ. Serum vitamin C status of people in New South Wales: retrospective analysis of findings at a public referral hospital. Med J Aust. 2023;219:475-481. 10.5694/mja2.52132 37875282

[26] Powers CD , Sternberg MR, Patel SB, Pfeiffer CM, Storandt RJ, Schleicher RL. Vitamin C status of US adults assessed as part of the National Health and Nutrition Examination Survey remained unchanged between 2003-2006 and 2017-2018. J Appl Lab Med. 2023;8:272-284. 10.1093/jalm/jfac093 36592081 PMC10321475

[27] Zhu L , Spence C, Yang JW, Ma GX. The IDF definition is better suited for screening metabolic syndrome and estimating risks of diabetes in Asian American adults: evidence from NHANES 2011-2016. J Clin Med. 2020;9:3871. 10.3390/jcm9123871 PMC7759813 33260754

[28] Lathigara D , Kaushal D, Wilson RB. Molecular mechanisms of Western diet-induced obesity and obesity-related carcinogenesis—a narrative review. Metabolites. 2023;13:675. 10.3390/metabo13050675 PMC10222258 37233716

[29] Keshawarz A , Joehanes R, Ma J, et al Dietary and supplemental intake of vitamins C and E is associated with altered DNA methylation in an epigenome-wide association study meta-analysis. Epigenetics. 2023;18:2211361. 10.1080/15592294.2023.2211361 37233989 PMC10228397

[30] Patel P , Selvaraju V, Babu JR, Wang X, Geetha T. Racial disparities in methylation of NRF1, FTO, and LEPR gene in childhood obesity. Genes (Basel). 2022;13:2030. 10.3390/genes13112030 PMC9690504 36360268

[31] McAllan L , Baranasic D, Villicaña S, et al Integrative genomic analyses in adipocytes implicate DNA methylation in human obesity and diabetes. Nat Commun. 2023;14:2784. 10.1038/s41467-023-38439-z 37188674 PMC10185556

[32] Mahmoud AM. An overview of epigenetics in obesity: the role of lifestyle and therapeutic interventions. Int J Mol Sci. 2022;23:1341. 10.3390/ijms23031341 PMC8836029 35163268

[33] Li W , Chen D, Peng Y, Lu Z, Kwan M-P, Tse LA. Association between metabolic syndrome and mortality: prospective cohort study. JMIR Public Health Surveill. 2023;9:e44073. 10.2196/44073 37669100 PMC10509744

[34] Abdullah M , Jamil RT, Attia FN. Vitamin C (ascorbic acid). StatPearls. StatPearls Publishing; 2022.

[35] Doseděl M , Jirkovský E, Macáková K, et al Vitamin C—sources, physiological role, kinetics, deficiency, use, toxicity, and determination. Nutrients. 2021;13:615. 10.3390/nu13020615 PMC7918462 33668681

[36] Drouin G , Godin JR, Pagé B. The genetics of vitamin C loss in vertebrates. Curr Genomics. 2011;12:371-378. 10.2174/138920211796429736 22294879 PMC3145266

[37] Garcia-Diaz DF , Lopez-Legarrea P, Quintero P, Martinez JA. Vitamin C in the treatment and/or prevention of obesity. J Nutr Sci Vitaminol (Tokyo). 2014;60:367-379. 10.3177/jnsv.60.367 25866299

[38] Kietzmann T. Vitamin C: From nutrition to oxygen sensing and epigenetics. Redox Biol. 2023;63:102753. 10.1016/j.redox.2023.102753 37263060 PMC10245123

[39] Linster CL , Van Schaftingen E. Vitamin C, biosynthesis, recycling and degradation in mammals. FEBS J. 2007;274:1-22. 10.1111/j.1742-4658.2006.05607.x 17222174

[40] Naidu KA. Vitamin C in human health and disease is still a mystery? An overview. Nutr J. 2003;2:7. 10.1186/1475-2891-2-7 14498993 PMC201008

[41] Ronchetti IP , Quaglino D Jr, Bergamini G. Ascorbic acid and connective tissue. Subcell Biochem. 1996;25:249-264. 10.1007/978-1-4613-0325-1_13 8821978

[42] Sim M , Hong S, Jung S, et al Vitamin C supplementation promotes mental vitality in healthy young adults: results from a cross-sectional analysis and a randomized, double-blind, placebo-controlled trial. Eur J Nutr. 2022;61:447-459. 10.1007/s00394-021-02656-3 34476568 PMC8783887

[43] Lang P , Hasselwander S, Li H, Xia N. Effects of different diets used in diet-induced obesity models on insulin resistance and vascular dysfunction in C57BL/6 mice. Sci Rep. 2019;9:19556. 10.1038/s41598-019-55987-x 31862918 PMC6925252

[44] Lykkesfeldt J , Tveden-Nyborg P. The pharmacokinetics of vitamin C. Nutrients. 2019;11:2412. 10.3390/nu11102412 PMC6835439 31601028

[45] Levine M , Conry-Cantilena C, Wang Y, et al Vitamin C pharmacokinetics in healthy volunteers: evidence for a Recommended Dietary Allowance. Proc Natl Acad Sci USA. 1996;93:3704-3709. 10.1073/pnas.93.8.3704 8623000 PMC39676

[46] Padayatty SJ , Levine M. New insights into the physiology and pharmacology of vitamin C. CMAJ. 2001;164:353-355. 11232136 PMC80729

[47] Padayatty SJ , Levine M. Vitamin C: the known and the unknown and Goldilocks. Oral Dis. 2016;22:463-493. 10.1111/odi.12446 26808119 PMC4959991

[48] Holman GD. Structure, function and regulation of mammalian glucose transporters of the SLC2 family. Pflugers Arch. 2020;472:1155-1175. 10.1007/s00424-020-02411-3 32591905 PMC7462842

[49] Hemilä H , Chalker E. Abrupt termination of vitamin C from ICU patients may increase mortality: secondary analysis of the LOVIT trial. Eur J Clin Nutr. 2023;77:490-494. 10.1038/s41430-022-01254-8 36539454 PMC10115628

[50] Lunt H , Carr AC, Heenan HF, et al People with diabetes and hypovitaminosis C fail to conserve urinary vitamin C. J Clin Transl Endocrinol. 2023;31:100316. 10.1016/j.jcte.2023.100316 36873955 PMC9982671

[51] Hirsch IB , Atchley DH, Tsai E, Labbé RF, Chait A. Ascorbic acid clearance in diabetic nephropathy. J Diabetes Complications. 1998;12:259-263. 10.1016/s1056-8727(97)00125-6 9747642

[52] Carr AC , Lykkesfeldt J. Factors affecting the vitamin C dose-concentration relationship: implications for global vitamin C dietary recommendations. Nutrients. 2023;15:1657. 10.3390/nu15071657 PMC10096887 37049497

[53] Carr AC , Lykkesfeldt J. Discrepancies in global vitamin C recommendations: a review of RDA criteria and underlying health perspectives. Crit Rev Food Sci Nutr. 2021;61:742-755. 10.1080/10408398.2020.1744513 32223303

[54] Seghieri G , Martinoli L, Miceli M, et al Renal excretion of ascorbic acid in insulin dependent diabetes mellitus. Int J Vitam Nutr Res. 1994;64:119-124. 7960490

[55] Iwakawa H , Nakamura Y, Fukui T, et al Concentrations of water-soluble vitamins in blood and urinary excretion in patients with diabetes mellitus. Nutr Metab Insights. 2016;9:85-92. 10.4137/nmi.S40595 27812289 PMC5091094

[56] Hujoel PP , Hujoel MLA. Vitamin C and scar strength: analysis of a historical trial and implications for collagen-related pathologies. Am J Clin Nutr. 2022;115:8-17. 10.1093/ajcn/nqab262 34396385

[57] Uhl E. Ascorbic acid requirements of adults: 30 mg or 75 mg? Am J Clin Nutr. 1958;6:146-150. 10.1093/ajcn/6.2.146 13520645

[58] Hodges RE , Baker EM, Hood J, Sauberlich HE, March SC. Experimental scurvy in man. Am J Clin Nutr. 1969;22:535-548. 10.1093/ajcn/22.5.535 4977512

[59] National Research Council Subcommittee on the Tenth Edition of the Recommended Dietary A. *The National Academies Collection: Reports funded by National Institutes of Health*. *Recommended Dietary Allowances: 10th Edition*. National Academies Press; 1989.

[60] Pauling L. Are Recommended Daily Allowances for vitamin C adequate? Proc Natl Acad Sci USA. 1974;71:4442-4446. 10.1073/pnas.71.11.4442 4612519 PMC433902

[61] Carr AC , Rowe S. Factors affecting vitamin C status and prevalence of deficiency: a global health perspective. Nutrients. 2020;12:1963. 10.3390/nu12071963 PMC7400679 32630245

[62] Carr AC , Block G, Lykkesfeldt J. Estimation of vitamin C intake requirements based on body weight: implications for obesity. Nutrients. 2022;14:1460. 10.3390/nu14071460 PMC9003354 35406073

[63] Carr AC , Myint PK, Vijewardane SC, Johnstone AM, Crook J, Lykkesfeldt J. An increasing proportion of the population is not covered by the current RDA for vitamin C—interrogation of EPIC-Norfolk and NHANES 2017/2018 cohorts. Crit Rev Food Sci Nutr. 2025;65:3084-3095. 10.1080/10408398.2024.2356760 38845362

[64] National Health and Medical Research Council; Australian Government Department of Health and Ageing; New Zealand Ministry of Health. Nutrient Reference Values for Australia and New Zealand. Canberra, Australia: National Health and Medical Research Council; 2006.

[65] Institute of Medicine Panel on Dietary Antioxidants and Related Compounds. Dietary Reference Intakes for Vitamin C, Vitamin E, Selenium, and Carotenoids. US National Academies Press; 2000. 25077263

[66] EFSA NDA Panel (EFSA Panel on Dietetic Products Nutrition and Allergies). Scientific opinion on dietary reference values for vitamin C. EFSA J. 2013;11:3418. 10.2903/j.efsa.2013.3418

[67] Mazaheri-Tehrani S , Yazdi M, Heidari-Beni M, Yazdani Z, Kelishadi R. The association between vitamin C dietary intake and its serum levels with anthropometric indices: a systematic review and meta-analysis. Complement Ther Clin Pract. 2023;51:101733. 10.1016/j.ctcp.2023.101733 36774847

[68] Elste V , Troesch B, Eggersdorfer M, Weber P. Emerging evidence on neutrophil motility supporting its usefulness to define vitamin C intake requirements. Nutrients. 2017;9:503. 10.3390/nu9050503 PMC5452233 28509882

[69] Bozonet SM , Carr AC, Pullar JM, Vissers MC. Enhanced human neutrophil vitamin C status, chemotaxis and oxidant generation following dietary supplementation with vitamin C-rich SunGold kiwifruit. Nutrients. 2015;7:2574-2588. 10.3390/nu7042574 25912037 PMC4425162

[70] Garcia-Diaz DF , Campion J, Quintero P, Milagro FI, Moreno-Aliaga MJ, Martinez JA. Vitamin C modulates the interaction between adipocytes and macrophages. Molecular Nutr Food Res. 2011;55(Suppl 2):S257-S263. 10.1002/mnfr.201100296 21796779

[71] Yang J-P , Shin J-H, Seo S-H, Kim S-G, Lee SH, Shin E-H. Effects of antioxidants in reducing accumulation of fat in hepatocyte. Int J Mol Sci. 2018;19:2563. 30158441 10.3390/ijms19092563 PMC6164327

[72] Gu X , Luo X, Wang Y, et al Ascorbic acid attenuates cell stress by activating the fibroblast growth factor 21/fibroblast growth factor receptor 2/adiponectin pathway in HepG2 cells. Mol Med Rep. 2019;20:2450-2458. 10.3892/mmr.2019.10457 31322211

[73] Ojo OO , Leake DS. Vitamins E and C do not effectively inhibit low density lipoprotein oxidation by ferritin at lysosomal pH. Free Radic Res. 2021;55:525-534. 10.1080/10715762.2021.1964494 34396869

[74] Bo S , Ciccone G, Durazzo M, et al Efficacy of antioxidant treatment in reducing resistin serum levels: a randomized study. PLoS Clin Trials. 2007;2:e17. 10.1371/journal.pctr.0020017 17479165 PMC1865087

[75] Balbi ME , Tonin FS, Mendes AM, et al Antioxidant effects of vitamins in type 2 diabetes: a meta-analysis of randomized controlled trials. Diabetol Metab Syndr. 2018;10:18. 10.1186/s13098-018-0318-5 29568330 PMC5853104

[76] Otsuka M , Matsuzawa M, Ha TY, Arakawa N. Contribution of a high dose of L-ascorbic acid to carnitine synthesis in guinea pigs fed high-fat diets. J Nutr Sci Vitaminol (Tokyo). 1999;45:163-171. 10.3177/jnsv.45.163 10450557

[77] Johnston C , Solomon RE, Corte C. Vitamin C depletion is associated with alterations in blood histamine and plasma free carnitine in adults. J Am Coll Nutr. 1996;15:586-591. 8951736 10.1080/07315724.1996.10718634

[78] Johnston CS , Corte C, Swan PD. Marginal vitamin C status is associated with reduced fat oxidation during submaximal exercise in young adults. Nutr Metab. 2006;3:35. 10.1186/1743-7075-3-35 PMC1564400 16945143

[79] Cárcamo JM , Pedraza A, Bórquez-Ojeda O, Golde DW. Vitamin C suppresses TNF alpha-induced NF kappa B activation by inhibiting I kappa B alpha phosphorylation. Biochemistry. 2002;41:12995-13002. 10.1021/bi0263210 12390026

[80] Rose FJ , Webster J, Barry JB, Phillips LK, Richards AA, Whitehead JP. Synergistic effects of ascorbic acid and thiazolidinedione on secretion of high molecular weight adiponectin from human adipocytes. Diabetes Obes Metab. 2010;12:1084-1089. 10.1111/j.1463-1326.2010.01297.x 20977580

[81] Garcia-Diaz DF , Campion J, Milagro FI, Boque N, Moreno-Aliaga MJ, Martinez JA. Vitamin C inhibits leptin secretion and some glucose/lipid metabolic pathways in primary rat adipocytes. J Mol Endocrinol. 2010;45:33-43. 10.1677/jme-09-0160 20400526

[82] Luo X , Ng C, He J, et al Vitamin C protects against hypoxia, inflammation, and ER stress in primary human preadipocytes and adipocytes. Mol Cell Endocrinol. 2022;556:111740. 10.1016/j.mce.2022.111740 35932980

[83] del Mar Bibiloni M , Maffeis C, Llompart I, Pons A, Tur JA. Dietary factors associated with subclinical inflammation among girls. Eur J Clin Nutr. 2013;67:1264-1270. 10.1038/ejcn.2013.196 24149444

[84] Jamalan M , Rezazadeh M, Zeinali M, Ghaffari MA. Effect of ascorbic acid and alpha-tocopherol supplementations on serum leptin, tumor necrosis factor alpha, and serum amyloid A levels in individuals with type 2 diabetes mellitus. Avicenna J Phytomed. 2015;5:531-539. 26693410 PMC4678498

[85] He Z , Li X, Yang H, et al Effects of oral vitamin C supplementation on liver health and associated parameters in patients with non-alcoholic fatty liver disease: a randomized clinical trial. Clinical trial. Front Nutr. 2021;8:745609. 10.3389/fnut.2021.745609 34595203 PMC8478121

[86] Park Y , Jang J, Lee D, Yoon M. Vitamin C inhibits visceral adipocyte hypertrophy and lowers blood glucose levels in high-fat-diet-induced obese C57BL/6J mice. BSL. 2018;24:311-318. 10.15616/BSL.2018.24.4.311

[87] Jeon S , Lee J, Shin Y, Yoon M. Ascorbic acid reduces insulin resistance and pancreatic steatosis by regulating adipocyte hypertrophy in obese ovariectomized mice. Can J Physiol Pharmacol. 2023;101:294-303. 10.1139/cjpp-2022-0339 36999637

[88] Gillani SW , Sulaiman SAS, Abdul MIM, Baig MR. Combined effect of metformin with ascorbic acid versus acetyl salicylic acid on diabetes-related cardiovascular complication; a 12-month single blind multicenter randomized control trial. Cardiovasc Diabetol. 2017;16:103. 10.1186/s12933-017-0584-9 28807030 PMC5556597

[89] Mason SA , Keske MA, Wadley GD. Effects of vitamin C supplementation on glycemic control and cardiovascular risk factors in people with type 2 diabetes: a GRADE-assessed systematic review and meta-analysis of randomized controlled trials. Diabetes Care. 2021;44:618-630. 10.2337/dc20-1893 33472962

[90] Jenkins S. Hypovitaminosis C and cholelithiasis in guinea pigs. Biochem Biophys Res Commun. 1977;77:1030-1035. 901508 10.1016/s0006-291x(77)80081-8

[91] Gustafsson U , Wang F, Axelson M, Kallner A, Sahlin S, Einarsson K. The effect of vitamin C in high doses on plasma and biliary lipid composition in patients with cholesterol gallstones: prolongation of the nucleation time. Eur J Clin Invest. 1997;27:387-391. 10.1046/j.1365-2362.1997.1240670.x 9179545

[92] Wang D , Yang X, Chen Y, et al Ascorbic acid enhances low-density lipoprotein receptor expression by suppressing proprotein convertase subtilisin/kexin 9 expression. J Biol Chem. 2020;295:15870-15882. 10.1074/jbc.RA120.015623 32913121 PMC7681026

[93] Duane WC , Hutton SW. Lack of effect of experimental ascorbic acid deficiency on bile acid metabolism, sterol balance, and biliary lipid composition in man. J Lipid Res. 1983;24:1186-1195. 6631246

[94] Walcher T , Haenle MM, Kron M, et al; EMIL Study Group. Vitamin C supplement use may protect against gallstones: an observational study on a randomly selected population. BMC Gastroenterol. 2009;9:74. 10.1186/1471-230x-9-74 19814821 PMC2763865

[95] Chen Q , Zhao L, Mei L, et al Vitamin C and vitamin D(3) alleviate metabolic-associated fatty liver disease by regulating the gut microbiota and bile acid metabolism via the gut-liver axis. Front Pharmacol. 2023;14:1163694. 10.3389/fphar.2023.1163694 37089915 PMC10113476

[96] Smith-Cortinez N , Fagundes RR, Gomez V, et al Collagen release by human hepatic stellate cells requires vitamin C and is efficiently blocked by hydroxylase inhibition. FASEB J. 2021;35:e21219. 10.1096/fj.202001564RR 33236467 PMC12315489

[97] Wang D , Yin Z, Han L, et al Ascorbic acid inhibits transcriptional activities of LXRα to ameliorate lipid metabolism disorder. J Funct Foods. 2022;88:104901. 10.1016/j.jff.2021.104901

[98] Rezazadeh A , Yazdanparast R, Molaei M. Amelioration of diet-induced nonalcoholic steatohepatitis in rats by Mn-salen complexes via reduction of oxidative stress. J Biomed Sci. 2012;19:26. 10.1186/1423-0127-19-26 22375551 PMC3311616

[99] Lee SW , Lee YJ, Baek SM, et al Mega-dose vitamin C ameliorates nonalcoholic fatty liver disease in a mouse fast-food diet model. Nutrients. 2022;14:2195. 10.3390/nu14112195 PMC9182669 35683997

[100] Park SH , Han AL, Kim NH, Shin SR. Liver histological improvement after administration of high-dose vitamin C in guinea pig with nonalcoholic steatohepatitis. Int J Vitam Nutr Res. 2018;88:263-269. 10.1024/0300-9831/a000515 30789804

[101] Zeng Q , Zhao L, Meng C, et al Prophylactic and therapeutic effects of different doses of vitamin C on high-fat-diet-induced non-alcoholic fatty liver disease in mice. Biomed Pharmacother. 2020;131:110792. 10.1016/j.biopha.2020.110792 33152949

[102] Lee SW , Baek SM, Kang KK, et al Vitamin C deficiency inhibits nonalcoholic fatty liver disease progression through impaired de novo lipogenesis. Am J Pathol. 2021;191:1550-1563. 10.1016/j.ajpath.2021.05.020 34126083

[103] Skat-Rørdam J , Pedersen K, Skovsted GF, et al Vitamin C deficiency may delay diet-induced NASH regression in the guinea pig. Antioxidants (Basel). 2021;11:69. 10.3390/antiox11010069 PMC8772888 35052573

[104] Harrison SA , Torgerson S, Hayashi P, Ward J, Schenker S. Vitamin E and vitamin C treatment improves fibrosis in patients with nonalcoholic steatohepatitis. Am J Gastroenterol. 2003;98:2485-2490. 10.1016/j.amjgastroenterol.2003.08.005 14638353

[105] Da Silva HE , Arendt BM, Noureldin SA, Therapondos G, Guindi M, Allard JP. A cross-sectional study assessing dietary intake and physical activity in Canadian patients with nonalcoholic fatty liver disease vs healthy controls. J Acad Nutr Diet. 2014;114:1181-1194. 24631112 10.1016/j.jand.2014.01.009

[106] Barbakadze G , Khachidze T, Sulaberidze G, Burnadze K, Jebashvili M. Comparative analysis of efficiency of ursodeoxycholic acid combination of vitamin E and vitamin C in treatment of non-diabetic nonalcoholic steatohepatitis. Georgian Med News. 2019;288:81-85. 31101782

[107] Xie ZQ , Li HX, Tan WL, et al Association of serum vitamin C with NAFLD and MAFLD among adults in the United States. Front Nutr. 2021;8:795391. 10.3389/fnut.2021.795391 35187020 PMC8854786

[108] Naylor GJ , Grant L, Smith C. A double blind placebo controlled trial of ascorbic acid in obesity. Nutr Health. 1985;4:25-28. 10.1177/026010618500400104 3914623

[109] Paolisso G , Balbi V, Volpe C, et al Metabolic benefits deriving from chronic vitamin C supplementation in aged non-insulin dependent diabetics. J Am Coll Nutr. 1995;14:387-392. 10.1080/07315724.1995.10718526 8568117

[110] García OP , Ronquillo D, Caamaño M, Camacho M, Long KZ, Rosado JL. Zinc, vitamin A, and vitamin C status are associated with leptin concentrations and obesity in Mexican women: results from a cross-sectional study. Nutr Metab (Lond). 2012;9:59-59. 22703731 10.1186/1743-7075-9-59 PMC3406981

[111] Larsen SC , Ängquist L, Ahluwalia TS, et al Dietary ascorbic acid and subsequent change in body weight and waist circumference: associations may depend on genetic predisposition to obesity—a prospective study of three independent cohorts. Nutr J. 2014;13:43. 10.1186/1475-2891-13-43 24886192 PMC4024624

[112] Guo H , Ding J, Liu Q, Li Y, Liang J, Zhang Y. Vitamin C and metabolic syndrome: a meta-analysis of observational studies. Front Nutr. 2021;8:728880. 10.3389/fnut.2021.728880 34692744 PMC8531097

[113] Pei X , Yao J, Ran S, et al Association of serum water-soluble vitamin exposures with the risk of metabolic syndrome: results from NHANES 2003-2006. Front Endocrinol (Lausanne). 2023;14:1167317. 10.3389/fendo.2023.1167317 37251666 PMC10213561

[114] Vlasiuk E , Zawari M, Whitehead R, Williman J, Carr AC. A high vitamin C micronutrient supplement is unable to attenuate inflammation in people with metabolic syndrome but may improve metabolic health indices: a randomised controlled trial. Antioxidants (Basel). 2024;13:404. 10.3390/antiox13040404 PMC11047647 38671852

[115] Shateri Z , Keshavarz SA, Hosseini S, Chamari M, Hosseini M, Nasli E. Effect of vitamin C supplementation on blood pressure level in type 2 diabetes mellitus: a randomized, double-blind, placebo-controlled trial. Biosci Biotech Res Asia. 2016;13:279-286.

[116] Mason SA , Rasmussen B, van Loon LJC, Salmon J, Wadley GD. Ascorbic acid supplementation improves postprandial glycaemic control and blood pressure in individuals with type 2 diabetes: findings of a randomized cross-over trial. Diabetes Obes Metab. 2019;21:674-682. 10.1111/dom.13571 30394006

[117] Zhitkovich A. Nuclear and cytoplasmic functions of vitamin C. Chem Res Toxicol. 2020;33:2515-2526. 10.1021/acs.chemrestox.0c00348 33001635 PMC7572711

[118] Li R , Jia Z, Trush MA. Defining ROS in biology and medicine. React Oxyg Species (Apex). 2016;1:9-21. 10.20455/ros.2016.803 29707643 PMC5921829

[119] Fujii J , Homma T, Osaki T. Superoxide radicals in the execution of cell death. Antioxidants (Basel). 2022;11:501. 10.3390/antiox11030501 PMC8944419 35326151

[120] Figueroa-Méndez R , Rivas-Arancibia S. Vitamin C in health and disease: its role in the metabolism of cells and redox state in the brain. Front Physiol. 2015;6:397. 10.3389/fphys.2015.00397 26779027 PMC4688356

[121] Marseglia L , Manti S, D’Angelo G, et al Oxidative stress in obesity: a critical component in human diseases. Int J Mol Sci. 2014;16:378-400. 10.3390/ijms16010378 25548896 PMC4307252

[122] Han CY. Roles of reactive oxygen species on insulin resistance in adipose tissue. Diabetes Metab J. 2016;40:272-279. 10.4093/dmj.2016.40.4.272 27352152 PMC4995181

[123] Moghadam ZM , Henneke P, Kolter J. From flies to men: ROS and the NADPH oxidase in phagocytes. Front Cell Dev Biol. 2021;9:628991. 10.3389/fcell.2021.628991 33842458 PMC8033005

[124] Smith U , Li Q, Rydén M, Spalding KL. Cellular senescence and its role in white adipose tissue. Int J Obes (Lond). 2021;45:934-943. 10.1038/s41366-021-00757-x 33510393

[125] Vincent HK , Innes KE, Vincent KR. Oxidative stress and potential interventions to reduce oxidative stress in overweight and obesity. Diabetes Obes Metab. 2007;9:813-839. 10.1111/j.1463-1326.2007.00692.x 17924865

[126] Palmieri VO , Grattagliano I, Portincasa P, Palasciano G. Systemic oxidative alterations are associated with visceral adiposity and liver steatosis in patients with metabolic syndrome. J Nutr. 2006;136:3022-3026. 10.1093/jn/136.12.3022 17116714

[127] Kaźmierczak-Barańska J , Boguszewska K, Adamus-Grabicka A, Karwowski BT. Two faces of vitamin C-antioxidative and pro-oxidative agent. Nutrients. 2020;12:1501. 10.3390/nu12051501 PMC7285147 32455696

[128] Ivanova IP , Trofimova SV, Piskarev IM, Aristova NA, Burhina OE, Soshnikova OO. Mechanism of chemiluminescence in Fenton reaction. JBPC. 2012;03:88-100.

[129] Pullar JM , Carr AC, Vissers MCM. The roles of vitamin C in skin health. Nutrients. 2017;9:866. 10.3390/nu9080866 PMC5579659 28805671

[130] Buettner GR. The pecking order of free radicals and antioxidants: lipid peroxidation, alpha-tocopherol, and ascorbate. Arch Biochem Biophys. 1993;300:535-543. 10.1006/abbi.1993.1074 8434935

[131] Naziroğlu M , Simşek M, Simşek H, Aydilek N, Ozcan Z, Atilgan R. The effects of hormone replacement therapy combined with vitamins C and E on antioxidants levels and lipid profiles in postmenopausal women with type 2 diabetes. Clin Chim Acta. 2004;344:63-71. 10.1016/j.cccn.2004.01.031 15149872

[132] Argaev-Frenkel L , Rosenzweig T. Redox balance in type 2 diabetes: therapeutic potential and the challenge of antioxidant-based therapy. Antioxidants (Basel). 2023;12:994. 10.3390/antiox12050994 PMC10215840 37237860

[133] Konno T , Melo EP, Chambers JE, Avezov E. Intracellular sources of ROS/H(2)O(2) in health and neurodegeneration: spotlight on endoplasmic reticulum. Cells. 2021;10:233. 10.3390/cells10020233 PMC7912550 33504070

[134] Szarka A , Lőrincz T. The role of ascorbate in protein folding. Protoplasma. 2014;251:489-497. 10.1007/s00709-013-0560-5 24150425

[135] Zhu H , Itoh K, Yamamoto M, Zweier JL, Li Y. Role of Nrf2 signaling in regulation of antioxidants and phase 2 enzymes in cardiac fibroblasts: protection against reactive oxygen and nitrogen species-induced cell injury. FEBS Lett. 2005;579:3029-3036. 10.1016/j.febslet.2005.04.058 15896789

[136] Li S , Eguchi N, Lau H, Ichii H. The role of the Nrf2 signaling in obesity and insulin resistance. Int J Mol Sci. 2020;21:6973. 10.3390/ijms21186973 PMC7555440 32971975

[137] Ferrada L , Magdalena R, Barahona MJ, et al Two distinct faces of vitamin C: AA vs. DHA. Antioxidants (Basel). 2021;10:215. 10.3390/antiox10020215 PMC7912923 33535710

[138] Johnston CS , Barkyoumb GM, Schumacher SS. Vitamin C supplementation slightly improves physical activity levels and reduces cold incidence in men with marginal vitamin C status: a randomized controlled trial. Nutrients. 2014;6:2572-2583. 10.3390/nu6072572 25010554 PMC4113757

[139] Rebouche CJ. Kinetics, pharmacokinetics, and regulation of L-carnitine and acetyl-L-carnitine metabolism. Ann N Y Acad Sci. 2004;1033:30-41. 10.1196/annals.1320.003 15591001

[140] Houten SM , Wanders RJA, Ranea-Robles P. Metabolic interactions between peroxisomes and mitochondria with a special focus on acylcarnitine metabolism. Biochim Biophys Acta Mol Basis Dis. 2020;1866:165720. 10.1016/j.bbadis.2020.165720 32057943 PMC7146961

[141] Reda E , D’Iddio S, Nicolai R, Benatti P, Calvani M. The carnitine system and body composition. Acta Diabetol. 2003;40(Suppl 1):s106-s113. 10.1007/s00592-003-0040-z 14618447

[142] Hoppel C. The role of carnitine in normal and altered fatty acid metabolism. Am J Kidney Dis. 2003;41:S4-12. 10.1016/S0272-6386(03)00112-4 12751049

[143] Rebouche CJ. Ascorbic acid and carnitine biosynthesis. Am J Clin Nutr. 1991;54:1147s-1152s. 10.1093/ajcn/54.6.1147s 1962562

[144] Ha TY , Otsuka M, Arakawa N. Ascorbate indirectly stimulates fatty acid utilization in primary cultured guinea pig hepatocytes by enhancing carnitine synthesis. J Nutr. 1994;124:732-737. 8169666 10.1093/jn/124.5.732

[145] Pullar JM , Carr AC, Bozonet SM, Vissers MCM. High vitamin C status is associated with elevated mood in male tertiary students. Antioxidants (Basel). 2018;7:91. 10.3390/antiox7070091 PMC6071228 30012945

[146] Huck CJ , Johnston CS, Beezhold BL, Swan PD. Vitamin C status and perception of effort during exercise in obese adults adhering to a calorie-reduced diet. Nutrition. 2013;29:42-45. 10.1016/j.nut.2012.01.021 22677357

[147] Rebouche CJ. The ability of guinea pigs to synthesize carnitine at a normal rate from epsilon-N-trimethyllysine or gamma-butyrobetaine in vivo is not compromised by experimental vitamin C deficiency. Metabolism. 1995;44:624-629. 10.1016/0026-0495(95)90120-5 7752911

[148] Furusawa H , Sato Y, Tanaka Y, et al Vitamin C is not essential for carnitine biosynthesis in vivo: verification in vitamin C-depleted senescence marker protein-30/gluconolactonase knockout mice. Biol Pharm Bull. 2008;31:1673-1679. 10.1248/bpb.31.1673 18758058

[149] Assante G , Chandrasekaran S, Ng S, et al Acetyl-CoA metabolism drives epigenome change and contributes to carcinogenesis risk in fatty liver disease. Genome Med. 2022;14:67. 10.1186/s13073-022-01071-5 35739588 PMC9219160

[150] Dahlman I , Sinha I, Gao H, et al The fat cell epigenetic signature in post-obese women is characterized by global hypomethylation and differential DNA methylation of adipogenesis genes. Int J Obes (Lond). 2015;39:910-919. 10.1038/ijo.2015.31 25783037

[151] Fujiki K , Shinoda A, Kano F, Sato R, Shirahige K, Murata M. PPARγ-induced PARylation promotes local DNA demethylation by production of 5-hydroxymethylcytosine. Nat Commun. 2013;4:2262. 10.1038/ncomms3262 23912449

[152] Yuan Y , Liu C, Chen X, et al Vitamin C inhibits the metabolic changes induced by Tet1 insufficiency under high fat diet stress. Mol Nutr Food Res. 2021;65:e2100417. 10.1002/mnfr.202100417 34129274

[153] Hou Y , Zhang Z, Wang Y, et al 5mC profiling characterized TET2 as an anti-adipogenic demethylase. Gene. 2020;733:144265. 10.1016/j.gene.2019.144265 31805318

[154] Park YJ , Han SM, Huh JY, Kim JB. Emerging roles of epigenetic regulation in obesity and metabolic disease. J Biol Chem. 2021;297:101296. 10.1016/j.jbc.2021.101296 34637788 PMC8561000

[155] Byun S , Lee CH, Jeong H, et al Loss of adipose TET proteins enhances β-adrenergic responses and protects against obesity by epigenetic regulation of β3-AR expression. Proc Natl Acad Sci USA. 2022;119:e2205626119. 10.1073/pnas.2205626119 35737830 PMC9245707

[156] Kwon H , Pessin JE. Adipokines mediate inflammation and insulin resistance. Front Endocrinol (Lausanne). 2013;4:71. 10.3389/fendo.2013.00071 23781214 PMC3679475

[157] McLaughlin T , Ackerman SE, Shen L, Engleman E. Role of innate and adaptive immunity in obesity-associated metabolic disease. J Clin Invest. 2017;127:5-13. 28045397 10.1172/JCI88876 PMC5199693

[158] Yanai H , Yoshida H. Beneficial effects of adiponectin on glucose and lipid metabolism and atherosclerotic progression: mechanisms and perspectives. Int J Mol Sci. 2019;20:1190. 10.3390/ijms20051190 PMC6429491 30857216

[159] Kadowaki T , Yamauchi T. Adiponectin and adiponectin receptors. Endocr Rev. 2005;26:439-451. 10.1210/er.2005-0005 15897298

[160] Johnston CS , Beezhold BL, Mostow B, Swan PD. Plasma vitamin C is inversely related to body mass index and waist circumference but not to plasma adiponectin in nonsmoking adults. J Nutr. 2007;137:1757-1762. 10.1093/jn/137.7.1757 17585027

[161] Vissers MCM , Gunningham SP, Morrison MJ, Dachs GU, Currie MJ. Modulation of hypoxia-inducible factor-1 alpha in cultured primary cells by intracellular ascorbate. Free Radic Biol Med. 2007;42:765-772. J.freeradbiomed.2006.11.023 17320759 10.1016/j.freeradbiomed.2006.11.023

[162] He Q , Gao Z, Yin J, Zhang J, Yun Z, Ye J. Regulation of HIF-1{alpha} activity in adipose tissue by obesity-associated factors: adipogenesis, insulin, and hypoxia. Am J Physiol Endocrinol Metab. 2011;300:E877-85. 10.1152/ajpendo.00626.2010 21343542 PMC3093977

[163] Gabryelska A , Karuga FF, Szmyd B, Białasiewicz P. HIF-1α as a mediator of insulin resistance, T2DM, and its complications: potential links with obstructive sleep apnea. Front Physiol. 2020;11:1035. 10.3389/fphys.2020.01035 33013447 PMC7509176

[164] Jiang C , Kim JH, Li F, et al Hypoxia-inducible factor 1α regulates a SOCS3-STAT3-adiponectin signal transduction pathway in adipocytes. J Biol Chem. 2013;288:3844-3857. 10.1074/jbc.M112.426338 23255598 PMC3567639

[165] Amitani M , Asakawa A, Amitani H, Inui A. The role of leptin in the control of insulin-glucose axis. Front Neurosci. 2013;7:51. 10.3389/fnins.2013.00051 23579596 PMC3619125

[166] Park S , Aintablian A, Coupe B, Bouret SG. The endoplasmic reticulum stress-autophagy pathway controls hypothalamic development and energy balance regulation in leptin-deficient neonates. Nat Commun. 2020;11:1914. 10.1038/s41467-020-15624-y 32313051 PMC7171135

[167] Thon M , Hosoi T, Ozawa K. Dehydroascorbic acid-induced endoplasmic reticulum stress and leptin resistance in neuronal cells. Biochem Biophys Res Commun. 2016;478:716-720. 10.1016/j.bbrc.2016.08.013 27498033

[168] Rudich A , Tirosh A, Potashnik R, Hemi R, Kanety H, Bashan N. Prolonged oxidative stress impairs insulin-induced GLUT4 translocation in 3T3-L1 adipocytes. Diabetes. 1998;47:1562-1569. 10.2337/diabetes.47.10.1562 9753293

[169] Bouviere J , Fortunato RS, Dupuy C, Werneck-de-Castro JP, Carvalho DP, Louzada RA. Exercise-stimulated ROS sensitive signaling pathways in skeletal muscle. Antioxidants (Basel). 2021;10:537. 10.3390/antiox10040537 PMC8066165 33808211

[170] Shi L , Du X, Guo P, Huang L, Qi P, Gong Q. Ascorbic acid supplementation in type 2 diabetes mellitus: a protocol for systematic review and meta-analysis. Medicine (Baltimore). 2020;99:e23125. 10.1097/md.0000000000023125 33157992 PMC7647560

[171] Liang Y , Kaushal D, Wilson RB. Cellular senescence and extracellular vesicles in the pathogenesis and treatment of obesity—a narrative review. Int J Mol Sci. 2024;25:7943. 10.3390/ijms25147943 PMC11276987 39063184

[172] Jung CH , Wells WW. Ascorbic acid is a stimulatory cofactor for mitochondrial glycerol-3-phosphate dehydrogenase. Biochem Biophys Res Commun. 1997;239:457-462. 10.1006/bbrc.1997.7438 9344851

[173] Talwar D , Miller CG, Grossmann J, et al The GAPDH redox switch safeguards reductive capacity and enables survival of stressed tumour cells. Nat Metab. 2023;5:660-676. 10.1038/s42255-023-00781-3 37024754 PMC10132988

[174] Napolitano G , Fasciolo G, Venditti P. Mitochondrial management of reactive oxygen species. Antioxidants (Basel). 2021;10:1824. 10.3390/antiox10111824 PMC8614740 34829696

[175] Yan LJ. Pathogenesis of chronic hyperglycemia: from reductive stress to oxidative stress. J Diabetes Res. 2014;2014:137919. 10.1155/2014/137919 25019091 PMC4082845

[176] Kondo Y , Ishigami A. Involvement of senescence marker protein-30 in glucose metabolism disorder and non-alcoholic fatty liver disease. Geriatrics Gerontol Int. 2016;16(Suppl 1):4-16. 10.1111/ggi.12722 27018279

[177] Johnson RJ , Lanaspa MA, Sanchez-Lozada LG, et al The fructose survival hypothesis for obesity. Philos Trans R Soc Lond B Biol Sci. 2023;378:20220230. 10.1098/rstb.2022.0230 37482773 PMC10363705

[178] Rahangdale S , Yeh SY, Malhotra A, Veves A. Therapeutic interventions and oxidative stress in diabetes. Front Biosci (Landmark Ed). 2009;14:192-209. 10.2741/3240 19273063 PMC5496818

[179] Caturano A , D’Angelo M, Mormone A, et al Oxidative stress in type 2 diabetes: impacts from pathogenesis to lifestyle modifications. Curr Issues Mol Biol. 2023;45:6651-6666. 10.3390/cimb45080420 37623239 PMC10453126

[180] Srikanth KK , Orrick JA. Biochemistry, Polyol or Sorbitol Pathways. StatPearls Publishing LLC; 2025. Accessed January 3, 2025. https://pubmed.ncbi.nlm.nih.gov/35015406/35015406

[181] Liu M , Park S. A causal relationship between vitamin C intake with hyperglycemia and metabolic syndrome risk: a two-sample Mendelian randomization study. Antioxidants (Basel). 2022;11:857. 10.3390/antiox11050857 PMC9137888 35624721

[182] Turpin C , Catan A, Guerin-Dubourg A, et al Enhanced oxidative stress and damage in glycated erythrocytes. PLoS One. 2020;15:e0235335. 10.1371/journal.pone.0235335 32628695 PMC7337333

[183] Lundy C , Fessler SN, Johnston CS. Erythrocyte osmotic fragility is not linked to vitamin C nutriture in adults with well-controlled type 2 diabetes. Front Nutr. 2022;9:954010. 10.3389/fnut.2022.954010 36034913 PMC9412951

[184] Williams A , Bissinger R, Shamaa H, et al Pathophysiology of red blood cell dysfunction in diabetes and its complications. Pathophysiology. 2023;30:327-345. 10.3390/pathophysiology30030026 37606388 PMC10443300

[185] Kahleova H , Matoulek M, Malinska H, et al Vegetarian diet improves insulin resistance and oxidative stress markers more than conventional diet in subjects with type 2 diabetes. Diabet Med. 2011;28:549-559. 10.1111/j.1464-5491.2010.03209.x 21480966 PMC3427880

[186] Kim Y , Oh YK, Lee J, Kim E. Could nutrient supplements provide additional glycemic control in diabetes management? A systematic review and meta-analysis of randomized controlled trials of as an add-on nutritional supplementation therapy. Arch Pharm Res. 2022;45:185-204. 10.1007/s12272-022-01374-6 35304727

[187] Ashor AW , Werner AD, Lara J, Willis ND, Mathers JC, Siervo M. Effects of vitamin C supplementation on glycaemic control: a systematic review and meta-analysis of randomised controlled trials. Eur J Clin Nutr. 2017;71:1371-1380. 10.1038/ejcn.2017.24 28294172

[188] Song Y , Xu Q, Park Y, Hollenbeck A, Schatzkin A, Chen H. Multivitamins, individual vitamin and mineral supplements, and risk of diabetes among older U.S. adults. Diabetes Care. 2011;34:108-114. 10.2337/dc10-1260 20978095 PMC3005464

[189] Mason SA , Della Gatta PA, Snow RJ, Russell AP, Wadley GD. Ascorbic acid supplementation improves skeletal muscle oxidative stress and insulin sensitivity in people with type 2 diabetes: findings of a randomized controlled study. Free Radic Biol Med. 2016;93:227-238. 10.1016/j.freeradbiomed.2016.01.006 26774673

[190] Jiang C-L , Tsao C-Y, Lee Y-C. Vitamin C attenuates predisposition to high-fat diet-induced metabolic dysregulation in GLUT10-deficient mouse model. Genes Nutr. 2022;17:10. 10.1186/s12263-022-00713-y 35842612 PMC9288715

[191] Boel A , Veszelyi K, Németh CE, et al Arterial tortuosity syndrome: an ascorbate compartmentalization disorder? Antioxid Redox Signal. 2021;34:875-889. 10.1089/ars.2019.7843 31621376

[192] Vázquez-Meza H , Vilchis-Landeros MM, Vázquez-Carrada M, Uribe-Ramírez D, Matuz-Mares D. Cellular compartmentalization, glutathione transport and its relevance in some pathologies. Antioxidants (Basel). 2023;12:834. 10.3390/antiox12040834 PMC10135322 37107209

[193] Lin IC , Yang Y-W, Wu M-F, et al The association of metabolic syndrome and its factors with gallstone disease. BMC Fam Pract. 2014;15:138. 10.1186/1471-2296-15-138 25070766 PMC4118643

[194] Zhu Q , Xing Y, Fu Y, et al Causal association between metabolic syndrome and cholelithiasis: a Mendelian randomization study. Front Endocrinol (Lausanne). 2023;14:1180903. 10.3389/fendo.2023.1180903 37361524 PMC10288183

[195] Ginter E. Cholesterol: vitamin C controls its transformation to bile acids. Science. 1973;179:702-704. 4685043 10.1126/science.179.4074.702

[196] Chiang JYL , Ferrell JM. Discovery of farnesoid X receptor and its role in bile acid metabolism. Mol Cell Endocrinol. 2022;548:111618. 10.1016/j.mce.2022.111618 35283218 PMC9038687

[197] Hemilä H. Vitamin C and plasma cholesterol. Crit Rev Food Sci Nutr. 1992;32:33-57. 10.1080/10408399209527579 1290584

[198] Pullinger CR , Eng C, Salen G, et al Human cholesterol 7alpha-hydroxylase (CYP7A1) deficiency has a hypercholesterolemic phenotype. J Clin Invest. 2002;110:109-117. 10.1172/jci15387 12093894 PMC151029

[199] Vargas-Alarcón G , Pérez-Méndez Ó, Posadas-Sánchez R, et al Associations of the CYP7A1 gene polymorphisms located in the promoter and enhancer regions with the risk of acute coronary syndrome, plasma cholesterol, and the incidence of diabetes. Biomedicines. 2024;12:617. 10.3390/biomedicines12030617 PMC10968401 38540230

[200] Frikke-Schmidt H , Lykkesfeldt J. Role of marginal vitamin C deficiency in atherogenesis: in vivo models and clinical studies. Basic Clin Pharmacol Toxicol. 2009;104:419-433. 10.1111/j.1742-7843.2009.00420.x 19489786

[201] Ginter E. Vitamin C deficiency, cholesterol metabolism, and atherosclerosis. J Orthomolec Med. 1991;6:166-183.

[202] Pendyala S , Walker JM, Holt PR. A high-fat diet is associated with endotoxemia that originates from the gut. Gastroenterology. 2012;142:1100-1101, e2. 10.1053/j.gastro.2012.01.034 22326433 PMC3978718

[203] Nogal A , Valdes AM, Menni C. The role of short-chain fatty acids in the interplay between gut microbiota and diet in cardio-metabolic health. Gut Microbes. 2021;13:1-24. 10.1080/19490976.2021.1897212 PMC8007165 33764858

[204] Carlson JL , Erickson JM, Lloyd BB, Slavin JL. Health effects and sources of prebiotic dietary fiber. Curr Dev Nutr. 2018;2:nzy005. 10.1093/cdn/nzy005 30019028 PMC6041804

[205] Fei N , Bruneau A, Zhang X, et al Endotoxin producers overgrowing in human gut microbiota as the causative agents for nonalcoholic fatty liver disease. mBio. 2020;11:e03263-19. 10.1128/mBio.03263-19 PMC7002352 32019793

[206] Mohammad S , Thiemermann C. Role of metabolic endotoxemia in systemic inflammation and potential interventions. Front Immunol. 2020;11:594150. 10.3389/fimmu.2020.594150 33505393 PMC7829348

[207] Fusco W , Lorenzo MB, Cintoni M, et al Short-chain fatty-acid-producing bacteria: key components of the human gut microbiota. Nutrients. 2023;15:2211. 10.3390/nu15092211 PMC10180739 37432351

[208] McBurney MI , Cho CE. Understanding the role of the human gut microbiome in overweight and obesity. Ann N Y Acad Sci. 2024;1540:61-88. 10.1111/nyas.15215 39283061

[209] Pham VT , Dold S, Rehman A, Bird JK, Steinert RE. Vitamins, the gut microbiome and gastrointestinal health in humans. Nutr Res. 2021;95:35-53. 10.1016/j.nutres.2021.09.001 34798467

[210] Mani V , Hollis JH, Gabler NK. Dietary oil composition differentially modulates intestinal endotoxin transport and postprandial endotoxemia. Nutr Metab (Lond). 2013;10:6. 10.1186/1743-7075-10-6 23305038 PMC3577458

[211] Ogilvie AR , Onishi JC, Schlussel Y, et al Short-term high fat diet-induced metabolic endotoxemia in older individuals with obesity: a randomized crossover study. Am J Clin Nutr. 2025;122:601-611. 10.1016/j.ajcnut.2025.06.001 40480607 PMC12405786

[212] Portincasa P , Bonfrate L, Khalil M, et al Intestinal barrier and permeability in health, obesity and NAFLD. Biomedicines. 2021;10:83. 10.3390/biomedicines10010083 PMC8773010 35052763

[213] Traber MG , Buettner GR, Bruno RS. The relationship between vitamin C status, the gut-liver axis, and metabolic syndrome. Redox Biol. 2019;21:101091. 10.1016/j.redox.2018.101091 30640128 PMC6327911

[214] Kessoku T , Kobayashi T, Imajo K, et al Endotoxins and non-alcoholic fatty liver disease. Front Endocrinol (Lausanne). 2021;12:770986. 10.3389/fendo.2021.770986 34777261 PMC8586459

[215] Bao X , Liang Y, Chang H, et al Targeting proprotein convertase subtilisin/kexin type 9 (PCSK9): from bench to bedside. Signal Transduct Target Ther. 2024;9:13. 10.1038/s41392-023-01690-3 38185721 PMC10772138

[216] Boyd JH , Fjell CD, Russell JA, Sirounis D, Cirstea MS, Walley KR. Increased plasma PCSK9 levels are associated with reduced endotoxin clearance and the development of acute organ failures during sepsis. J Innate Immun. 2016;8:211-220. 10.1159/000442976 26756586 PMC6738828

[217] Lee H , Ahn J, Shin SS, Yoon M. Ascorbic acid inhibits visceral obesity and nonalcoholic fatty liver disease by activating peroxisome proliferator-activated receptor α in high-fat-diet-fed C57BL/6J mice. Int J Obes. 2019;43:1620-1630. 10.1038/s41366-018-0212-0 30283077

[218] Ducheix S , Montagner A, Theodorou V, Ferrier L, Guillou H. The liver X receptor: a master regulator of the gut–liver axis and a target for non alcoholic fatty liver disease. Biochem Pharmacol. 2013;86:96-105. 23542537 10.1016/j.bcp.2013.03.016

[219] Grewal T , Buechler C. Emerging insights on the diverse roles of proprotein convertase subtilisin/kexin type 9 (PCSK9) in chronic liver diseases: cholesterol metabolism and beyond. Int J Mol Sci. 2022;23:1070. 10.3390/ijms23031070 PMC8834914 35162992

[220] Han L , Wu L, Yin Q, et al A promising therapy for fatty liver disease: PCSK9 inhibitors. Phytomedicine. 2024;128:155505. 10.1016/j.phymed.2024.155505 38547616

[221] Sokal-Dembowska A , Jarmakiewicz-Czaja S, Ferenc K, Filip R. Can nutraceuticals support the treatment of MASLD/MASH, and thus affect the process of liver fibrosis? Int J Mol Sci. 2024;25:5238. 38791276 10.3390/ijms25105238 PMC11120776

[222] Wu H , Guo J-L, Yao J-J, et al Serum vitamin C levels and risk of non-alcoholic fatty liver disease: results from a cross-sectional study and Mendelian randomization analysis. Front Nutr. 2023;10:1162031. 10.3389/fnut.2023.1162031 37252248 PMC10213341

[223] Zhang H , Zhang E, Hu H. Role of ferroptosis in non-alcoholic fatty liver disease and its implications for therapeutic strategies. Biomedicines. 2021;9:1660. 10.3390/biomedicines9111660 PMC8615581 34829889

[224] Li N , Zhao G, Wu W, et al The efficacy and safety of vitamin C for iron supplementation in adult patients with iron deficiency anemia: a randomized clinical trial. JAMA Netw Open. 2020;3:e2023644. 10.1001/jamanetworkopen.2020.23644 33136134 PMC7607440

[225] Lane DJ , Richardson DR. The active role of vitamin C in mammalian iron metabolism: much more than just enhanced iron absorption!. Free Radic Biol Med. 2014;75:69-83. 10.1016/j.freeradbiomed.2014.07.007 25048971

[226] Badu-Boateng C , Naftalin RJ. Ascorbate and ferritin interactions: consequences for iron release in vitro and in vivo and implications for inflammation. Free Radic Biol Med. 2019;133:75-87. 10.1016/j.freeradbiomed.2018.09.041 30268889

[227] Li J , Zheng S, Fan Y, Tan K. Emerging significance and therapeutic targets of ferroptosis: a potential avenue for human kidney diseases. Cell Death Dis. 2023;14:628. 10.1038/s41419-023-06144-w 37739961 PMC10516929

[228] Szarka A , Kapuy O, Lőrincz T, Bánhegyi G. Vitamin C and cell death. Antioxid Redox Signal. 2021;34:831-844. 10.1089/ars.2019.7897 32586104

[229] Timoshnikov VA , Kobzeva TV, Polyakov NE, Kontoghiorghes GJ. Redox interactions of vitamin C and iron: inhibition of the pro-oxidant activity by deferiprone. Int J Mol Sci. 2020;21:3967. 32486511 10.3390/ijms21113967 PMC7312906

[230] Michels AJ , Frei B. Myths, artifacts, and fatal flaws: identifying limitations and opportunities in vitamin C research. Nutrients. 2013;5:5161-5192. 10.3390/nu5125161 24352093 PMC3875932

[231] Fujita N , Takei Y. Iron overload in nonalcoholic steatohepatitis. Adv Clin Chem. 2011;55:105-132. 22126026 10.1016/b978-0-12-387042-1.00006-x

[232] Hu Z , Li Y, Ma B, Lei S, Wang X. Iron metabolism mediates the relationship between Vitamin C and hepatic steatosis and fibrosis in NAFLD. Front Nutr. 2022;9:952056. 10.3389/fnut.2022.952056 36159474 PMC9494736

[233] Chen K , Suh J, Carr AC, Morrow JD, Zeind J, Frei B. Vitamin C suppresses oxidative lipid damage in vivo, even in the presence of iron overload. Am J Physiol Endocrinol Metab. 2000;279:E1406-12. 10.1152/ajpendo.2000.279.6.E1406 11093930

[234] National Institute of Diabetes and Digestive and Kidney Diseases. LiverTox: clinical and research information on drug-induced liver injury. Accessed January 3, 2025. https://www.ncbi.nlm.nih.gov/books/NBK548448/#:∼:text=Introduction,acute%20liver%20injury%20or%20jaundice 31643176

[235] Koop AC , Thiele ND, Steins D, et al Therapeutic targeting of myeloperoxidase attenuates NASH in mice. Hepatol Commun. 2020;4:1441-1458. 10.1002/hep4.1566 33024915 PMC7527691

[236] Andrés CMC , Pérez de la Lastra JM, Juan CA, Plou FJ, Pérez-Lebeña E. Hypochlorous acid chemistry in mammalian cells-influence on infection and role in various pathologies. Int J Mol Sci. 2022;23:735. 10.3390/ijms231810735 PMC9504810 36142645

[237] Tangeten C , Zouaoui Boudjeltia K, Delporte C, Van Antwerpen P, Korpak K. Unexpected role of MPO-oxidized LDLs in atherosclerosis: in between inflammation and its resolution. Antioxidants (Basel). 2022;11:874. 10.3390/antiox11050874 PMC9137493 35624738

[238] Rensen SS , Slaats Y, Nijhuis J, et al Increased hepatic myeloperoxidase activity in obese subjects with nonalcoholic steatohepatitis. Am J Pathol. 2009;175:1473-1482. 10.2353/ajpath.2009.080999 19729473 PMC2751544

[239] Libby P. Inflammation in atherosclerosis. Arterioscler Thromb Vasc Biol. 2012;32:2045-2051. 10.1161/atvbaha.108.179705 22895665 PMC3422754

[240] Chistiakov DA , Bobryshev YV, Orekhov AN. Macrophage-mediated cholesterol handling in atherosclerosis. J Cell Mol Med. 2016;20:17-28. 10.1111/jcmm.12689 26493158 PMC4717859

[241] Kiokias S , Proestos C, Oreopoulou V. Effect of natural food antioxidants against LDL and DNA oxidative changes. Antioxidants (Basel). 2018;7:133. 10.3390/antiox7100133 PMC6211048 30282925

[242] Das S , Das N, Srivastava LM, Snehlata. Role of ascorbic acid on in vitro oxidation of low-density lipoprotein derived from hypercholesterolemic patients. Clin Chim Acta. 2006;372:202-205. 10.1016/j.cca.2006.03.016 16701604

[243] Carr AC , McCall MR, Frei B. Oxidation of LDL by myeloperoxidase and reactive nitrogen species: reaction pathways and antioxidant protection. Arterioscler Thromb Vasc Biol. 2000;20:1716-1723. 10.1161/01.atv.20.7.1716 10894808

[244] Du J , Cullen JJ, Buettner GR. Ascorbic acid: chemistry, biology and the treatment of cancer. Biochim Biophys Acta. 2012;1826:443-457. 22728050 10.1016/j.bbcan.2012.06.003 PMC3608474

[245] Toh JWT , Wilson RB. Pathways of gastric carcinogenesis, helicobacter pylori virulence and interactions with antioxidant systems, vitamin C and phytochemicals. Int J Mol Sci. 2020;21:6451. 10.3390/ijms21176451 PMC7503565 32899442

[246] Di Salvo E , Casciaro M, Giorgianni CM, Cicero N, Gangemi S. Age-related diseases and foods generating chlorinative stress. Antioxidants (Basel). 2023;12:249. 10.3390/antiox12020249 PMC9952263 36829808

[247] Liu X , Shen H, Chen M, Shao J. Clinical relevance of vitamins and carotenoids with liver steatosis and fibrosis detected by transient elastography in adults. Front Nutr. 2021;8:760985. 10.3389/fnut.2021.760985 34869532 PMC8632634

[248] Zhao Y , Li H. Association of serum vitamin C with liver fibrosis in adults with nonalcoholic fatty liver disease. Scand J Gastroenterol. 2022;57:872-877. 10.1080/00365521.2022.2041085 35189786

[249] Wei J , Lei G-h, Fu L, Zeng C, Yang T, Peng S-f. Association between dietary vitamin c intake and non-alcoholic fatty liver disease: a cross-sectional study among middle-aged and older adults. PLoS One. 2016;11:e0147985. 10.1371/journal.pone.0147985 26824361 PMC4732670

[250] Fabbrini E , Sullivan S, Klein S. Obesity and nonalcoholic fatty liver disease: biochemical, metabolic, and clinical implications. Hepatology. 2010;51:679-689. 10.1002/hep.23280 20041406 PMC3575093

[251] Ivancovsky-Wajcman D , Fliss-Isakov N, Salomone F, et al Dietary vitamin E and C intake is inversely associated with the severity of nonalcoholic fatty liver disease. Digest Liver Dis. 2019;51:1698-1705. 10.1016/j.dld.2019.06.005 31281067

[252] Ezhilarasan D , Lakshmi T. A molecular insight into the role of antioxidants in nonalcoholic fatty liver diseases. Oxid Med Cell Longev. 2022;2022:9233650. 10.1155/2022/9233650 35602098 PMC9117022

[253] Foster T , Budoff MJ, Saab S, Ahmadi N, Gordon C, Guerci AD. Atorvastatin and antioxidants for the treatment of nonalcoholic fatty liver disease: the St Francis Heart Study randomized clinical trial. Am J Gastroenterol. 2011;106:71-77. 10.1038/ajg.2010.299 20842109

[254] Carr AC , Vlasiuk E, Zawari M, Lunt H. Understanding the additional impact of prediabetes and type 2 diabetes mellitus on vitamin C requirements in people living with obesity. Nutr Res. 2024;130:1. 10.1016/j.nutres.2024.08.001 39303359

[255] Carr AC , Pullar JM, Bozonet SM, Vissers MC. Marginal ascorbate status (hypovitaminosis C) results in an attenuated response to vitamin C supplementation. Nutrients. 2016;8:341. 10.3390/nu8060341 PMC4924182 27271663

[256] Farag HAM , Hosseinzadeh-Attar MJ, Muhammad BA, Esmaillzadeh A, El Bilbeisi AH. Effects of vitamin C supplementation with and without endurance physical activity on components of metabolic syndrome: a randomized, double-blind, placebo-controlled clinical trial. Clin Nutr Exp. 2019;26:23-33. 10.1016/j.yclnex.2019.05.003

[257] Chen H , Karne RJ, Hall G, et al High-dose oral vitamin C partially replenishes vitamin C levels in patients with type 2 diabetes and low vitamin C levels but does not improve endothelial dysfunction or insulin resistance. Am J Physiol Heart Circ Physiol. 2006;290:H137-H145. 16126809 10.1152/ajpheart.00768.2005

[258] Lambadiari V , Triantafyllou K, Dimitriadis GD. Insulin action in muscle and adipose tissue in type 2 diabetes: the significance of blood flow. World J Diabetes. 2015;6:626-633. 10.4239/wjd.v6.i4.626 25987960 PMC4434083

[259] McRae MP. Vitamin C supplementation lowers serum low-density lipoprotein cholesterol and triglycerides: a meta-analysis of 13 randomized controlled trials. J Chiropr Med. 2008;7:48-58. 10.1016/j.jcme.2008.01.002 19674720 PMC2682928

[260] Ashor AW , Siervo M, van der Velde F, Willis ND, Mathers JC. Systematic review and meta-analysis of randomised controlled trials testing the effects of vitamin C supplementation on blood lipids. Clin Nutr. 2016;35:626-637. 10.1016/j.clnu.2015.05.021 26164552

[261] Cicero AFG , Fogacci F, Bove M, Giovannini M, Borghi C. Three-arm, placebo-controlled, randomized clinical trial evaluating the metabolic effect of a combined nutraceutical containing a bergamot standardized flavonoid extract in dyslipidemic overweight subjects. Phytother Res. 2019;33:2094-2101. 10.1002/ptr.6402 31225673

[262] Murer SB , Aeberli I, Braegger CP, et al Antioxidant supplements reduced oxidative stress and stabilized liver function tests but did not reduce inflammation in a randomized controlled trial in obese children and adolescents. J Nutr. 2014;144:193-201. 10.3945/jn.113.185561 24353344

[263] Vincent HK , Bourguignon CM, Vincent KR, Weltman AL, Bryant M, Taylor AG. Antioxidant supplementation lowers exercise-induced oxidative stress in young overweight adults. Obesity (Silver Spring). 2006;14:2224-2235. 10.1038/oby.2006.261 17189550

[264] Li S , Fasipe B, Laher I. Potential harms of supplementation with high doses of antioxidants in athletes. J Exerc Sci Fit. 2022;20:269-275. 10.1016/j.jesf.2022.06.001 35812825 PMC9241084

[265] McMorrow AM , Connaughton RM, Magalhães TR, et al Personalized cardio-metabolic responses to an anti-inflammatory nutrition intervention in obese adolescents: a randomized controlled crossover trial. Mol Nutr Food Res. 2018;62:e1701008. 10.1002/mnfr.201701008 29665620 PMC6079645

[266] Czernichow S , Vergnaud AC, Galan P, et al Effects of long-term antioxidant supplementation and association of serum antioxidant concentrations with risk of metabolic syndrome in adults. Am J Clin Nutr. 2009;90:329-335. 10.3945/ajcn.2009.27635 19491388

[267] Yang Y , Xu H, Zhang Y, et al Associations of dietary antioxidant micronutrients with the prevalence of obesity in adults. Front Nutr. 2023;10:1098761. 10.3389/fnut.2023.1098761 36992905 PMC10040542

[268] Huang PL. A comprehensive definition for metabolic syndrome. Dis Model Mech. 2009;2:231-237. 10.1242/dmm.001180 19407331 PMC2675814

[269] Lira AB , de Sousa Rodrigues CF. Evaluation of oxidative stress markers in obstructive sleep apnea syndrome and additional antioxidant therapy: a review article. Sleep Breath. 2016;20:1155-1160. 10.1007/s11325-016-1367-3 27255237

[270] Perticone F , Ceravolo R, Candigliota M, et al Obesity and body fat distribution induce endothelial dysfunction by oxidative stress: protective effect of vitamin C. Diabetes. 2001;50:159-165. 10.2337/diabetes.50.1.159 11147782

[271] Ariel H , Cooke JP. Cardiovascular risk of proton pump inhibitors. Methodist Debakey Cardiovasc J. 2019;15:214-219. 10.14797/mdcj-15-3-214 31687101 PMC6822659

[272] Ghebremariam YT , LePendu P, Lee JC, et al Unexpected effect of proton pump inhibitors: elevation of the cardiovascular risk factor asymmetric dimethylarginine. Circulation. 2013;128:845-853. 10.1161/circulationaha.113.003602 23825361 PMC3838201

[273] Kobayashi J , Uchida H, Ito J. Long-term proton pump inhibitor use after Helicobacter pylori eradication may create a gastric environment for N-nitrosamine formation and gastric cancer development. Gut. 2019;68:1131. 10.1136/gutjnl-2018-316592 29802173

[274] Bryan NS. Nitric oxide enhancement strategies. Future Sci OA. 2015;1:Fso48. 10.4155/fso.15.48 28031863 PMC5137939

[275] Bryan NS. Functional nitric oxide nutrition to combat cardiovascular disease. Curr Atheroscler Rep. 2018;20:21. 10.1007/s11883-018-0723-0 29550903

[276] Łuczak A , Madej M, Kasprzyk A, Doroszko A. Role of the eNOS uncoupling and the nitric oxide metabolic pathway in the pathogenesis of autoimmune rheumatic diseases. Oxid Med Cell Longev. 2020;2020:1417981. 10.1155/2020/1417981 32351667 PMC7174952

[277] Hartley L , Igbinedion E, Holmes J, et al Increased consumption of fruit and vegetables for the primary prevention of cardiovascular diseases. Cochrane Database Syst Rev. 2013;2013:CD009874. 10.1002/14651858.CD009874.pub2 23736950 PMC6464871

[278] Lbban E , Kwon K, Ashor A, et al Vitamin C supplementation showed greater effects on systolic blood pressure in hypertensive and diabetic patients: an updated systematic review and meta-analysis of randomised clinical trials. Int J Food Sci Nutr. 2023;74:814-825. 10.1080/09637486.2023.2264549 37791386

[279] Madsen H , Sen A, Aune D. Fruit and vegetable consumption and the risk of hypertension: a systematic review and meta-analysis of prospective studies. Eur J Nutr. 2023;62:1941-1955. 10.1007/s00394-023-03145-5 37106252 PMC10349693

[280] The Blood Pressure Lowering Treatment Trialists Collaboration. Pharmacological blood pressure lowering for primary and secondary prevention of cardiovascular disease across different levels of blood pressure: an individual participant-level data meta-analysis. Lancet. 2021;397:1625-1636. 10.1016/s0140-6736(21)00590-0 33933205 PMC8102467

[281] Cook NR , Albert CM, Gaziano JM, et al A randomized factorial trial of vitamins C and E and beta carotene in the secondary prevention of cardiovascular events in women: results from the Women’s Antioxidant Cardiovascular Study. Arch Intern Med. 2007;167:1610-1618. 10.1001/archinte.167.15.1610 17698683 PMC2034519

[282] Sesso HD , Buring JE, Christen WG, et al Vitamins E and C in the prevention of cardiovascular disease in men: the Physicians’ Health Study II randomized controlled trial. JAMA. 2008;300:2123-2133. 10.1001/jama.2008.600 18997197 PMC2586922

[283] Waters DD , Alderman EL, Hsia J, et al Effects of hormone replacement therapy and antioxidant vitamin supplements on coronary atherosclerosis in postmenopausal women: a randomized controlled trial. JAMA. 2002;288:2432-2440. 10.1001/jama.288.19.2432 12435256

[284] Bleys J , Miller ER 3rd, Pastor-Barriuso R, Appel LJ, Guallar E. Vitamin-mineral supplementation and the progression of atherosclerosis: a meta-analysis of randomized controlled trials. Am J Clin Nutr. 2006;84:880.7; quiz 954-5. 10.1093/ajcn/84.4.880 17023716

[285] Shekelle P , Morton S, Hardy ML. Effect of supplemental antioxidants vitamin C, vitamin E, and coenzyme Q10 for the prevention and treatment of cardiovascular disease. Evid Rep Technol Assess (Summ). 2003;83:1-3. PMC4781262 15040141

[286] Lee DH , Folsom AR, Harnack L, Halliwell B, Jacobs DR. Jr. Does supplemental vitamin C increase cardiovascular disease risk in women with diabetes? Am J Clin Nutr. 2004;80:1194-1200. 10.1093/ajcn/80.5.1194 15531665

[287] Kurnick A , Zaveri S, Tadayoni A, Chandrakumar HP, John S. Reversible severe pulmonary hypertension and right heart failure with cardiogenic shock due to scurvy: a case report. Eur Heart J Case Rep. 2023;7: Ytad404. 10.1093/ehjcr/ytad404 37650076 PMC10464571

[288] Hemilä H , de Man AME. Vitamin C deficiency can lead to pulmonary hypertension: a systematic review of case reports. BMC Pulm Med. 2024;24:140. 10.1186/s12890-024-02941-x 38504249 PMC10949735

[289] Hemilä H , Chalker E, de Man AME. Vitamin C may improve left ventricular ejection fraction: a meta-analysis. Front Cardiovasc Med. 2022;9:789729. 10.3389/fcvm.2022.789729 35282368 PMC8913583

[290] Polidori MC , Praticó D, Savino K, Rokach J, Stahl W, Mecocci P. Increased F2 isoprostane plasma levels in patients with congestive heart failure are correlated with antioxidant status and disease severity. J Card Fail. 2004;10:334-338. 10.1016/j.cardfail.2003.11.004 15309701

[291] Reynolds A , Mann J, Cummings J, Winter N, Mete E, Te Morenga L. Carbohydrate quality and human health: a series of systematic reviews and meta-analyses. Lancet. 2019;393:434-445. 10.1016/s0140-6736(18)31809-9 30638909

[292] Tobias DK , Chen M, Manson JE, Ludwig DS, Willett W, Hu FB. Effect of low-fat diet interventions versus other diet interventions on long-term weight change in adults: a systematic review and meta-analysis. Lancet Diabetes Endocrinol. 2015;3:968-979. 10.1016/s2213-8587(15)00367-8 26527511 PMC4667723

[293] Mansoor N , Vinknes KJ, Veierød MB, Retterstøl K. Effects of low-carbohydrate diets v. low-fat diets on body weight and cardiovascular risk factors: a meta-analysis of randomised controlled trials. Br J Nutr. 2016;115:466-479. 10.1017/s0007114515004699 26768850

[294] Parmar RM , Can AS. Dietary Approaches to Obesity Treatment. StatPearls; 2023. 34662090

[295] Esposito K , Kastorini CM, Panagiotakos DB, Giugliano D. Mediterranean diet and weight loss: meta-analysis of randomized controlled trials. Metab Syndr Relat Disord. 2011;9:1-12. 10.1089/met.2010.0031 20973675

[296] Appel LJ , Moore TJ, Obarzanek E, et al A clinical trial of the effects of dietary patterns on blood pressure. N Engl J Med. 1997;336:1117-1124. 9099655 10.1056/NEJM199704173361601

[297] Soltani S , Shirani F, Chitsazi MJ, Salehi‐Abargouei A. The effect of Dietary Approaches to Stop Hypertension (DASH) diet on weight and body composition in adults: a systematic review and meta‐analysis of randomized controlled clinical trials. Obes Rev. 2016;17:442-454. 26990451 10.1111/obr.12391

[298] Nobili V , Manco M, Devito R, et al Lifestyle intervention and antioxidant therapy in children with nonalcoholic fatty liver disease: a randomized, controlled trial. Hepatology. 2008;48:119-128. 10.1002/hep.22336 18537181

[299] Shenoy SF , Poston WSC, Reeves RS, et al Weight loss in individuals with metabolic syndrome given DASH diet counseling when provided a low sodium vegetable juice: a randomized controlled trial. Nutr J. 2010;9:8. 10.1186/1475-2891-9-8 20178625 PMC2841082

[300] Ponce O , Benassi R, Cesar T. Orange juice associated with a balanced diet mitigated risk factors of metabolic syndrome: a randomized controlled trial. J Nutr Intermed Metab. 2019;17:100101. 10.1016/j.jnim.2019.100101

[301] Abdali D , Samson SE, Grover AK. How effective are antioxidant supplements in obesity and diabetes? Med Princ Pract. 2015;24:201-215. 10.1159/000375305 25791371 PMC5588240

[302] Lee CY. Effects of dietary vitamins on obesity-related metabolic parameters. J Nutr Sci. 2023;12:e47. 10.1017/jns.2023.30 37123391 PMC10131053

[303] Brandt K. Commentary: vitamin c and metabolic syndrome: a meta-analysis of observational studies. general commentary. Front Nutr. 2021;8:810716. 10.3389/fnut.2021.810716 34970586 PMC8712686

[304] Wong SK , Chin KY, Ima-Nirwana S. Vitamin C: a review on its role in the management of metabolic syndrome. Int J Med Sci. 2020;17:1625-1638. 10.7150/ijms.47103 32669965 PMC7359392

[305] Miller CK. Medical nutrition therapy: still relevant in the era of pharmacotherapy for obesity care. J Acad Nutr Diet. 2025;125:595-599. 10.1016/j.jand.2024.06.222 38936770

[306] Wilding JPH , Batterham RL, Davies M, et al STEP 1 Study Group. Weight regain and cardiometabolic effects after withdrawal of semaglutide: the STEP 1 trial extension. Diabetes Obes Metab. 2022;24:1553-1564. 10.1111/dom.14725 35441470 PMC9542252

[307] Mozaffarian D , Agarwal M, Aggarwal M, et al Nutritional priorities to support GLP-1 therapy for obesity: a joint Advisory from the American College of Lifestyle Medicine, the American Society for Nutrition, the Obesity Medicine Association, and The Obesity Society. Obesity. 2025;33:1475-1503. 10.1002/oby.24336 40445127 PMC12304835

